# A High-Content RNAi Screen Identifies Ubiquitin Modifiers That Regulate TNF-Dependent Nuclear Accumulation of NF-κB

**DOI:** 10.3389/fimmu.2014.00322

**Published:** 2014-07-14

**Authors:** Brittany Fraser, Robert A. Maranchuk, Edan Foley

**Affiliations:** ^1^Department of Medical Microbiology and Immunology, University of Alberta, Edmonton, AB, Canada; ^2^Institute of Virology, University of Alberta, Edmonton, AB, Canada

**Keywords:** TNF, ubiquitin, NF-κB, JNK, apoptosis, siRNA screen, high-content

## Abstract

The mammalian tumor necrosis factor (TNF) cytokine is a central mediator of inflammatory events. Recent studies revealed a number of complex and sophisticated interactions between the TNF pathway and the enzymatic activities encoded by ubiquitin ligases and deubiquitylation enzymes. However, very little is known about the identity of the ubiquitin pathway members that control the extent of ubiquitylation in TNF responses. To address this deficit, we conducted an unbiased, high-content screen of the human ubiquitin pathway for gene products that control defining features of the cellular response to TNF. In particular, we sought to identify ubiquitin modifying enzymes that alter the ability of TNF to regulate the nuclear accumulation of nuclear factor kappa B. In this screen, we identified and validated several novel regulators of the TNF pathway. We believe these regulators constitute potential targets for pharmacological interventions that manipulate TNF-dependent inflammation.

## Introduction

The mammalian tumor necrosis factor (TNF) cytokine directs immunological responses to pathogenic microbes and is a central feature of numerous inflammatory illnesses (1). Physiological responses to TNF involve a complex series of events that include the activation, differentiation, mobilization, and extravasation of leukocytes, or the induction of cell death. Inflammation prevents the spread of microbial invaders, promotes the repair of damaged host tissues, and drives sophisticated secondary immune responses in mammals (2). Chronic inflammation drives persistent cycles of tissue destruction and repair that are implicated in pathological conditions such as rheumatoid arthritis, inflammatory bowel disease, and multiple sclerosis (3, 4). Persistent inflammation also establishes a micro-environment that favors the development and growth of cancerous lesions through the promotion of angiogenesis, proliferation, tissue invasion, and metastasis (5).

Given the critical links between cytokine signaling, immunity, and disease, there is considerable interest in the molecular pathways that translate detection of a cytokine into induction of a host response. Myeloid cells such as dendritic cells, macrophages, and mast cells release soluble TNF homotrimers in response to the molecular signatures of pathogenic invasion (6). Trimeric TNF induces dissociation of the suppressor of death domain (SODD) protein from homotrimers of the TNF receptor (TNFR) (7). The release of SODD exposes TNFR death domains that nucleate a complex arrangement of adaptor proteins, kinases, and ubiquitin ligases. Current models suggest that homotypic interactions between the death domains of the TNFR and the TNF receptor associated death domain (TRADD) (8) lead to the formation of a membrane-associated signaling complex, commonly referred to as complex I (9). Complex I contains TNFR, TRADD, the adaptor molecule receptor-interacting serine/threonine–protein kinase-1 [RIPK1 (10)], the ubiquitin ligase TNF receptor associated factor 2 [TRAF2 (11)], and the ubiquitin ligase cellular inhibitor of apoptosis proteins 1 and 2 [c-IAP1/2 (12)].

The ubiquitin ligase activities of cIAPs are essential for the recruitment of pro-inflammatory kinases to complex I. Activation of ubiquitin involves the serial transfer of ubiquitin molecules from E1 ligases to E2 ligases to terminal E3 ligases (13). E3 ligases transfer ubiquitin to substrate proteins in a number of different lysine-linked configurations. K48-linked ubiquitin chains target substrates for proteasomal destruction, while K63-linked chains modify the localization or actions of a substrate. In the TNF pathway, cIAPs drive the K63-conjugation of ubiquitin to a number of substrate proteins within complex I (14). Ubiquitin binding motifs on the TGF-beta activated kinase binding protein 2 (TAB2) integrate the transforming growth factor beta-activated kinase-1 (TAK1) kinase into complex I (15), and ubiquitin binding motifs on NF-κB essential modifier (NEMO) recruit the I-κB kinase (IKK) complex (16–18). TAB2–TAK1 transduces pro-inflammatory signals by the activation of c-Jun N-terminal kinase (JNK) and IKK phosphorylates inhibitor of kappa B (I-κB) proteins. In resting cells, I-κB binds and retains nuclear factor kappa B (NF-κB) transcription factors in the cytosol. Phosphorylation of I-κB results in the attachment of K48-linked ubiquitin proteins to I-κB by the SKP1-CUL-F-box-beta-transducin repeat containing (SCF–bTrCP) E3 ligase, leading to the proteasomal destruction of I-κB, the nuclear translocation of NF-κB, and the transcriptional induction of pro-inflammatory genes (19).

Recent studies established that K63-linked substrates in complex I recruit a novel ubiquitin ligase, the linear ubiquitin chain assembly complex (LUBAC) (20). LUBAC catalyzes the formation of a linear chain of ubiquitin proteins in which the C-terminal residue of one ubiquitin protein is covalently linked to the alpha-amino group of a second ubiquitin protein through a peptide bond. Biochemical studies demonstrated that LUBAC ubiquitylates RIPK1 and NEMO, and LUBAC contributes to the transduction of signals from complex I to NF-κB transcription factors.

Ubiquitin modification is also a central feature of the termination of TNF signals. Prolonged signals through the TNFR lead to the formation of a pro-apoptotic complex II that accompanies TNFR endocytosis (9). Complex II retains a core of TNFR, RIPK1, TRADD, and TRAF2, and recruits the pro-apoptotic adaptor protein Fas-associated death domain (FADD) and the initiator caspase, caspase-8. Caspase-8 is a proximal element in the induction of programed cell death by extrinsic cues (21). TNF signals rarely lead to the activation of caspase-8, as NF-κB induces the transcription of a number of proteins that effectively limit the extent of TNF signaling. For example, NF-κB induces the expression of the NF-κB inhibitor I-κB, the caspase-8 inhibitor FLIP_L_ (22), and the ubiquitin-editing enzyme A20 (23). A20 encodes ubiquitin ligase and deubiquitylation domains that act in a coordinated manner to disassemble complex I and target RIPK1 for destruction (24).

Given the involvement of ubiquitylation in TNF signaling and the extensive involvement of TNF in health and disease, there is a burgeoning interest in deciphering the precise relationship between the TNF and ubiquitin pathways. We present a simple, high-content assay that reliably quantifies nuclear translocation of NF-κB in cells treated with recombinant TNF. In an unbiased siRNA screen of the entire complement of deubiquitylation enzymes, and E1, E2, and E3 ubiquitin ligases in the human genome, we identified a number of enzymes that modify the extent to which TNF directs the nuclear accumulation of NF-κB. We believe that these enzymes represent potential targets for pharmacological manipulations that control inflammatory condition.

## Materials and Methods

### Cell culture

HeLa cells were cultured in Dulbecco’s modified Eagle Medium with 4.5 g/l d-glucose and l-glutamine (Gibco) and A549 cells were cultured in Dulbecco’s modified Eagle Medium/F12 (1:1) with l-glutamine and 15 mM HEPES (Gibco). Both were supplemented with 10% fetal bovine serum (Gibco) and 50 U/ml penicillin and 50 μg/ml streptomycin (Gibco). Cells were maintained in an incubator at 37°C with 5% CO_2_.

### High-content imaging

All liquid handling steps were performed with a Janus Workstation (Perkin Elmer) unless stated otherwise. Plates were treated with TNF for 30 or 120 min. Medium was aspirated from the cells and 50 μl DMEM culture medium (+FBS/−antibiotics) containing 10 ng/ml TNF-alpha was added to each well. Plates were incubated at 37°C for the appropriate amount of time. Medium containing TNF was aspirated and cells were washed with 150 μl PBS per well. Plates were rocked manually 10–15 times then placed on a shaker. PBS was manually dumped from plates and 150 μl 3.7% formaldehyde in PBS was added to each well using a multi-channel pipette. Plates were rocked manually 10–15 times and incubated at room temperature for at least 15 min without shaking. Formaldehyde was manually dumped from the plates. Plates were washed with 150 μl 1×PBS/0.1% Triton for 6 min while shaking at room temperature, four times. The wash solution was added with the Janus Workstation and dumped manually. Fifty microliters of 5% NGS in 1×PBS/0.1% Tween (blocking reagent) was added to each well and incubated for at least 1 h shaking at room temperature. Blocking reagent was manually dumped from plates. Fifty microliters of primary antibody solution {1:5000 anti-NF-κB [p65 (C-20) rabbit polyclonal from Santa Cruz, catalog no. sc-372] in 5% NGS in 1×PBS/0.1% Tween} was added to each well. Plates were incubated overnight with gentle rocking at 4°C. The primary antibody solution was aspirated. Plates were washed with 150 μl 1×PBS/0.1% Tween for 6 min while shaking at room temperature, four times. The wash solution was added with the Janus Workstation and dumped manually. Fifty microliters of Goat anti-rabbit Alexa Fluor 488 nm secondary antibody (Life Technologies, 1:1000) and Hoechst 34328 stain (Life Technologies, 1:1000) in 5% NGS in 1×PBS/0.1% Tween were added to each well. Plates were shielded from light and incubated at room temperature for at least 1 h shaking. The secondary antibody solution was dumped from plates. Plates were washed with 150 μl 1×PBS/0.1% Tween twice, then once with 1×PBS for 6 min while shaking. The wash solution was added with the Janus Workstation and dumped manually. One hundred fifty microliters of 1×PBS was added to each well.

Each plate was imaged with an Operetta High-Content Scanner (Perkin Elmer) and the number of cells with nuclear NF-κB in each well was quantified with Harmony software (Perkin Elmer). Individual cells were identified based on Hoechst staining of DNA. The cytoplasm of each cell was then identified based on the detection of NF-κB staining surrounding individual nuclei. Cells on the edges of each image were excluded from subsequent analysis, so that only completely visible cells were quantified. The intensity of the NF-κB signal in the nucleus was calculated by the Harmony software. Nuclear NF-κB was defined as an NF-κB signal that overlapped with the Hoechst stain. Based on the negative and positive controls in each plate, an intensity threshold was defined for each plate that identified cells with predominant nuclear NF-κB signals, i.e., where the bulk of the NF-κB signal overlapped with the Hoechst signal. The intensity threshold was set to >400–800 mean pixel intensity depending on the plate. The remaining wells of the plate were then automatically visualized and quantified. Between two and eight images were captured per well, depending on the cell density in the respective plates, with total cell count per well averaging 766.4. For the validation screens, 8–12 images per well were captured. The HeLa cell validation screen counted an average of 2056.3 cells per well, while the A549 cell validation screen counted an average of 2023.5 cells per well. A formula was set up in the analysis sequence to find the percentage of cells with nuclear NF-κB in each well: (number of cells with a strong nuclear NF-κB signal/total number of cells) × 100.

### RNAi screen

The siRNA screen was performed with Dharmacon siGENOME SMARTpool siRNA pools that target human ubiquitin conjugation enzymes and human deubiquitylating enzymes. Transfections were performed in duplicate for each plate at each time point assayed. All liquid handling steps were performed with a PerkinElmer Janus Automated Workstation. For transfections, 85 μl of DMEM culture medium (+FBS/−antibiotics) containing HeLa cells (1500 cells per well) or 85 μl of DMEM/F12 culture medium (+FBS/−antibiotics) containing A549 cells (2000 cells per well) were added to each well. Plates were incubated overnight at 37°C. The next day, siRNA was diluted to 200 nM (10 μl per well) in nuclease-free water in a mixing plate. Five microliters of Polyplus Interferin-HTS diluted in nuclease-free water (0.1 μl interferin-HTS + 4.9 μl nuclease-free water in each well) was added to each well containing siRNA and was mixed by slowly pipetting up and down. The transfection reagent and siRNA were incubated for 20–30 min at room temperature. Fifteen microliters of the transfection reagent–siRNA complex were mixed again by pipetting, and then added to the plates containing the cells (final siRNA concentration = 20 nM). Plates were mixed by gently tapping the sides of the plate 20–30 times and then incubated for 3 days at 37°C.

### Validation screens

The siRNA validation screens were performed using a custom plate of Dharmacon siGENOME individual siRNAs corresponding to the major hits from the original screen (Figure 6A). Each gene was tested with four distinct siRNA duplexes to test potential off-target effects from the pooled siRNA. The plating of cells and the RNAi transfections were executed as above.

### *z*-Score analysis

We used *z*-score analysis to normalize the percentage of cells with nuclear NF-κB values for the entire screen. The *z*-score assumes normal distribution and represents the standard deviation of well measurement value from the plate median for each dsRNA treatment. *z*-Scores were calculated by subtracting the sample value by the plate median value and dividing by the plate standard deviation. *z*-Scores above 1.96 or below −1.96 represent the 95% confidence interval.

### Reverse transfection of siRNA

For the orthogonal assays described in Figure 8 through Figure 11, cells were reverse transfected with individual siRNA duplexes (Dharmacon siGENOME individuals: *CUL1* D-004086-03; *TNFAIP3* D-009919-02; *USP7* D-006097-01, -02, -03, -04; *USP12* D-027148-01, -02, -03, -04; *USP49* D-005945-06, -05, -04, -02; *USP54* D-016853-17, -04, -02, -01; *FBXO36* D-018460-04, -03, -02, -01; *KMT2D* D-004828-01, -02, -03, -05) and control siRNA (Dharmacon siGENOME non-targeting siRNA pool no. 1, D-001206-13-05). HeLa: in a 6-, 12-, or 96-well plate, the diluted transfection reagent (0.028 μl Polyplus Interferin-HTS and 4.972 μl nuclease-free water) was added to each well (6-well = 100 μl; 12-well = 50 μl; 96-well = 5 μl). Ten micromolar of siRNA was diluted 1:50 in nuclease-free water (siRNA concentration = 200 nM) and was added to each well (6-well = 200 μl; 12-well = 100 μl; 96-well = 10 μl). The transfection reagent and the siRNA were combined by carefully pipetting up and down five times, and the transfection mixture was incubated at room temperature for 20–30 min. HeLa cells in DMEM culture medium (+FBS/−antibiotics) at 3 × 10^5^ cells/ml (western blot and qRT-PCR) or 3.63 × 10^4^ cells/ml (TUNEL) (6-well = 1.7 ml; 12-well = 850 μl; 96-well = 85 μl) were added to each well and were mixed with the transfection mixture by rocking the plate followed by gently tapping the sides of the plate (siRNA final concentration = 20 nM). Cells were incubated with the siRNA for 3 days at 37°C. A549: the diluted transfection reagent [6-well = 2 μl DharmaFECT 1 (Dharmacon) and 148 μl Opti-MEM (Gibco); 12-well = 4 μl DharmaFECT 1 and 296 μl Opti-MEM] was added to each well. Ten micromolar of siRNA was diluted 1:50 in Opti-MEM and was added to the transfection reagent by pipetting up and down. The transfection mixture was incubated at room temperature for 30 min. A549 cells in DMEM/F12 (+FBS/−antibiotics) at 3 × 10^5^ cells/ml were added to each well and were mixed as described above.

### Western blot analysis

Cells were reverse transfected in a 12-well plate, as described above. Following the incubation, the culture medium was aspirated from each well and replaced with 500 μl of 10 ng/ml TNF in DMEM (+FBS/−antibiotics) or a control solution of DMEM only, and the samples were incubated at 37°C for the appropriate amount of time. DMEM and TNF solutions were then aspirated. Fifty microliters of fresh lysis buffer (20 mM Tris, pH 7.5; 25 mM glycerol-3-phosphate; 150 mM NaCl; 1% Triton X-100; 2 mM Na_3_VO_4_; protease inhibitors) was quickly added to each well and samples were harvested by scraping the well with a plastic cell lifter. Lysates were added to 40 μl 2× sample buffer [62.5 mM Tris, pH 6.8; 10% (v/v) glycerol; 2% (w/v) SDS; 50 mM β-mercaptoethanol; 0.00125% (w/v) bromophenol blue] and boiled for 5 min. Proteins were separated by SDS-PAGE electrophoresis and transferred to a nitrocellulose membrane by semi-dry transfer. Membranes were blocked in blocking buffer (LI-COR Biosciences) for 1 h at room temperature and probed with antibodies against total I-κB [1:1000, cell signaling, no. 4812S, IkappaBalpha (44D4) Rabbit mAb], phosphorylated I-κB [1:1000, cell signaling, no. 9246S, P-IkappaBalpha (S32/36) (5A5) Mouse mAb], Tubulin (1:1000, DSHB, E7 mouse supernatant), phosphorylated JNK [1:1000, Promega, Anti-Active JNK (pTPpY) pAb Rabbit, no. 9255S], USP7 (1:200, cell signaling, HAUSP Rabbit Ab, no. 3277S), or beta-catenin (1:500, cell signaling, beta-catenin Rabbit Ab, no. 9562S) where indicated in blocking buffer with 0.1% (v/v) Tween-20 overnight at 4°C. The membranes were washed four times in PBS with 0.1% (v/v) Tween-20 for 5 min at room temperature and incubated with a 1:10,000 dilution of the Alexa Fluor 680 or 750 conjugated secondary antibodies (molecular probes).

### Quantitative real-time PCR analysis

Cells were reverse transfected in a six-well plate as described above. Following the incubation, total RNA was extracted from approximately 4 × 10^6^ cells using TRIzol reagent (Life Technologies). The culture media was aspirated from each well and 200 μl of TRIzol was added. Lysed cells were removed by scraping the wells with a cell lifter and RNA was extracted according to the manufacturer’s protocol. The RNA samples were purified of DNA by treatment with DNase I (Invitrogen). cDNA was generated from 5 μg of RNA using qScript cDNA SuperMix (Quanta Biosciences) according to the manufacturer’s protocol. A 1:16 dilution of the cDNA was employed for analysis. Transcript amplification was monitored with the Eppendorf realplex software, using KAPA SYBR FAST Universal 2× qPCR master mix (KAPA Biosystems) and the following primers: *USP7* forward 5′-AAA TGG TGT AAA TTT GAT GAC GAC G-3′, reverse 5′-CAA AAC TGG TCC TCT GCG ACT ATC-3′; *USP12* forward 5′-AAA TAA TAA CAG CAC ACC AGA CC-3′, reverse 5′-TTT GCT GCT TAT AGT TTC ACA AG-3′; *USP49* forward 5′-ACC AGT GTA ACA GCA AAC GAC-3′, reverse 5′-TCA TTA ACT GCT TTC TAG CTT CAC-3′; *USP54* forward 5′-ACC ACA GTG AAG GAG AAA CAC-3′, reverse 5′-CCA GGG ATG GTC TGT TAT G-3′; *FBXO36* forward 5′-CAC ATC TTC AAG GTC AAA CTG-3′, reverse 5′-GAT AGT CAG GAG CAA ATC GTC-3′; *KMT2D* forward 5′-GCC AAG CCT GCA GGA AAC-3′, reverse 5′-CAC GTC TCA CAA ACC AAC ATC-3′. Transcript expression levels for each experimental sample were normalized to actin expression levels (*actin* forward 5′-AAG ACC TGT ACG CCA ACA C-3′, reverse 5′-TCC ACA CGG AGT ACT TGC-3′) for that sample and to the non-targeting control sample using the ΔΔ*C_T_* method.

### TUNEL assay

Cells were reverse transfected as described above. Following the incubation, the culture medium was aspirated from each well and replaced with 50 μl of DMEM culture medium containing 10 ng/ml TNF and 10 μg/ml cycloheximide (Sigma) for 8 h at 37°C. The medium was aspirated and 100 μl 3.7% formaldehyde in PBS was added to each well. The plate was rocked manually 10–15 times and incubated at room temperature for at least 15 min without shaking. Formaldehyde was removed from the plate and the plate was washed with 100 μl 1×PBS for 6 min while shaking at room temperature, two times. Cells were permeabilized by adding fresh 0.1% sodium citrate/0.1% Triton X-100 in 1×PBS and incubating on ice for 2 min. The sodium citrate solution was removed from the plate. The plate was washed with 100 μl of 1×PBS for 6 min while shaking at room temperature, two times. The *in situ* cell death detection kit, TMR red (Roche), was employed to label apoptotic cells. Twenty microliters of fresh TUNEL reaction mixture (2 μl enzyme solution 1 and 18 μl label solution 2) was added to each well and samples were incubated at 37°C for 1 h. TUNEL reaction mixture was removed from the plate and the cells were washed with 100 μl 1×PBS for 6 min while shaking at room temperature, three times. DNA was labeled by incubating cells with 50 μl Hoechst 34328 stain (Life Technologies, 1:1000) in 1×PBS for at least 10 min and the plate was washed one more time in 100 μl 1×PBS. Cells were imaged using the Operetta High-Content Scanner (Perkin Elmer) and the number of TUNEL-positive cells in each well was quantified with Harmony software. The nuclei of individual cells were identified based on Hoechst staining of DNA. Cells on the edges of each image were excluded from subsequent analysis, so that only completely visible cells were quantified. The intensity of the TUNEL signal in the nucleus was calculated by the Harmony software. Nuclear TUNEL was defined as a TUNEL signal that overlapped with the Hoechst stain. Based on the negative and positive controls in each plate, an intensity threshold was defined for each plate that identified cells with predominant TUNEL signals. The intensity threshold was set to 450 mean pixel intensity. The remaining wells of the plate were then automatically visualized and quantified.

## Results

### A high-content assay for the nuclear localization of NF-κB

**Figure 1:**
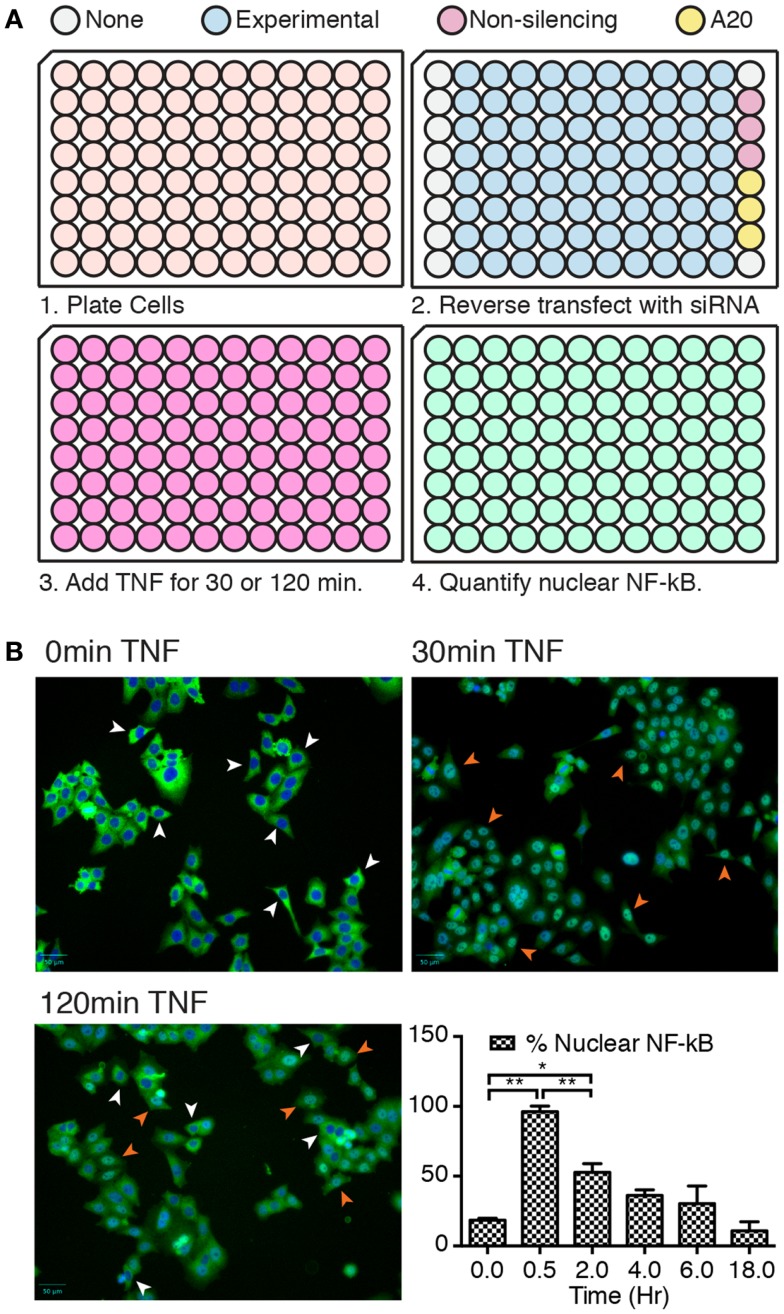
**(A)** Overview of key steps in the high-content assay. Step 2 shows the organization of siRNA pools within each plate. **(B)** Representative images of cells treated with TNF for 0, 30, and 120 min, and stained for NF-κB (green) and nuclear DNA (blue). Representative cells with cytosolic NF-κB are shown with white arrowheads and cells with nuclear NF-κB are shown with orange arrowheads. The bar chart shows the percentage of cells with nuclear NF-κB at the indicated times of TNF exposure. Results are shown as the average of three independent measurements and error bars indicate standard deviations. Statistical analysis was performed with a one-way ANOVA analysis, and one and two asterisks indicate *p* values below 0.05 and 0.01, respectively.

We developed a rapid and sensitive high-content assay to identify ubiquitin modifiers that regulate intracellular signals in response to TNF (Figure 1A). In this assay, we incubated HeLa cells in 96-well plates with pools of siRNA duplexes that target ubiquitin pathway elements. Eighty wells contained siRNA pools that targeted ubiquitin modifying enzymes, 10 wells did not contain any siRNA duplexes, 3 wells contained control non-silencing siRNAs, and 3 wells contained duplexes that targeted the ubiquitin-editing enzyme A20. Loss of A20 results in prolonged nuclear translocation of NF-κB in HeLa cells treated with TNF and serves as a reliable positive control for gene products that alter the activation of NF-κB.

We initially visualized and quantified the nuclear translocation of NF-κB at different times of TNF incubation (Figure 1B). NF-κB was predominantly cytosolic in cells that were not treated with TNF, translocated to the nucleus by 30 min TNF treatment, and gradually relocated to the cytosol with increasing periods of TNF exposure. From these results, we selected two time points to assay for modifiers of NF-κB localization. We tested the entire siRNA collection for duplexes that diminish the nuclear translocation of NF-κB at 30 min TNF incubation. We reasoned that such duplexes identify gene products that contribute to the nuclear localization of NF-κB. We also looked for siRNA duplexes that enhance the nuclear accumulation of NF-κB at 120 min TNF exposure. Such duplexes uncover candidate gene products that revert the subcellular localization of NF-κB at later times.

### Screen for ubiquitin modifiers that alter nuclear localization of NF-κB

**Figure 2:**
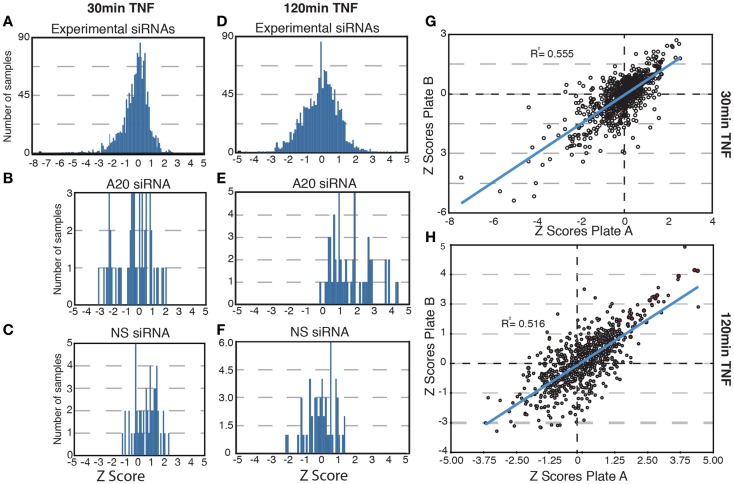
**Results of a high-content screen for ubiquitin modifiers of NF-κB**. **(A–E)** The distribution of *z*-scores are shown for all samples **(A,D)**; samples treated with control, non-silencing siRNAs (NS, C, and F); or samples treated with controls A20 siRNA **(B,E)**. **(A–C)** Show the *z*-score distributions for all samples at 30 min TNF and **(D–F)** show the *z*-score distribution for all samples at 120 min TNF. **(G–H)** Scatterplots show the replicate *z*-scores for the respective siRNAs at 30 min TNF **(G)** and 120 min TNF **(H)**. **(H)** Red dots show the *z*-scores for control A20 siRNAs and green dots show the *z*-scores for control non-silencing siRNAs.

In total, we tested a collection of 700 siRNA pools for effects on the nuclear translocation of NF-κB in response to TNF. We used standard score (*z*-score) analysis to determine the magnitude each siRNA pool alters the subcellular localization of NF-κB. The *z*-score is a measure of the extent to which an experimental group deviates from the median group within the same plate and is ideal for plate-to-plate comparisons. *z*-Scores above 1.96 or below −1.96 represent the fifth percentile groups of modifiers and are typically considered statistically significant. The results for each siRNA pool are presented in Tables S1–S4 in Supplementary Material. As expected, the bulk of experimental siRNAs had low *z*-scores at 30 and 120 min TNF exposure and do not appear to have a significant impact on the transduction of signals from TNF to NF-κB (Figures 2A,D). Likewise, control non-silencing siRNA duplexes did not modify the response of NF-κB to TNF at either time point (Figures 2C,F). The A20 siRNA duplexes did not prevent a nuclear accumulation of NF-κB at 30 min TNF incubation (Figure 2B), but frequently resulted in elevated *z*-scores suggest that prolonged nuclear accumulation of NF-κB at 120 min TNF exposure and are consistent with the physiological function of A20 (Figure 2E). Finally, comparison of the replicate assays indicated reproducible phenotypes for the majority of the siRNA duplexes tested at both time points (Figures 2G,H). In short, preliminary evaluation of the screen data suggests that the siRNA duplexes reproducibly generated predictable phenotypes that include a small number of significant modifiers of TNF-mediated nuclear accumulation of NF-κB.

### Ubiquitin modifiers of signals from TNF to NF-κB

The primary screen identified 12 putative modifiers of NF-κB localization at 30 min TNF treatment (Table 1) and an additional 9 putative modifiers at 120 min TNF exposure (Table 2).

**Table 1:** **Ubiquitin pathway modifiers of TNF signaling at 30 min**.

Gene ID	Name	Class	Phenotype	Lethal	Nuclear	Potential immune function (NCBI gene report)
NM_001014283	DCUN1D2	Neddylation	Reduced nuclear translocation	N	N	Neddylation of CUL1 (25)
NM_003592	CUL1	SCF E3 component	Reduced nuclear translocation	N	N	Ubiquitylation of I-κB (26)
NM_024907	FBXO17	SCF E3 component	Reduced nuclear translocation	Y	Y	None
NM_033645	FBXW11	SCF E3 component	Reduced nuclear translocation	N	N	Ubiquitylation of I-κB (27)
NM_003482	KMT2D	Lysine methyl transferase	Reduced nuclear translocation	N	N	No clear connection to ubiquitin pathway
NM_001024941	TRIM17	RING motif E3 ligase	Reduced nuclear translocation	N	N	None
NM_017999	RNF31	RING motif E3 ligase	Reduced nuclear translocation	N	N	AKA HOIP (28)
NM_005553	KRTAP5-9	Keratin protein	Reduced nuclear translocation	N	N	No clear connection to ubiquitin pathway
NM_005805	PSMD14	Deubiquitylation	Reduced nuclear translocation	Y	N	Proteasomal component
NM_182488	USP12	Deubiquitylation	Reduced nuclear translocation	N	N	*Drosophila* IMD response (29)
NM_031907	USP26	Deubiquitylation	Reduced nuclear translocation	N	N	None
NM_020718	USP31	Deubiquitylation	Reduced nuclear translocation	N	N	NF-κB activation (30)

**Table 2:** **Ubiquitin pathway modifiers of TNF signaling at 120 min**.

Gene ID	Gene name	Class	Phenotype	Lethal	Nuclear	Potential immune function (NCBI gene report)
NM_001032288	UBE2V1	E2	Prolonged nuclear translocation	N	N	NF-κB pathway regulation (31)
NM_012180	FBXO8	SCF E3 component	Prolonged nuclear translocation	N	N	None
NM_183413	FBXO44	SCF E3 component	Prolonged nuclear translocation	Y	N	None
NM_174899	FBXO36	SCF E3 component	Prolonged nuclear translocation	N	N	None
NM_006290	TNFA1P3	Ubiquitin-editing	Prolonged nuclear translocation	N	N	AKA A20. Termination of TNF signals (24)
NM_018561	USP49	Deubiquitylation	Prolonged nuclear translocation	N	N	None
NM_203494	USP50	Deubiquitylation	Prolonged nuclear translocation	N	N	Cell cycle
NM_152586	USP54	Deubiquitylation	Prolonged nuclear translocation	N	N	None
NM_003470	USP7	Deubiquitylation	Prolonged nuclear translocation	N	N	Activation of NF-κB (32)

**Figure 3:**
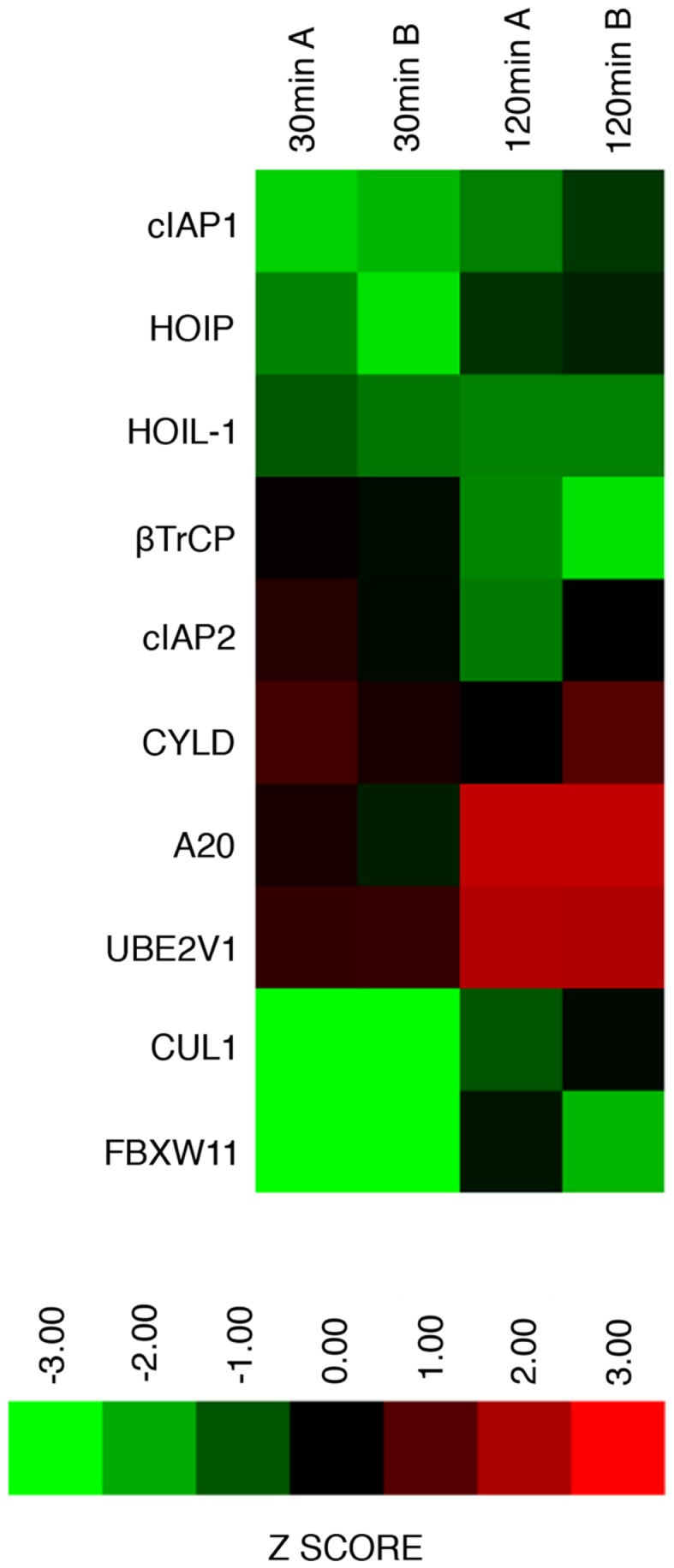
**Graphical representation of replicate assay *z*-scores assigned to known ubiquitin ligase modifiers of the TNF–NF-κB pathway in the primary siRNA screen**.

**Figure 4:**
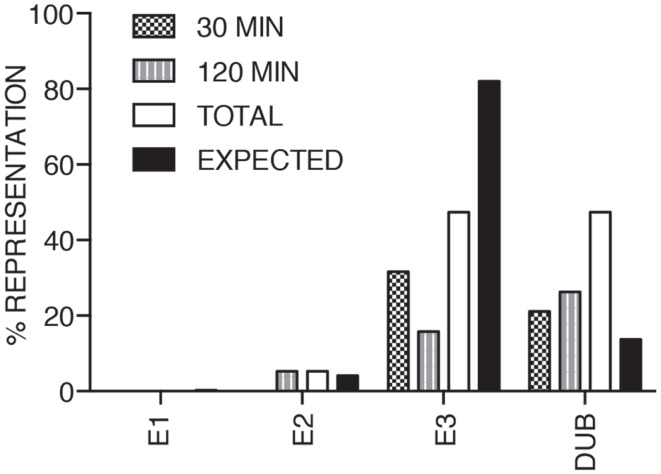
**The bar chart shows the relative percentages of E1, E2, and E3 ubiquitin ligases, and deubiquitylation enzymes identified as modifiers of the TNF pathway at 30, 120 min TNF exposure and in total**. The expected column shows the anticipated distribution assuming a normal representation of the respective functional groups.

We consider it noteworthy that several modifiers identified in our primary screen (e.g., CUL1, FBXW11, RNF31/HOIP, and TNFA1P3/A20) are known TNF pathway members. Furthermore, the phenotypes ascribed to the individual gene products overlap with the known molecular function of the target gene. Our ability to identify several known TNF pathway elements in an unbiased reverse genetics approach suggests an acceptable false-negative rate in the screen. Indeed, examination of the *z*-scores attributed to known TNF pathway members indicated that we consistently rated TNF pathway elements as substantial modifiers of NF-κB nuclear translocation (Figure 3). Of the 10 TNF pathway members tested, 5 (HOIP, A20, UBE2V1, CUL1, and FBXW11) were significant modifiers, and an additional 2 (c-IAP1 and HOIL-1) generated moderate phenotypes that matched their established biochemical functions. Two modifiers at 30 min (KRTAP5-9 and KMT2D) do not have obvious relationships to the ubiquitin pathway. We noticed an overrepresentation of deubiquitylation enzymes and underrepresentation of E3 ligases in the preliminary list of putative modifiers (Figure 4). The bias of hits toward deubiquitylation enzymes and away from E3 ligases may reflect differing functional redundancies within the respective groups.

### Phenotypic classes of ubiquitin pathway modifiers

**Figure 5:**
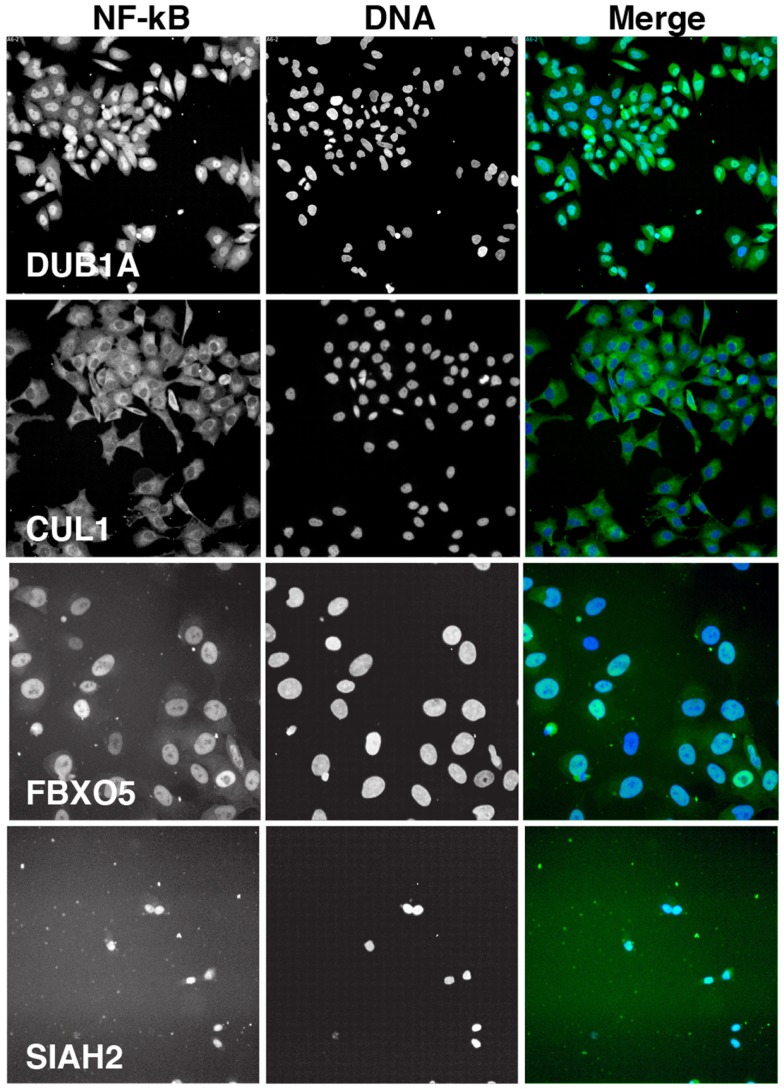
**Representative images of HeLa cells treated with the indicated siRNA pools and incubated with TNF for 30 min**. Each panel is shown at the same scale. NF-κB is shown in green in the merged image and DNA is shown in blue. CUL1 siRNA is a representative inhibitor of NF-κB nuclear accumulation, FBXO5 siRNA results in enlarged nuclei, and SIAH2 siRNA leads to extensive cell death.

We visually inspected the remaining putative modifiers to confirm the reported phenotypes. The results shown in Figure 5 are representative images of samples treated with a pool of siRNA duplexes and TNF for 30 min. As a reference, we included images of cells treated with DUB1A siRNA. DUB1A does not appear to play a role in the nuclear translocation of NF-κB and the bulk of HeLa cells have a visible nuclear accumulation of NF-κB. In contrast, cells treated with CUL1 siRNA have a pronounced absence of nuclear NF-κB at 30 min TNF treatment, consistent with the established requirement for CUL1 in the K48 ubiquitylation of I-κB. The majority of putative modifiers had visual phenotypes that agreed with the reported *z*-scores. However, we also detected two novel phenotypes within the list of putative modifiers. One siRNA pool (FBXO5) had greatly enlarged nuclei suggest that defects in cell division, and matches a known requirement for FBXO5 in progression through mitosis. An additional three pools resulted in widespread cell death. For example, treatment of HeLa cells with an siRNA pool that targets SIAH2 led to extensive loss of cells within 3 days. We excluded pools that led to pronounced cell death or nuclear enlargement from subsequent analysis.

### Validation of modifiers from the primary screen

**Figure 6:**
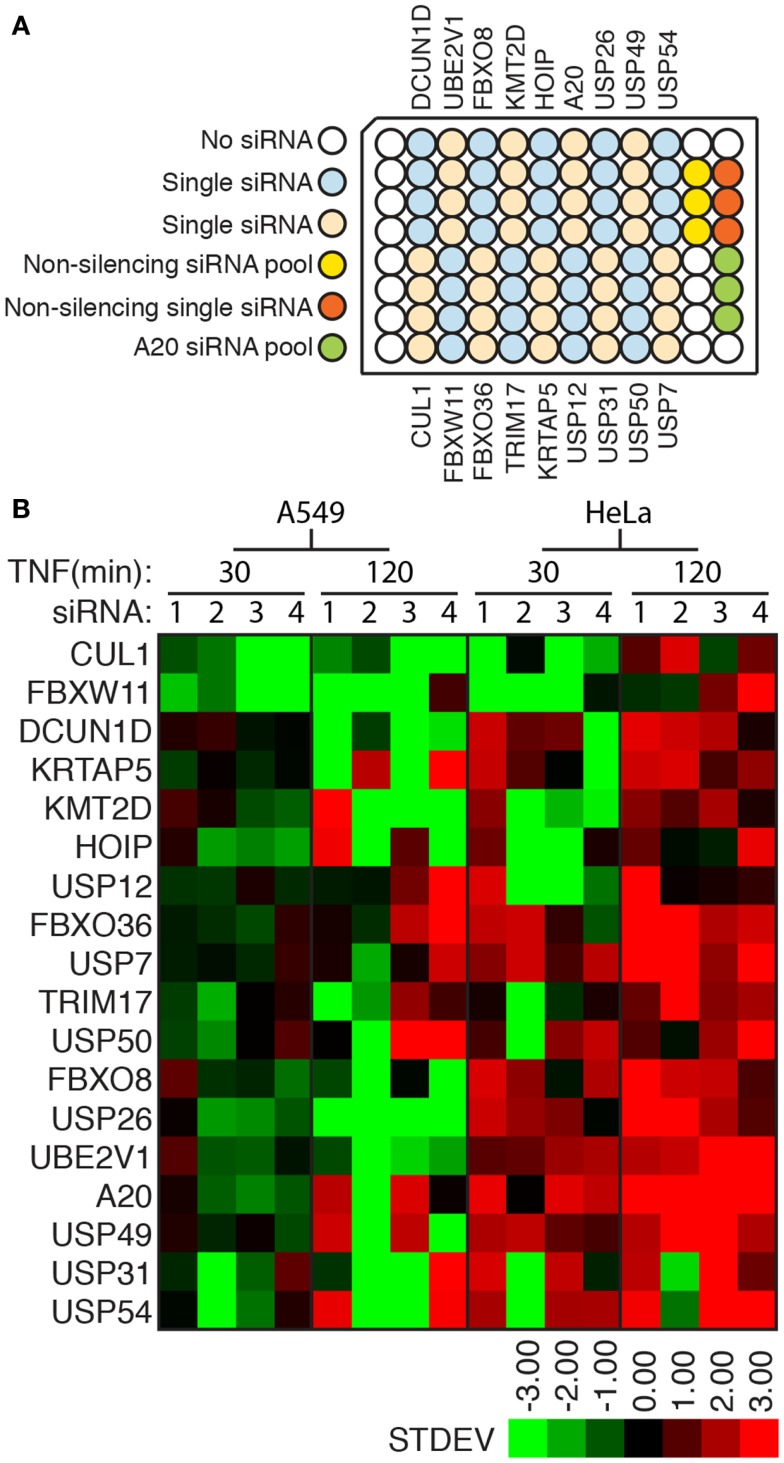
**(A)** Arrangement of experimental and control siRNA duplexes in a secondary assay for ubiquitin modifiers that control the nuclear translocation of NF-κB. **(B)** Graphical overview of the nuclear accumulation of NF-κB as a number of standard deviations from control non-silencing siRNA duplexes in HeLa or A549 cells treated with the respective siRNA duplexes and TNF for the indicated periods.

The phenotypes described in the primary screen were observed with pools of four siRNA duplexes that target non-overlapping sections of a mutual target. This approach permits an increased throughput in primary screens, but fails to adequately address concerns about potential off-target effects that result from the unintended depletion of non-targeted transcripts. To determine the extent to which off-target effects generated “hit” phenotypes in our primary screen, we performed a validation screen where we tested each individual siRNA alone for modification of NF-κB responses to TNF in HeLa cells and adenocarcinoma A549 cells. The organization of siRNA duplexes within the validation plate is shown in Figure 6A. In this assay, we defined an siRNA duplex as a modifier if it resulted in a nuclear accumulation of NF-κB that was greater than two standard deviations away from the median nuclear accumulation observed for cells treated with the control non-silencing siRNA (Figure 6B; Table S5 in Supplementary Material). For several targets, two or more individual duplexes generated phenotypes that matched our observations in the primary screen (e.g., FBXW11, HOIP, USP12, and A20; Figure 6B). In contrast, we found a number of cases where multiple individual duplexes failed to replicate the results of the primary screen (e.g., DCUN1D, USP54, TRIM17, and USP26). Our inability to replicate primary screen data with individual duplexes for several genes indicates that our initial screen generated several false positive hits. Statistical analysis of the primary data suggests that a false discovery rate of 0.501 in our screen.

Comparison between cell types uncovered a marked difference between A549 cells and HeLa cells. In particular, very few duplexes resulted in enhanced nuclear accumulation of NF-κB in A549 cells treated with TNF for prolonged periods. We interpret these data to suggest that A549 cells have a reduced ability to terminate TNF signals, as numerous TNF-responsive transcripts actively interrupt the nuclear accumulation of NF-κB and gene products such as CUL1, FBXW11, and HOIP are known targets of the TNF transcriptional machinery. Consistent with this hypothesis, we observed a substantially greater accumulation of NF-κB in HeLa cells depleted of A20 than in A549 cells.

We preliminarily defined a candidate as a potential hit if a minimum of two non-overlapping siRNAs yielded a modifier phenotype that mirrored the results observed in the primary screen. By these selection criteria, we identified four modifiers at 30 min TNF (CUL1, FBXW11, KMT2D, and USP12) treatment and an additional five modifiers at 120 min TNF (A20, USP7, USP49, USP54, and FBXO36) in HeLa cells. We observed identical 30 min TNF phenotypes for CUL1 and FBXW11 in A549 cells, a similar, although delayed phenotype for KMT2D, and a similar, but milder phenotype for USP12. As expected, the inhibitory phenotypes identified in HeLa cells were less pronounced upon inactivation of the same gene product in A549 cells. Nonetheless, a minimum of two siRNA molecules led to increased levels of nuclear NF-κB in A549 cells depleted of A20, USP7, USP49, USP54, or FBXO36 and treated with TNF for 120 min. Of these modifiers, three have clearly established roles in the regulation of TNF-dependent nuclear translocation of NF-κB (CUL1, FBXW11, and A20). In each case, the phenotypes described in the primary and validation screens overlap with the known molecular function of the respective gene products. In short, primary and secondary analysis indicates that the high-content approach detailed in this project detects a number of *bona fide* modifiers of the TNF–NF-κB axis, although orthogonal assays are required to identify false positives.

**Figure 7:**
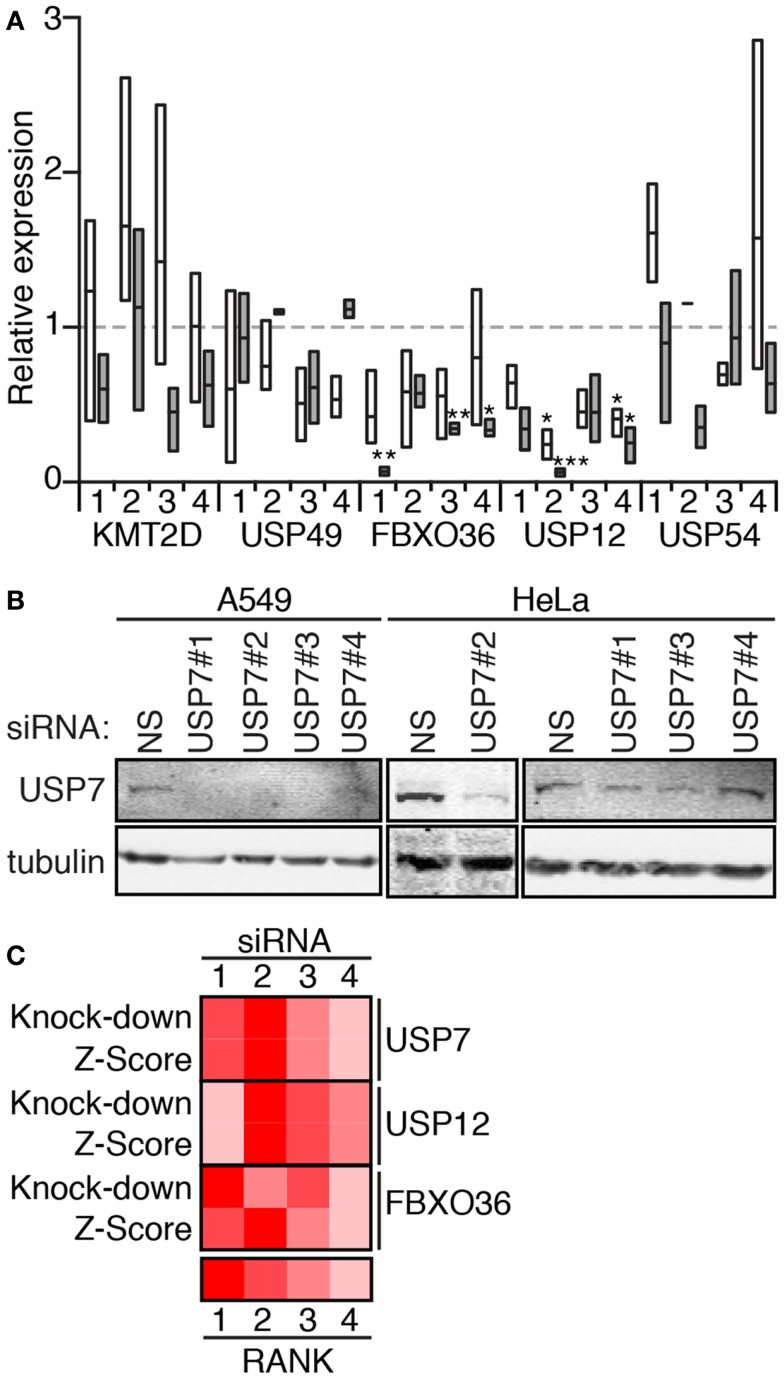
**(A)** Expression of the indicated genes in HeLa cells (open boxes) and A549 cells (gray boxes) treated with the corresponding siRNA (numbered 1–4). Box plots represent the three independent measurements and expression levels are reported relative to cells treated with a control non-silencing siRNA. Statistical evaluations were performed with a one-way ANOVA analysis comparing each sample mean to the non-silencing mean individually. One, two, and three asterisks indicate *p* values below 0.05, 0.01, and 0.001, respectively. **(B)** Western blot evaluation of lysates from A549 and HeLa cells treated with a control non-silencing siRNA (NS), or four independent siRNA duplexes that target USP7. Samples were probed with anti-USP7 antibodies and counter-probed with a tubulin antibody as a loading control. **(C)** Rank score evaluation of phenotypic severity for individual siRNAs that target USP12, USP7, and FBXO36 (*z*-score), and knock-down efficiency (knock-down for the corresponding siRNAs). *z*-Score data for USP12 are from the 30 min TNF exposure data presented in Figure 6, and *z*-score data from USP7 and FBXO36 are from the 120 min TNF exposure data presented in Figure 6.

We then used qPCR (Figure 7A) and Western blot analysis (Figure 7B) to determine the extent to which the individual siRNA duplexes affected expression of the target genes. As anticipated, the effects of the individual duplexes varied. In general, A549 cells appear more sensitive to the effects of RNAi. qPCR data suggest that RNAi-mediated inactivation of USP49, FBXO36, and USP12 depleted levels of the corresponding transcripts. In contrast, most KMT2D and USP54 duplexes had no appreciable effect on transcript levels. For several gene products (USP7, USP12, and FBXO36), we observed a correlation between the extent of target knock-down and the severity of the cellular phenotype (Figure 7C).

### Interactions of putative ubiquitin pathway modifiers of NF-κB with I-κB

**Figure 8:**
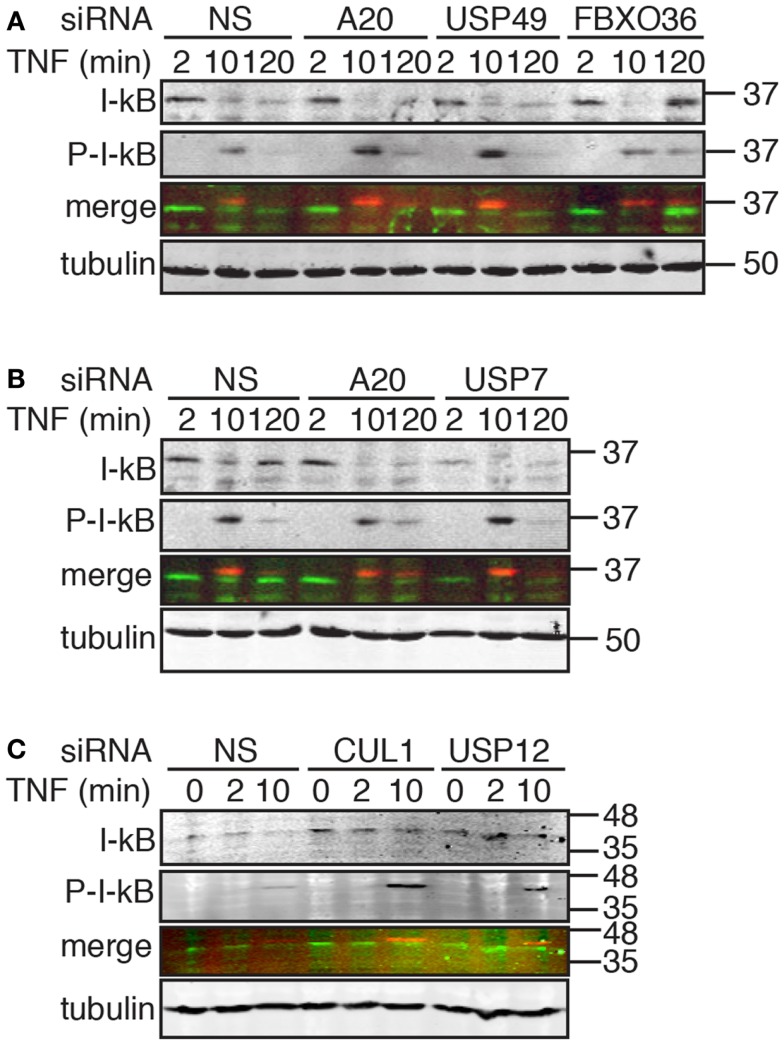
**Western blot evaluation of total I-κB and phosphorylated I-κB (P-I-κB) in cells treated with the indicated siRNAs and incubated with TNF for the indicated times**. In the merged images, I-κB is shown in green and P-I-κB is shown in red. Tubulin is shown as a loading control and numbers on the side indicate molecular weights in kilodaltons.

**Figure 9:**
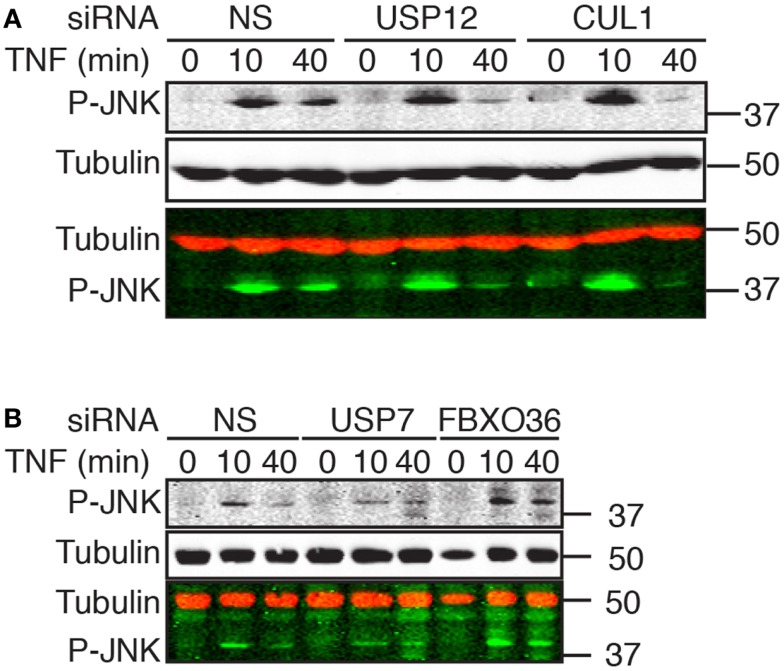
**Western blot evaluation of total phosphorylated JNK (P-JNK) in cells treated with the indicated siRNAs and incubated with TNF for the indicated times**. In the merged images, P-JNK is shown in green and tubulin is shown in red. Molecular weights are shown in kilodaltons.

**Figure 10:**
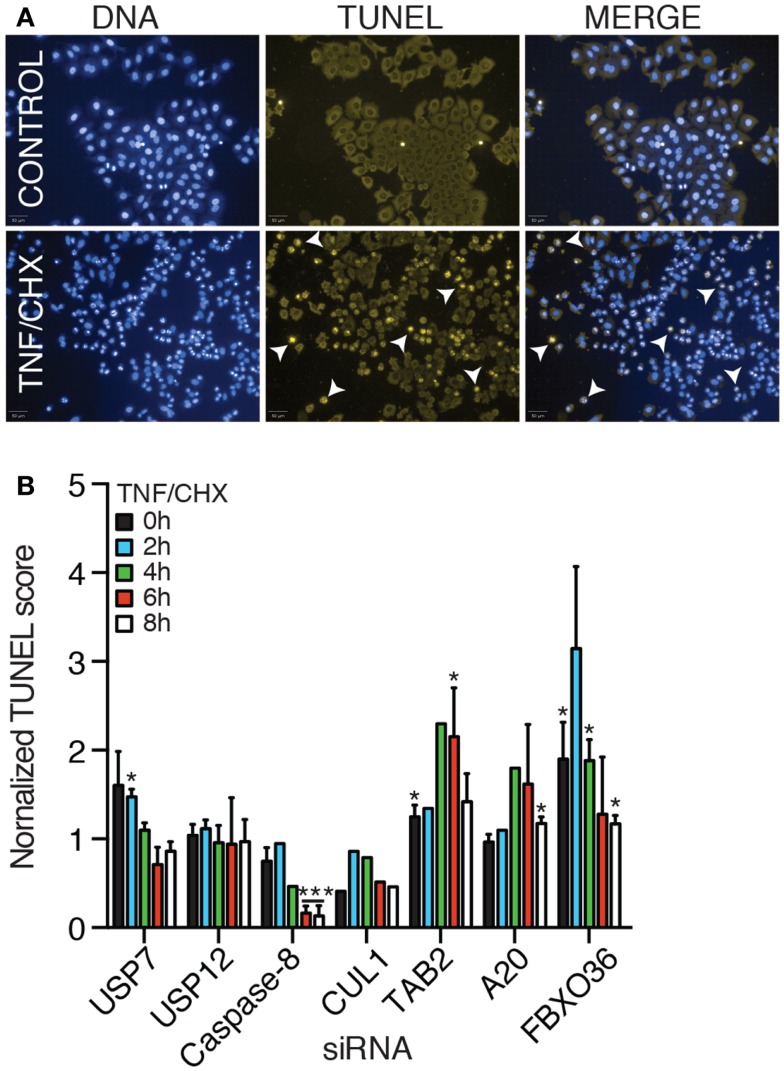
**(A)** TUNEL and DNA stain of HeLa cells (upper row), or HeLa cells treated with TNF and cycloheximide (CHX) for 8 h (lower row). **(B)** Relative levels of apoptosis in HeLa cells treated with the indicated siRNA duplexes and TNF and cycloheximide for the indicated periods. For each time point, the level of apoptosis in cells treated with non-silencing (NS) siRNA were assigned a value of 1 and other treatments are reported relative to the control. **(B)** Results are the mean of three separate experiments and error bars indicate the standard deviation. Statistical analysis was performed with a one-way ANOVA analysis, and one and three asterisks indicate *p* values below 0.05 and 0.001, respectively.

**Figure 11:**
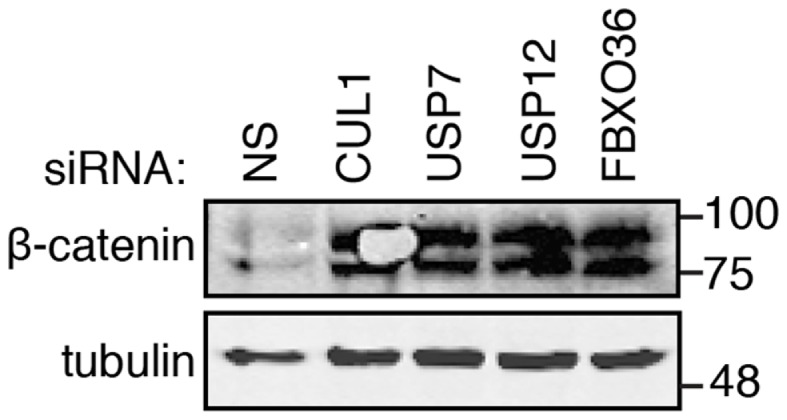
**Western blot evaluation of beta-catenin (β-catenin) in cells treated with the indicated siRNAs**. Tubulin is shown as a loading control. Molecular weights are shown in kilodaltons.

Based on the validation data presented in Figures 6 and 7, we performed a series of orthogonal assays to probe the involvement of USP7, USP12, USP49, and FBXO36 in TNF pathway activity. We do not consider USP54 and KMT2D suitable candidates for study, given the lack of target knock-down by the corresponding siRNA duplexes. For all orthogonal assays (Figures 8–11), we used single siRNA duplexes to target each gene. Specifically, we used duplex no. 2 to target USP7; duplex no. 2 to target USP12; duplex no. 3 to target USP49; and duplex no. 3 to target FBXO36.

Signals from TNF lead to the phosphorylation, ubiquitylation, and destruction of I-κB with a concomitant nuclear translocation of NF-κB. A subset of NF-κB-responsive transcripts, such as I-κB and A20, terminate the signal through the sequestration of NF-κB and the editing of ubiquitylated TNF pathway components, respectively. The causal relationship between phosphorylation-dependent ubiquitylation of I-κB and nuclear translocation of NF-κB led us to explore the impact of hits from the secondary screen on I-κB. For these assays, we treated cells with siRNA duplexes that target putative modifiers of NF-κB nuclear accumulation at 30 min TNF (CUL1 and USP12) and at 120 min TNF (A20, USP7, USP49, and FBXO36). For the former group of siRNAs, we examined the extent of I-κB phosphorylation and destruction at early times after TNF exposure. For the latter group of siRNAs, we examined the early turnover of I-κB 10 min after TNF exposure and the subsequent re-accumulation of I-κB 120 min after TNF exposure.

As expected, we detected a rapid phosphorylation of I-κB and a decrease in total I-κB levels by 10 min, and a re-accumulation of I-κB by 120 min in cells treated with TNF and a control non-silencing siRNA (Figure 8A). Loss of USP49 or FBXO36 did not have noticeable effects on the dynamics of I-κB stability (Figure 8A). However, we noticed a persistent late stage phosphorylation of I-κB in cells treated with FBXO36 siRNA that was absent from cells treated with a control siRNA. As expected for a gene product that terminates TNF signals to NF-κB, loss of A20 prevents re-accumulation of I-κB 120 min after incubation with TNF (Figure 8B). Loss of USP7 diminished I-κB at all time points tested (Figure 8B), indicating a relationship between USP7 activity and I-κB protein levels. Depletion of CUL1 or USP12 resulted in a pronounced accumulation of phosphorylated I-κB at 10 min TNF incubation (Figure 8C). These observations suggest that CUL1 and USP12 are involved in the proteasomal turnover of phosphorylated I-κB. As USP49 duplexes had minimal effects on USP49 expression, or I-κB protein turnover, we excluded USP49 from further analysis. We selected USP7, USP12, and FBXO36 as promising leads for the identification of novel ubiquitin pathway elements that determine the outcomes of TNF signals.

### Interactions of putative ubiquitin ligase modifiers of NF-κB with JNK

As the TAB2–TAK1 module drives the transient phosphorylation of JNK by MAPKK4 and MAPKK7 in response to TNF, we examined the relationship between ubiquitin pathway components and the transduction of signals from TNF to JNK. In cells treated with a non-silencing siRNA, exposure to TNF resulted in robust JNK phosphorylation by 10 min with a rapid decline in phospho-JNK levels over time. The phenotype of cells treated with USP12 or CUL1 siRNA deviated mildly from cells treated with control siRNA; we detected a minor increase in the early TNF-mediated phosphorylation of JNK and a faster decline in phospho-JNK levels (Figure 9A). In cells treated with FBXO36 siRNA, we detected a moderate, but reproducible prolonged phosphorylation of JNK, while loss of USP7 results in a decreased phosphorylation of JNK (Figure 9B). Combined, our data elaborate the phenotypic contributions of novel ubiquitin pathway members to the TNF signal transduction pathway. Loss of USP12 results in accumulation of phosphorylated I-κB, diminished nuclear accumulation of NF-κB, and modified activation of JNK. In contrast, loss of USP7 results in decreased expression of I-κB, decreased activation of JNK, and prolonged nuclear accumulation of NF-κB. Depletion of FBXO36 appears to result in enhanced signal transduction through the TNF pathway kinase relay, with prolonged phosphorylation of JNK and I-κB.

### Interactions of putative ubiquitin ligase modifiers of NF-κB with apoptosis

In the absence of signal transduction from TNF to NF-κB, engagement of the TNFR typically results in the activation of programed cell death. We developed a high-content assay to quantify apoptotic cell death in HeLa cells. For this assay, we incubated HeLa cells with TNF and prevented the expression of NF-κB-responsive anti-apoptotic factors through the addition of the translation inhibitor cycloheximide. As a consequence, signals from the TNFR induce mitochondrial permeabilization and the induction of cell death. We used a TUNEL stain to visualize the fragmentation of nuclear DNA that accompanies apoptotic death. Despite variability in the extent of cell death in individual replicates, we consistently detected that addition of TNF and cycloheximide to HeLa cells resulted in a gradual increase in the levels of apoptosis (Figures 10A,B). As expected, loss of the pro-apoptotic effector caspase-8 prevented the induction of cell death and loss of the TNF pro-survival pathway element TAB2 enhanced cell death (Figure 10B). Thus, the assay presents a useful forum to interrogate the contribution of gene products to apoptosis. Depletion of USP7 and USP12 did not substantially modify the induction of cell death by a TNF–cycloheximide regime. In contrast, loss of A20 and FBXO36 enhanced the induction of cell death by TNF and cycloheximide, particularly at early time points. These observations overlap with an established role for A20 in the prevention of TNF-dependent death and hint at a similar function for FBXO36.

### An orthologous assay for putative ubiquitin pathway modifiers of the TNF response

The primary and secondary data presented in this report support roles for USP7, USP12, and FBXO36 in the control of the TNF pathway. In our original assay, we explored the regulation of NF-κB nuclear translocation, a process that requires I-κB ubiquitylation by the SCF–bTrCP E3 ligase. To explore relationships between candidate ubiquitin pathway members and the SCF–bTrCP complex, we examined beta-catenin levels in cells depleted of USP7, USP12, and FBXO36, respectively (Figure 11). Beta-catenin is a target for ubiquitylation by SCF–bTrCP, and we hypothesized that modifiers of I-κB ubiquitylation will alter steady state levels of beta-catenin. Consistent with this hypothesis, loss of CUL1 resulted in increased levels of beta-catenin. Likewise, depletion of USP12, USP7, or FBXO36 increased total cellular levels of beta-catenin. These results support a general role for USP7, USP12, and FBXO36 as putative modifiers of the SCF–bTrCP E3 ligase.

## Discussion

Ubiquitin adds a sophisticated and frequently underappreciated degree of complexity to cellular events. Ubiquitin conjugates determine the half-life, localization, and function of substrate proteins and regulate the assembly of macromolecular complexes into functional groups. Remarkably, we know very little about the coordination of ubiquitin and TNF pathway responses during inflammation. For example, the ubiquitylated complex I element that recruits TAB2–TAK1 is unknown, and the deubiquitylation enzymes that remove linear and K63-linked ubiquitin chains from complex I components require characterization. To address this issue, we conducted a ubiquitin pathway siRNA screen for enzymes that regulate TNF-mediated nuclear translocation of NF-κB. We confirmed established TNF signaling modifiers and identified several novel candidate components. We believe that the candidate proteins represent potentially valuable targets for therapeutic regulation of TNF-mediated inflammation.

Off-target effects that result from the unintended inactivation of a non-targeted transcript are a frequent source of concern in RNAi screens (33). In this report, we describe an unbiased RNAi platform and a series of orthogonal cell culture based studies that position a cohort of novel gene products within the TNF pathway. We believe that this cohort is a valuable set of targets for evaluation in animal models. However, additional studies are required to substantiate roles for the individual gene products in the TNF to NF-κB axis. While there is a body of literature to support a role for USP7 in the TNF pathway, the involvement of USP12 and FBXO36 required additional validation experiments. It is common practice for investigators to validate a phenotype with additional dsRNAs, molecular, biochemical, and cell biological studies, and where possible an examination of an animal model where the target gene is inactivated through conventional mutagenesis techniques. We believe secondary assays that probe the effects of USP12 and FBXO36 on transcriptional responses to TNF are good candidates for follow-up experiments.

We consider false negatives an equally important and often neglected source of concern in RNAi screens. False negatives arise when a relevant gene product is not sufficiently depleted in a particular screen or when the screen itself is not robust enough to uncover meaningful hits. A recent bioinformatic study indicated that false negatives are a common source of error in RNAi screens (34). We note that false negatives are clearly an issue in our screen, as our secondary selection criteria excluded the known LUBAC component HOIP from a more detailed analysis. Investigators can mitigate the impact of false negatives to some extent by optimizing the sensitivity of their screen and by testing several dsRNA molecules for each target, but we consider it likely that current screening technologies have an appreciable frequency of false negatives.

With these considerations in mind, we recommend investigators take a parsimonious approach in the evaluation of primary data from large-scale RNAi screens, including the one presented here. The entire data set of our primary screen is available in Tables S1–S4 in Supplementary Material. Apparent phenotypes should not be considered truly biologically relevant without in-depth follow-up studies, and absence of a phenotype should not be considered absence of a function. We are satisfied that USP7, USP12, and FBXO36 are reasonable candidates for consideration as TNF modifiers, as we detected reproducible phenotypes in duplicate assays with siRNA pools and individual duplexes, and generated clear phenotypes in secondary assays that interrogated specific aspects of TNF signaling. On a cautionary note, our studies were limited to direct examination of NF-κB localization and did not examine downstream transcriptional events.

*In vitro* studies indicate interactions between USP7 and TNF pathway members TRAF2 and NEDD4 (35). The USP7 phenotype described in this report is quite complex, as depletion of USP7 led to a loss of I-κB protein and a stabilization of beta-catenin. The constitutive loss of I-κB protein observed upon depletion of USP7 may reflect a non-proteasomal requirement for USP7 in transcriptional processes. Recent publications identified functional relationships between USP7 and the regulation of apoptosis or NF-κB transcriptional activity in the TNF pathway (32, 36). These parallel observations support our positioning of USP7 within the TNF–NF-κB axis. There are no available data on the role of USP12 in the control of TNF signaling. However, a previous report from our group identified the *Drosophila* ortholog of USP12 as a modifier of the immune deficiency (IMD) pathway (29). We consider these findings striking, as the IMD and TNF pathways share an evolutionary conserved core of molecules that regulate JNK, NF-κB, and caspase responses. To date, FBXO36 has not been described in the literature. However, F box proteins interact with CUL1 and our data consistently suggested that loss of FBXO36 results in heightened signal transduction through the TNF pathway. The simplest explanation for this phenotype is that disruption of interactions between CUL1 and FBXO36 alter the activity of SCF–bTrCP.

Biochemical and mutational studies are required to fully establish the roles of FBXO36, USP7, and USP12 in the TNF pathway. However, a preliminary organization of the phenotypes identified for the respective gene products in various secondary assays hint at functional relationships. For example, USP12 and CUL1 RNAi phenotypes are strikingly similar in a number of secondary assays, suggesting similar requirements for both gene products in the activation of the SCF–bTrCP complex. FBXO36 has a unique phenotype that deviates considerably from A20 and CUL1, indicating a distinct relationship between FBXO36 and the TNF pathway. The non-overlapping nature of the FBXO36 and USP12 phenotypes are fortuitous, as they represent unique pharmacological targets for the manipulation of TNF pathway activity and intensity.

## Author Contributions

Conceived and designed the experiments: Edan Foley, Brittany Fraser, and Robert Maranchuk. Performed the RNAi screen: Brittany Fraser and Robert Maranchuk. Performed the secondary analysis: Brittany Fraser. Performed the analysis: Brittany Fraser and Edan Foley. Drafted and reviewed the manuscript: Brittany Fraser and Edan Foley.

## Conflict of Interest Statement

The authors declare that the research was conducted in the absence of any commercial or financial relationships that could be construed as a potential conflict of interest.

## Supplementary Material

The Supplementary Material for this article can be found online at http://www.frontiersin.org/Journal/10.3389/fimmu.2014.00322/abstract

## References

[1] Chen G Goeddel DV TNF-R1 signaling: a beautiful pathway. Science (2002) 296:1634–5 10.1126/science.1071924 12040173

[2] Aggarwal BB Signalling pathways of the TNF superfamily: a double-edged sword. Nat Rev Immunol (2003) 3:745–56 10.1038/nri1184 12949498

[3] Medzhitov R Inflammation 2010: new adventures of an old flame. Cell (2010) 140:771–6 10.1016/j.cell.2010.03.006 20303867

[4] Nathan C Ding A Nonresolving inflammation. Cell (2010) 140:871–82 10.1016/j.cell.2010.02.029 20303877

[5] Grivennikov SI Greten FR Karin M Immunity, inflammation, and cancer. Cell (2010) 140:883–99 10.1016/j.cell.2010.01.025 20303878 PMC2866629

[6] Kriegler M Perez C Defay K Albert I Lu SD A novel form of TNF/cachectin is a cell surface cytotoxic transmembrane protein: ramifications for the complex physiology of TNF. Cell (1988) 53:45–53 10.1016/0092-8674(88)90486-2 3349526

[7] Jiang Y Woronicz JD Liu W Goeddel DV Prevention of constitutive TNF receptor 1 signaling by silencer of death domains. Science (1999) 283:543–6 10.1126/science.283.5401.543 9915703

[8] Hsu H Xiong J Goeddel DV The TNF receptor 1-associated protein TRADD signals cell death and NF-kappa B activation. Cell (1995) 81:495–504 10.1016/0092-8674(95)90070-5 7758105

[9] Micheau O Tschopp J Induction of TNF receptor I-mediated apoptosis via two sequential signaling complexes. Cell (2003) 114:181–90 10.1016/S0092-8674(03)00521-X 12887920

[10] Hsu H Huang J Shu HB Baichwal V Goeddel DV TNF-dependent recruitment of the protein kinase RIP to the TNF receptor-1 signaling complex. Immunity (1996) 4:387–96 10.1016/S1074-7613(00)80252-6 8612133

[11] Tada K Okazaki T Sakon S Kobarai T Kurosawa K Yamaoka S Critical roles of TRAF2 and TRAF5 in tumor necrosis factor-induced NF-kappa B activation and protection from cell death. J Biol Chem (2001) 276:36530–4 10.1074/jbc.M104837200 11479302

[12] Varfolomeev E Goncharov T Fedorova AV Dynek JN Zobel K Deshayes K c-IAP1 and c-IAP2 are critical mediators of tumor necrosis factor alpha (TNFalpha)-induced NF-kappaB activation. J Biol Chem (2008) 283:24295–9 10.1074/jbc.C800128200 18621737 PMC3259840

[13] Fang S Weissman AM A field guide to ubiquitylation. Cell Mol Life Sci (2004) 61:1546–61 10.1007/s00018-004-4129-5 15224180 PMC11138666

[14] Walczak H TNF and ubiquitin at the crossroads of gene activation, cell death, inflammation, and cancer. Immunol Rev (2011) 244:9–28 10.1111/j.1600-065X.2011.01066.x 22017428

[15] Kanayama A Seth RB Sun L Ea CK Hong M Shaito A TAB2 and TAB3 activate the NF-kappaB pathway through binding to polyubiquitin chains. Mol Cell (2004) 15:535–48 10.1016/j.molcel.2004.08.008 15327770

[16] Ea CK Deng L Xia ZP Pineda G Chen ZJ Activation of IKK by TNFalpha requires site-specific ubiquitination of RIP1 and polyubiquitin binding by NEMO. Mol Cell (2006) 22:245–57 10.1016/j.molcel.2006.03.026 16603398

[17] Li H Kobayashi M Blonska M You Y Lin X Ubiquitination of RIP is required for tumor necrosis factor alpha-induced NF-kappaB activation. J Biol Chem (2006) 281:13636–43 10.1074/jbc.M600620200 16543241

[18] Wu CJ Conze DB Li T Srinivasula SM Ashwell JD Sensing of Lys 63-linked polyubiquitination by NEMO is a key event in NF-kappaB activation [corrected]. Nat Cell Biol (2006) 8:398–406 10.1038/ncb1384 16547522

[19] Wajant H Scheurich P TNFR1-induced activation of the classical NF-kappaB pathway. FEBS J (2011) 278:862–76 10.1111/j.1742-4658.2011.08015.x 21232017

[20] Tokunaga F Iwai K LUBAC, a novel ubiquitin ligase for linear ubiquitination, is crucial for inflammation and immune responses. Microbes Infect (2012) 14:563–72 10.1016/j.micinf.2012.01.011 22309894

[21] van Raam BJ Salvesen GS Proliferative versus apoptotic functions of caspase-8 hetero or homo: the caspase-8 dimer controls cell fate. Biochim Biophys Acta (2012) 1824:113–22 10.1016/j.bbapap.2011.06.005 21704196 PMC3993904

[22] Micheau O Lens S Gaide O Alevizopoulos K Tschopp J NF-kappaB signals induce the expression of c-FLIP. Mol Cell Biol (2001) 21:5299–305 10.1128/MCB.21.16.5299-5305.2001 11463813 PMC87253

[23] Song HY Rothe M Goeddel DV The tumor necrosis factor-inducible zinc finger protein A20 interacts with TRAF1/TRAF2 and inhibits NF-kappaB activation. Proc Natl Acad Sci U S A (1996) 93:6721–5 10.1073/pnas.93.13.6721 8692885 PMC39093

[24] Jaattela M Mouritzen H Elling F Bastholm L A20 zinc finger protein inhibits TNF and IL-1 signaling. J Immunol (1996) 156:1166–73 8557994

[25] Kurz T Ozlu N Rudolf F O’Rourke SM Luke B Hofmann K The conserved protein DCN-1/Dcn1p is required for cullin neddylation in *C. elegans* and *S. cerevisiae*. Nature (2005) 435:1257–61 10.1038/nature03662 15988528

[26] Winston JT Strack P Beer-Romero P Chu CY Elledge SJ Harper JW The SCFbeta-TRCP-ubiquitin ligase complex associates specifically with phosphorylated destruction motifs in IkappaBalpha and beta-catenin and stimulates IkappaBalpha ubiquitination in vitro. Genes Dev (1999) 13:270–83 10.1101/gad.13.3.270 9990852 PMC316433

[27] Suzuki H Chiba T Suzuki T Fujita T Ikenoue T Omata M Homodimer of two F-box proteins betaTrCP1 or betaTrCP2 binds to IkappaBalpha for signal-dependent ubiquitination. J Biol Chem (2000) 275:2877–84 10.1074/jbc.275.4.2877 10644755

[28] Haas TL Emmerich CH Gerlach B Schmukle AC Cordier SM Rieser E Recruitment of the linear ubiquitin chain assembly complex stabilizes the TNF-R1 signaling complex and is required for TNF-mediated gene induction. Mol Cell (2009) 36:831–44 10.1016/j.molcel.2009.10.013 20005846

[29] Bond D Foley E A quantitative RNAi screen for JNK modifiers identifies PVR as a novel regulator of *Drosophila* immune signaling. PLoS Pathog (2009) 5:e1000655 10.1371/journal.ppat.1000655 19893628 PMC2766254

[30] Tzimas C Michailidou G Arsenakis M Kieff E Mosialos G Hatzivassiliou EG Human ubiquitin specific protease 31 is a deubiquitinating enzyme implicated in activation of nuclear factor-kappaB. Cell Signal (2006) 18:83–92 10.1016/j.cellsig.2005.03.017 16214042

[31] Deng L Wang C Spencer E Yang L Braun A You J Activation of the IkappaB kinase complex by TRAF6 requires a dimeric ubiquitin-conjugating enzyme complex and a unique polyubiquitin chain. Cell (2000) 103:351–61 10.1016/S0092-8674(00)00126-4 11057907

[32] Colleran A Collins PE O’Carroll C Ahmed A Mao X Mcmanus B Deubiquitination of NF-kappaB by ubiquitin-specific protease-7 promotes transcription. Proc Natl Acad Sci U S A (2013) 110:618–23 10.1073/pnas.1208446110 23267096 PMC3545798

[33] Echeverri CJ Beachy PA Baum B Boutros M Buchholz F Chanda SK Minimizing the risk of reporting false positives in large-scale RNAi screens. Nat Methods (2006) 3:777–9 10.1038/nmeth1006-777 16990807

[34] Hao L He Q Wang Z Craven M Newton MA Ahlquist P Limited agreement of independent RNAi screens for virus-required host genes owes more to false-negative than false-positive factors. PLoS Comput Biol (2013) 9:e1003235 10.1371/journal.pcbi.1003235 24068911 PMC3777922

[35] Zapata JM Pawlowski K Haas E Ware CF Godzik A Reed JC A diverse family of proteins containing tumor necrosis factor receptor-associated factor domains. J Biol Chem (2001) 276:24242–52 10.1074/jbc.M100354200 11279055

[36] Zaman MM Nomura T Takagi T Okamura T Jin W Shinagawa T Ubiquitination-deubiquitination by the TRIM27-USP7 complex regulates tumor necrosis factor alpha-induced apoptosis. Mol Cell Biol (2013) 33:4971–84 10.1128/MCB.00465-13 24144979 PMC3889550

