# Supplementary material for: A High-Content RNAi Screen Identifies Ubiquitin Modifiers That Regulate TNF-Dependent Nuclear Accumulation of NF-κB
Source: Front Immunol. 2014 Jul 14;5:322. doi: 10.3389/fimmu.2014.00322 (PMC4094887; doi:10.3389/fimmu.2014.00322)
Supplement: Supplementary file 1 [file Presentation_1.ZIP › Supp. Table 3.PDF]

	Gene ID	Gene	30 mins # of cells	120 mins # of cells	Lethal	Nuclear phenotype	30 mins % NF-kB Translocation	30 mins Z-score	120 mins % NF-kB Translocation	120 mins Z-score
Plate A	NM_006879	MDM2	126	1113	N	N	70.63	-0.76	85.44	1.75
Plate B			576	1014			90.10	0.46	75.25	1.67
Avg			351	1063.5			80.37	-0.15	80.35	1.71
Plate A	NM_016483	PHF7	579	503	N	N	90.67	0.46	41.15	-0.14
Plate B			758	590			79.42	-0.11	35.93	0.01
Avg			668.5	546.5			85.05	0.17	38.54	-0.07
Plate A	NM_172016	TRIM39	516	762	N	N	92.25	0.56	53.54	0.39
Plate B			546	737			72.34	-0.50	25.78	-0.42
Avg			531	749.5			82.30	0.03	39.66	-0.01
Plate A	NM_201627	TRIM41	625	703	N	N	96.16	0.79	61.31	0.72
Plate B			354	428			85.03	0.19	30.14	-0.23
Avg			489.5	565.5			90.59	0.49	45.72	0.24
Plate A	NM_139175	RNF133	971	1321	N	N	85.89	0.17	57.23	0.54
Plate B			830	1129			77.71	-0.21	50.22	0.62
Avg			900.5	1225			81.80	-0.02	53.73	0.58
Plate A	NM_030647	KIAA1718	396	1112	N	N	91.41	0.51	76.80	1.38
Plate B			894	632			95.75	0.77	55.06	0.82
Avg			645	872			93.58	0.64	65.93	1.10
Plate A	NM_030936	RNF32	226	253	N	N	76.99	-0.37	22.13	-0.96
Plate B			273	269			86.08	0.25	27.88	-0.33
Avg			249.5	261			81.54	-0.06	25.01	-0.64
Plate A	NM_020395	INTS12	776	968	N	N	77.32	-0.35	13.33	-1.33
Plate B			884	905			74.21	-0.40	17.79	-0.75
Avg			830	936.5			75.76	-0.37	15.56	-1.04
Plate A	NM_014630	ZNF592	608	383	N	N	85.20	0.13	18.54	-1.11
Plate B			149	110			82.55	0.05	3.64	-1.35
Avg			378.5	246.5			83.87	0.09	11.09	-1.23
Plate A	NM_001093725	LOC92312	644	957	N	N	73.76	-0.57	18.50	-1.11
Plate B			721	753			58.95	-1.22	5.84	-1.26
Avg			682.5	855			66.35	-0.89	12.17	-1.19
Plate A	NM_014502	PRPF19	243	645	N	N	75.72	-0.45	42.64	-0.08
Plate B			930	518			91.94	0.56	46.53	0.46
Avg			586.5	581.5			83.83	0.06	44.58	0.19
Plate A	NM_014248	RBX1	427	711	N	N	67.45	-0.95	77.78	1.42
Plate B			534	608			60.67	-1.13	54.77	0.81
Avg			480.5	659.5			64.06	-1.04	66.27	1.12
Plate A	XM_926073	LOC653111	330	740	N	N	44.24	-2.36	61.49	0.73
Plate B			464	973			37.28	-2.39	44.60	0.38
Avg			397	856.5			40.76	-2.38	53.05	0.55
Plate A	NM_002152	HRC	774	738	N	N	94.44	0.69	71.27	1.14
Plate B			367	482			84.20	0.14	48.13	0.53
Avg			570.5	610			89.32	0.42	59.70	0.84
Plate A	NM_015057	MYCBP2	472	741	N	N	83.26	0.01	31.17	-0.57
Plate B			498	468			76.71	-0.26	8.12	-1.16
Avg			485	604.5			79.98	-0.13	19.65	-0.87
Plate A	NM_153026	PRICKLE1	340	628	N	N	71.76	-0.69	21.97	-0.96
Plate B			195	548			53.85	-1.50	21.35	-0.60
Avg			267.5	588			62.81	-1.09	21.66	-0.78
Plate A	NM_005188	CBL	360	712	N	N	90.83	0.47	51.26	0.29
Plate B			559	249			83.36	0.10	8.03	-1.17
Avg			459.5	480.5			87.10	0.28	29.65	-0.44
Plate A	NM_015557	CHD5	369	598	N	N	53.12	-1.82	16.56	-1.20
Plate B			745	427			62.42	-1.03	6.79	-1.22
Avg			557	512.5			57.77	-1.43	11.67	-1.21
Plate A	NM_178532	RNF180	333	324	N	N	52.25	-1.87	2.78	-1.79
Plate B			322	592			18.32	-3.42	10.30	-1.07
Avg			327.5	458			35.29	-2.65	6.54	-1.43
Plate A	NM_016621	PHF21A	381	441	N	N	72.97	-0.61	20.18	-1.04
Plate B			505	556			77.23	-0.23	6.12	-1.25
Avg			443	498.5			75.10	-0.42	13.15	-1.14
Plate A	NM_001003819	TRIM6-TRIM34	389	394	N	N	69.67	-0.82	21.57	-0.98
Plate B			520	720			79.04	-0.14	48.19	0.53
Avg			454.5	557			74.35	-0.48	34.88	-0.23
Plate A	XM_926130	LOC642678	825	870	N	N	81.94	-0.07	37.01	-0.32
Plate B			1129	656			98.94	0.94	21.04	-0.62
Avg			977	763			90.44	0.44	29.02	-0.47
Plate A	NM_001002266	MARCH8	308	954	N	N	79.87	-0.20	69.92	1.09
Plate B			579	630			88.95	0.40	30.79	-0.20
Avg			443.5	792			84.41	0.10	50.35	0.44
Plate A	NM_032205	PHF20L1	408	397	N	N	93.63	0.64	26.45	-0.77
Plate B			338	810			70.71	-0.59	35.31	-0.01
Avg			373	603.5			82.17	0.03	30.88	-0.39

**Plate A**	NM_173557	RNF152	461	451	N	N	91.54	0.51	36.36	-0.35
**Plate B**			422	463			78.20	-0.18	32.40	-0.14
**Avg**			441.5	457			84.87	0.17	34.38	-0.24
**Plate A**	NM_001001740	RFWD2	487	48	N	N	97.54	0.88	22.92	-0.92
**Plate B**			1162	766			94.92	0.72	84.99	2.08
**Avg**			824.5	407			96.23	0.80	53.95	0.58
**Plate A**	NM_133368	RSPRY1	278	340	N	N	80.22	-0.17	41.18	-0.14
**Plate B**			570	428			78.95	-0.14	10.05	-1.08
**Avg**			424	384			79.58	-0.16	25.61	-0.61
**Plate A**	NM_001037125	UNKL	669	299	N	N	74.89	-0.50	23.75	-0.89
**Plate B**			739	706			84.17	0.14	22.52	-0.55
**Avg**			704	502.5			79.53	-0.18	23.13	-0.72
**Plate A**	NM_012216	MID2	456	798	N	N	57.24	-1.57	15.66	-1.23
**Plate B**			730	839			58.22	-1.26	11.44	-1.02
**Avg**			593	818.5			57.73	-1.42	13.55	-1.13
**Plate A**	NM_005937	MLLT6	508	624	N	N	88.19	0.31	8.17	-1.55
**Plate B**			788	348			89.59	0.44	3.74	-1.35
**Avg**			648	486			88.89	0.37	5.95	-1.45
**Plate A**	NM_006624	ZMYND11	140	252	N	N	87.14	0.25	28.97	-0.67
**Plate B**			154	298			81.17	-0.02	11.07	-1.04
**Avg**			147	275			84.16	0.11	20.02	-0.85
**Plate A**	NM_001165	BIRC3	830	301	N	N	90.84	0.47	10.63	-1.45
**Plate B**			688	956			79.51	-0.11	35.25	-0.02
**Avg**			759	628.5			85.17	0.18	22.94	-0.73
**Plate A**	NM_080875	MIB2	459	484	N	N	91.29	0.50	26.65	-0.76
**Plate B**			979	499			98.47	0.92	19.44	-0.68
**Avg**			719	491.5			94.88	0.71	23.05	-0.72
**Plate A**	NM_018207	TRIM62	871	1129	N	N	89.67	0.40	73.87	1.26
**Plate B**			262	914			40.46	-2.22	26.15	-0.40
**Avg**			566.5	1021.5			65.06	-0.91	50.01	0.43
**Plate A**	NM_017831	RNF125	776	982	N	N	97.42	0.87	57.74	0.57
**Plate B**			599	1121			84.81	0.18	47.90	0.52
**Avg**			687.5	1051.5			91.12	0.52	52.82	0.54
**Plate A**	NM_005180	BMI1	541	884	N	N	92.24	0.56	78.28	1.44
**Plate B**			1000	1088			96.70	0.82	64.25	1.21
**Avg**			770.5	986			94.47	0.69	71.26	1.33
**Plate A**	NM_016436	PHF20	621	624	N	N	90.82	0.47	40.54	-0.17
**Plate B**			598	752			92.47	0.59	41.89	0.26
**Avg**			609.5	688			91.65	0.53	41.22	0.05
**Plate A**	NM_018683	ZNF313	346	869	N	N	68.79	-0.87	16.34	-1.21
**Plate B**			840	1021			68.69	-0.69	22.92	-0.54
**Avg**			593	945			68.74	-0.78	19.63	-0.87
**Plate A**	NM_152267	RNF185	526	672	N	N	56.46	-1.62	23.36	-0.91
**Plate B**			579	859			47.32	-1.85	24.56	-0.47
**Avg**			552.5	765.5			51.89	-1.73	23.96	-0.69
**Plate A**	NM_032173	ZNRF3	499	530	N	N	49.90	-2.02	11.13	-1.43
**Plate B**			290	553			33.79	-2.58	15.19	-0.86
**Avg**			394.5	541.5			41.85	-2.30	13.16	-1.15
**Plate A**	NM_006618	JARID1B	189	143	N	N	65.61	-1.06	44.06	-0.02
**Plate B**			291	156			80.07	-0.08	25.64	-0.42
**Avg**			240	149.5			72.84	-0.57	34.85	-0.22
**Plate A**	NM_022064	RNF123	645	325	N	N	92.56	0.58	47.69	0.14
**Plate B**			730	658			83.42	0.10	29.48	-0.26
**Avg**			687.5	491.5			87.99	0.34	38.59	-0.06
**Plate A**	NM_001040444	PHF11	275	451	N	N	82.91	-0.01	57.43	0.55
**Plate B**			641	966			81.90	0.02	52.80	0.73
**Avg**			458	708.5			82.41	0.00	55.11	0.64
**Plate A**	NM_152620	TRIM60	626	389	N	N	78.59	-0.27	20.05	-1.05
**Plate B**			949	431			91.57	0.54	13.92	-0.92
**Avg**			787.5	410			85.08	0.13	16.99	-0.98
**Plate A**	NM_030912	TRIM8	229	591	N	N	98.25	0.92	89.68	1.93
**Plate B**			403	677			92.06	0.57	72.08	1.54
**Avg**			316	634			95.16	0.75	80.88	1.74
**Plate A**	NM_024787	RNF122	435	438	N	N	74.02	-0.55	44.98	0.02
**Plate B**			257	730			47.08	-1.86	49.45	0.58
**Avg**			346	584			60.55	-1.21	47.21	0.30
**Plate A**	NM_025236	RNF39	286	173	N	N	87.41	0.26	39.31	-0.22
**Plate B**			848	336			91.27	0.53	49.11	0.57
**Avg**			567	254.5			89.34	0.39	44.21	0.17
**Plate A**	NM_016120	RNF12	575	607	N	N	38.61	-2.70	7.74	-1.57
**Plate B**			907	1141			57.44	-1.30	46.19	0.45
**Avg**			741	874			48.03	-2.00	26.97	-0.56
**Plate A**	NM_197939	RNF135	1060	1061	N	N	69.43	-0.83	53.16	0.37
**Plate B**			722	455			42.24	-2.12	10.99	-1.04
**Avg**			891	758			55.84	-1.48	32.07	-0.34

Plate A	NM_147128	ZNRF2	699	668	N	N	88.98	0.36	22.31	-0.95
Plate B			665	693			85.11	0.19	8.66	-1.14
Avg			682	680.5			87.05	0.28	15.48	-1.04
Plate A	NM_203304	RKHD1	344	709	N	N	93.31	0.62	71.37	1.15
Plate B			587	501			95.40	0.75	30.74	-0.21
Avg			465.5	605			94.36	0.69	51.05	0.47
Plate A	NM_001004342	TRIM67	926	927	N	N	90.06	0.42	29.23	-0.65
Plate B			628	597			57.17	-1.32	22.28	-0.56
Avg			777	762			73.62	-0.45	25.76	-0.61
Plate A	XM_929433	LOC644006	261	286	N	N	47.13	-2.18	48.25	0.16
Plate B			497	440			79.48	-0.11	42.73	0.30
Avg			379	363			63.30	-1.15	45.49	0.23
Plate A	NM_032335	PHF6	305	355	N	N	97.70	0.89	78.59	1.46
Plate B			291	427			94.50	0.70	63.00	1.16
Avg			298	391			96.10	0.79	70.79	1.31
Plate A	NM_032588	TRIM63	279	460	N	N	96.06	0.79	75.65	1.33
Plate B			239	292			92.89	0.61	20.89	-0.62
Avg			259	376			94.47	0.70	48.27	0.36
Plate A	NM_001013734	RFPL4B	333	888	N	N	69.37	-0.83	75.79	1.34
Plate B			564	512			84.40	0.15	32.23	-0.14
Avg			448.5	700			76.88	-0.34	54.01	0.60
Plate A	NM_015009	PDZRN3	407	327	N	N	87.47	0.27	65.75	0.91
Plate B			573	832			84.29	0.15	68.39	1.38
Avg			490	579.5			85.88	0.21	67.07	1.15
Plate A	NM_013397	C6ORF49	651	478	N	N	78.96	-0.25	48.12	0.15
Plate B			400	621			66.75	-0.80	36.07	0.02
Avg			525.5	549.5			72.85	-0.53	42.09	0.09
Plate A	NM_032758	PHF5A	343	328	N	N	81.63	-0.09	56.40	0.51
Plate B			180	467			63.33	-0.98	28.48	-0.30
Avg			261.5	397.5			72.48	-0.54	42.44	0.10
Plate A	NM_006462	C20ORF18	769	615	N	N	66.19	-1.03	8.78	-1.53
Plate B			473	845			55.81	-1.39	25.56	-0.42
Avg			621	730			61.00	-1.21	17.17	-0.98
Plate A	NM_001105575	TRIM75	318	412	N	N	70.13	-0.79	57.52	0.56
Plate B			311	408			72.99	-0.46	54.41	0.79
Avg			314.5	410			71.56	-0.62	55.97	0.68
Plate A	NM_024900	PHF17	471	593	N	N	81.95	-0.07	47.05	0.11
Plate B			676	649			92.60	0.60	79.82	1.87
Avg			573.5	621			87.28	0.26	63.43	0.99
Plate A	NM_152616	TRIM42	374	402	N	N	87.70	0.28	42.29	-0.10
Plate B			608	493			96.71	0.82	55.38	0.83
Avg			491	447.5			92.21	0.55	48.83	0.37
Plate A	NM_014952	BAHD1	491	664	N	N	14.26	-4.18	11.60	-1.41
Plate B			570	177			31.58	-2.70	2.26	-1.41
Avg			530.5	420.5			22.92	-3.44	6.93	-1.41
Plate A	NM_032259	WDR24	266	521	N	N	91.35	0.50	57.97	0.58
Plate B			659	1022			96.81	0.83	80.63	1.90
Avg			462.5	771.5			94.08	0.66	69.30	1.24
Plate A	NM_015177	DTX4	533	122	N	N	98.31	0.92	72.95	1.22
Plate B			313	365			96.49	0.81	68.77	1.40
Avg			423	243.5			97.40	0.87	70.86	1.31
Plate A	NM_003449	TRIM26	574	612	N	N	83.62	0.03	72.22	1.19
Plate B			884	1033			85.07	0.19	74.73	1.65
Avg			729	822.5			84.35	0.11	73.48	1.42
Plate A	NM_138415	PHF21B	259	530	N	N	59.46	-1.43	53.40	0.38
Plate B			808	1221			76.24	-0.29	76.99	1.75
Avg			533.5	875.5			67.85	-0.86	65.19	1.06
Plate A	NM_020814	MARCH4	405	313	N	N	72.59	-0.64	39.94	-0.20
Plate B			218	699			59.63	-1.18	58.23	0.95
Avg			311.5	506			66.11	-0.91	49.08	0.38
Plate A	NM_019004	ANKIB1	927	574	N	N	84.36	0.08	27.18	-0.74
Plate B			499	747			60.92	-1.11	13.25	-0.94
Avg			713	660.5			72.64	-0.52	20.22	-0.84
Plate A	NM_152635	OIT3	154	280	N	N	95.45	0.75	71.79	1.17
Plate B			400	475			98.50	0.92	81.05	1.92
Avg			277	377.5			96.98	0.83	76.42	1.54
Plate A	NM_015246	MGRN1	613	913	N	N	88.42	0.32	75.25	1.31
Plate B			428	845			94.63	0.71	78.46	1.81
Avg			520.5	879			91.52	0.52	76.85	1.56
Plate A	NM_014245	RNF7	513	676	N	N	81.68	-0.09	59.62	0.65
Plate B			706	942			82.72	0.06	66.45	1.30
Avg			609.5	809			82.20	-0.01	63.03	0.97
Plate A	NM_152528	WDSUB1	639	785	N	N	86.85	0.23	75.54	1.33
Plate B			1175	1159			98.98	0.94	79.81	1.87
Avg			907	972			92.92	0.59	77.68	1.60

**Plate A**	NM_032673	PCGF1	560	817	N	N	92.68	0.58	83.11	1.65
**Plate B**			327	696			97.86	0.88	61.35	1.09
**Avg**			443.5	756.5			95.27	0.73	72.23	1.37
**Plate A**	NM_006458	TRIM3	590	631	N	N	91.19	0.49	60.06	0.67
**Plate B**			711	1060			90.15	0.47	79.72	1.86
**Avg**			650.5	845.5			90.67	0.48	69.89	1.26
**Plate A**	NM_152577	ZNF645	889	479	N	N	95.28	0.74	63.05	0.79
**Plate B**			1064	930			96.33	0.80	58.17	0.95
**Avg**			976.5	704.5			95.81	0.77	60.61	0.87
**Plate A**	NM_006913	RNF5	684	727	N	N	47.66	-2.15	49.38	0.21
**Plate B**			693	878			63.78	-0.96	43.28	0.32
**Avg**			688.5	802.5			55.72	-1.56	46.33	0.27
**Plate A**	NM_015288	PHF15	1029	223	N	N	71.53	-0.70	30.94	-0.58
**Plate B**			823	908			67.31	-0.77	38.44	0.12
**Avg**			926	565.5			69.42	-0.74	34.69	-0.23
**Plate A**	NM_007298	BRCA1	591	500	N	N	49.58	-2.04	15.00	-1.26
**Plate B**			658	830			50.91	-1.66	51.57	0.67
**Avg**			624.5	665			50.24	-1.85	33.28	-0.29
**Plate A**	NM_019006	ZFAND6	891	887	N	N	97.53	0.01	85.34	0.66
**Plate B**			997	1647			94.98	0.77	92.90	1.23
**Avg**			944	1267			96.26	0.39	89.12	0.95
**Plate A**	NM_152550	SH3RF2	1103	1086	N	N	93.02	-0.42	82.50	0.53
**Plate B**			364	1891			50.82	-1.71	93.60	1.27
**Avg**			733.5	1488.5			71.92	-1.06	88.05	0.90
**Plate A**	NM_020724	RNF150	509	754	N	N	96.07	-0.13	68.44	-0.15
**Plate B**			1288	847			79.27	-0.11	63.52	-0.13
**Avg**			898.5	800.5			87.67	-0.12	65.98	-0.14
**Plate A**	NM_006315	PCGF3	553	1338	N	N	99.10	0.16	95.59	1.16
**Plate B**			1145	1760			91.53	0.57	96.42	1.40
**Avg**			849	1549			95.31	0.37	96.01	1.28
**Plate A**	NM_183078	RNF8	1075	1101	N	N	98.23	0.08	91.64	0.97
**Plate B**			544	1908			64.89	-0.92	93.97	1.28
**Avg**			809.5	1504.5			81.56	-0.42	92.81	1.13
**Plate A**	NM_194329	RNF38	674	1454	N	N	95.40	-0.19	85.90	0.69
**Plate B**			913	937			69.11	-0.68	57.84	-0.39
**Avg**			793.5	1195.5			82.26	-0.44	71.87	0.15
**Plate A**	NM_033229	TRIM15	946	878	N	N	98.84	0.14	75.63	0.19
**Plate B**			1126	367			94.85	0.76	34.06	-1.49
**Avg**			1036	622.5			96.84	0.45	54.84	-0.65
**Plate A**	NM_015649	IRF2BP1	233	671	N	N	84.12	-1.27	65.28	-0.31
**Plate B**			405	357			63.46	-1.00	44.26	-1.02
**Avg**			319	514			73.79	-1.14	54.77	-0.66
**Plate A**	NM_003482	MLL2	895	569	N	N	19.89	-7.42	4.57	-3.24
**Plate B**			218	634			5.96	-4.22	4.42	-2.86
**Avg**			556.5	601.5			12.93	-5.82	4.49	-3.05
**Plate A**	NM_015089	PARC	932	1130	N	N	97.85	0.04	66.37	-0.25
**Plate B**			972	1307			83.74	0.14	56.16	-0.47
**Avg**			952	1218.5			90.80	0.09	61.27	-0.36
**Plate A**	NM_018411	HR	1827	1712	N	N	95.57	-0.18	80.84	0.45
**Plate B**			1465	1945			62.12	-1.07	90.08	1.10
**Avg**			1646	1828.5			78.84	-0.63	85.46	0.77
**Plate A**	NM_024297	PHF23	1555	1379	N	N	99.29	0.18	91.08	0.94
**Plate B**			1440	1212			96.74	0.87	92.99	1.24
**Avg**			1497.5	1295.5			98.01	0.52	92.03	1.09
**Plate A**	NM_022453	RNF25	332	248	N	N	94.88	-0.24	68.55	-0.15
**Plate B**			217	745			85.71	0.25	79.46	0.61
**Avg**			274.5	496.5			90.30	0.00	74.01	0.23
**Plate A**	NM_153812	PHF13	1454	1820	N	N	99.31	0.18	86.87	0.74
**Plate B**			1255	1541			61.83	-1.09	74.11	0.36
**Avg**			1354.5	1680.5			80.57	-0.45	80.49	0.55
**Plate A**	NM_138287	DTX3L	347	605	N	N	94.81	-0.25	75.37	0.18
**Plate B**			401	711			73.32	-0.45	70.32	0.19
**Avg**			374	658			84.06	-0.35	72.85	0.19
**Plate A**	NM_006290	TNFAIP3	935	471	N	N	98.18	0.07	90.02	0.89
**Plate B**			1167	667			82.26	0.05	91.90	1.19
**Avg**			1051	569			90.22	0.06	90.96	1.04
**Plate A**	NM_005664	MKRN3	1655	561	N	N	97.40	0.00	29.77	-2.02
**Plate B**			1629	1409			91.53	0.57	58.27	-0.37
**Avg**			1642	985			94.47	0.29	44.02	-1.20
**Plate A**	NM_001080535	LINCR	1660	1556	N	N	95.48	-0.18	80.91	0.45
**Plate B**			820	1166			68.66	-0.71	50.09	-0.75
**Avg**			1240	1361			82.07	-0.45	65.50	-0.15
**Plate A**	NM_020889	PHF12	703	371	N	N	94.74	-0.26	82.48	0.53
**Plate B**			957	1240			86.94	0.32	66.45	0.01
**Avg**			830	805.5			90.84	0.03	74.47	0.27

**Plate A**	NM_033290	MID1	1015	1700	N	N	91.43	-0.57	81.76	0.49
**Plate B**			1443	1871			80.39	-0.05	46.18	-0.93
**Avg**			1229	1785.5			85.91	-0.31	63.97	-0.22
**Plate A**	NM_014788	TRIM14	970	879	N	N	99.38	0.19	76.68	0.24
**Plate B**			1732	1511			88.45	0.40	78.36	0.56
**Avg**			1351	1195			93.92	0.30	77.52	0.40
**Plate A**	NM_001040452	RUFY1	993	1119	N	N	79.46	-1.72	46.11	-1.23
**Plate B**			753	1333			28.02	-2.99	44.04	-1.03
**Avg**			873	1226			53.74	-2.35	45.07	-1.13
**Plate A**	NM_014487	ZNF330	847	506	N	N	97.40	0.00	26.28	-2.19
**Plate B**			1515	802			89.97	0.49	43.52	-1.05
**Avg**			1181	654			93.68	0.24	34.90	-1.62
**Plate A**	NM_198085	RNF148	1246	1354	N	N	97.51	0.01	78.21	0.32
**Plate B**			910	948			67.58	-0.77	55.70	-0.49
**Avg**			1078	1151			82.55	-0.38	66.95	-0.08
**Plate A**	NM_080631	VPS41	1123	1282	N	N	97.42	0.00	79.56	0.38
**Plate B**			1311	1389			84.74	0.19	67.39	0.05
**Avg**			1217	1335.5			91.08	0.10	73.47	0.22
**Plate A**	NM_006048	UBE4B	1062	225	N	N	93.22	-0.40	58.22	-0.65
**Plate B**			1771	931			80.29	-0.06	47.15	-0.88
**Avg**			1416.5	578			86.76	-0.23	52.69	-0.77
**Plate A**	NM_004591	CCL20	1504	484	N	N	97.74	0.03	74.17	0.12
**Plate B**			1146	1777			68.59	-0.71	72.88	0.31
**Avg**			1325	1130.5			83.16	-0.34	73.52	0.22
**Plate A**	NM_016422	RNF141	1592	864	N	N	71.80	-2.45	17.25	-2.63
**Plate B**			1487	1553			41.16	-2.25	30.26	-1.66
**Avg**			1539.5	1208.5			56.48	-2.35	23.75	-2.15
**Plate A**	NM_017778	WHSC1L1	1695	863	N	N	94.81	-0.25	42.06	-1.43
**Plate B**			928	750			50.00	-1.75	26.13	-1.85
**Avg**			1311.5	806.5			72.40	-1.00	34.10	-1.64
**Plate A**	NM_001005416	MARCH2	821	931	N	N	95.01	-0.23	58.54	-0.63
**Plate B**			1327	758			81.24	0.00	48.55	-0.82
**Avg**			1074	844.5			88.12	-0.12	53.54	-0.73
**Plate A**	NM_005831	NDP52	1034	633	N	N	99.52	0.20	90.52	0.91
**Plate B**			470	659			88.30	0.39	87.86	1.00
**Avg**			752	646			93.91	0.30	89.19	0.96
**Plate A**	NM_015255	UBR2	1032	826	N	N	98.84	0.14	70.22	-0.07
**Plate B**			2310	1556			96.67	0.86	72.43	0.29
**Avg**			1671	1191			97.75	0.50	71.32	0.11
**Plate A**	NM_015271	TRIM2	1170	1527	N	N	99.40	0.19	94.24	1.09
**Plate B**			2094	1703			95.56	0.80	92.25	1.20
**Avg**			1632	1615			97.48	0.50	93.24	1.15
**Plate A**	NM_005667	RNF103	1296	1550	N	N	98.15	0.07	71.94	0.02
**Plate B**			2023	1478			83.54	0.13	60.42	-0.27
**Avg**			1659.5	1514			90.84	0.10	66.18	-0.13
**Plate A**	NM_178556	TRIML1	751	372	N	N	98.27	0.08	38.44	-1.61
**Plate B**			715	880			78.74	-0.14	66.02	-0.01
**Avg**			733	626			88.51	-0.03	52.23	-0.81
**Plate A**	NM_005802	TOPORS	998	932	N	N	98.40	0.09	54.94	-0.81
**Plate B**			880	1413			74.55	-0.38	59.87	-0.29
**Avg**			939	1172.5			86.47	-0.14	57.40	-0.55
**Plate A**	NM_007331	WHSC1	953	1328	N	N	97.69	0.03	71.01	-0.03
**Plate B**			1664	1072			96.69	0.86	70.43	0.19
**Avg**			1308.5	1200			97.19	0.45	70.72	0.08
**Plate A**	NM_001024941	TRIM17	697	1140	N	N	62.12	-3.38	67.28	-0.21
**Plate B**			591	408			22.50	-3.30	12.75	-2.47
**Avg**			644	774			42.31	-3.34	40.01	-1.34
**Plate A**	NM_033020	TRIM33	1610	588	N	N	90.25	-0.69	37.59	-1.65
**Plate B**			927	634			68.28	-0.73	14.20	-2.41
**Avg**			1268.5	611			79.27	-0.71	25.89	-2.03
**Plate A**	NM_018835	RC3H2	1374	900	N	N	87.34	-0.96	39.89	-1.54
**Plate B**			1299	1361			67.59	-0.77	52.53	-0.63
**Avg**			1336.5	1130.5			77.46	-0.87	46.21	-1.08
**Plate A**	XM_927169	LOC643904	1262	1230	N	N	95.96	-0.14	73.82	0.11
**Plate B**			963	1422			73.31	-0.45	60.83	-0.25
**Avg**			1112.5	1326			84.64	-0.29	67.33	-0.07
**Plate A**	NM_138700	TRIM40	1460	1333	N	N	98.22	0.08	67.29	-0.21
**Plate B**			1152	1470			68.66	-0.71	68.91	0.12
**Avg**			1306	1401.5			83.44	-0.32	68.10	-0.04
**Plate A**	NM_001098527	RFPL2	798	694	N	N	98.50	0.10	70.46	-0.06
**Plate B**			1565	644			96.29	0.84	82.14	0.74
**Avg**			1181.5	669			97.40	0.47	76.30	0.34
**Plate A**	NM_001077445	PHF16	860	1586	N	N	98.37	0.09	86.07	0.70
**Plate B**			997	547			93.58	0.69	78.61	0.57
**Avg**			928.5	1066.5			95.98	0.39	82.34	0.64

**Plate A**	NM_006074	TRIM22	2130	2363	N	N	97.42	0.00	86.37	0.71
**Plate B**			949	1808			61.75	-1.10	76.83	0.49
**Avg**			1539.5	2085.5			79.58	-0.55	81.60	0.60
**Plate A**	NM_017999	RNF31	1501	1788	N	N	81.01	-1.57	59.68	-0.58
**Plate B**			1044	1411			33.62	-2.67	57.41	-0.41
**Avg**			1272.5	1599.5			57.32	-2.12	58.54	-0.49
**Plate A**	NM_181710	ZNRF4	1015	556	N	N	99.01	0.15	55.58	-0.78
**Plate B**			1122	945			91.35	0.56	78.94	0.59
**Avg**			1068.5	750.5			95.18	0.36	67.26	-0.09
**Plate A**	NM_178841	RNF166	2050	1222	N	N	92.20	-0.50	38.87	-1.58
**Plate B**			1501	931			70.15	-0.62	31.47	-1.61
**Avg**			1775.5	1076.5			81.17	-0.56	35.17	-1.60
**Plate A**	XM_938138	LOC649055	945	1742	N	N	97.57	0.01	72.50	0.04
**Plate B**			876	804			81.74	0.03	40.42	-1.19
**Avg**			910.5	1273			89.65	0.02	56.46	-0.58
**Plate A**	NM_152617	RNF168	850	907	N	N	86.12	-1.08	27.23	-2.15
**Plate B**			1134	1612			63.76	-0.98	32.44	-1.56
**Avg**			992	1259.5			74.94	-1.03	29.84	-1.85
**Plate A**	XM_374917	LOC399937	963	663	N	N	98.23	0.08	68.63	-0.14
**Plate B**			1240	1459			88.31	0.39	79.92	0.63
**Avg**			1101.5	1061			93.27	0.24	74.27	0.24
**Plate A**	NM_138800	TRIM43	862	1830	N	N	96.52	-0.09	88.80	0.83
**Plate B**			907	1668			74.42	-0.39	73.02	0.31
**Avg**			884.5	1749			85.47	-0.24	80.91	0.57
**Plate A**	NM_007148	ZNF179	799	209	N	N	97.00	-0.04	43.06	-1.38
**Plate B**			1003	705			94.32	0.73	48.65	-0.81
**Avg**			901	457			95.66	0.35	45.86	-1.10
**Plate A**	NM_020165	RAD18	1211	1500	N	N	99.01	0.15	97.53	1.25
**Plate B**			1592	1041			91.46	0.57	94.91	1.33
**Avg**			1401.5	1270.5			95.23	0.36	96.22	1.29
**Plate A**	NM_005861	STUB1	1468	1318	N	N	96.46	-0.09	63.58	-0.39
**Plate B**			1269	1177			87.31	0.34	53.61	-0.58
**Avg**			1368.5	1247.5			91.89	0.12	58.60	-0.49
**Plate A**	NM_005885	MARCH6	847	1349	N	N	98.70	0.12	85.69	0.68
**Plate B**			1365	1898			95.82	0.82	89.67	1.08
**Avg**			1106	1623.5			97.26	0.47	87.68	0.88
**Plate A**	NM_000465	BARD1	1826	640	N	N	99.78	0.23	91.88	0.98
**Plate B**			1314	1637			93.07	0.66	95.66	1.36
**Avg**			1570	1138.5			96.43	0.44	93.77	1.17
**Plate A**	NM_001009922	RCHY1	1436	1413	N	N	97.56	0.01	63.62	-0.39
**Plate B**			1519	1438			81.90	0.03	49.51	-0.77
**Avg**			1477.5	1425.5			89.73	0.02	56.57	-0.58
**Plate A**	NM_005879	TRIP	937	751	N	N	89.33	-0.77	54.46	-0.83
**Plate B**			1038	1146			70.81	-0.59	72.69	0.30
**Avg**			987.5	948.5			80.07	-0.68	63.57	-0.27
**Plate A**	NM_033342	TRIM7	2377	2198	N	N	96.47	-0.09	71.84	0.01
**Plate B**			1313	2078			81.34	0.00	64.63	-0.07
**Avg**			1845	2138			88.90	-0.04	68.23	-0.03
**Plate A**	NM_006150	LMO6	883	1024	N	N	97.96	0.05	71.88	0.01
**Plate B**			537	779			75.05	-0.35	53.02	-0.61
**Avg**			710	901.5			86.50	-0.15	62.45	-0.30
**Plate A**	NM_032765	TRIM52	1093	1489	N	N	98.81	0.13	95.16	1.14
**Plate B**			551	1722			82.21	0.05	95.01	1.33
**Avg**			822	1605.5			90.51	0.09	95.09	1.23
**Plate A**	NM_012116	CBLC	1336	698	N	N	99.33	0.18	88.11	0.80
**Plate B**			1784	1264			96.69	0.86	89.48	1.07
**Avg**			1560	981			98.01	0.52	88.79	0.94
**Plate A**	NM_014901	RNF44	865	669	N	N	94.91	-0.24	77.58	0.29
**Plate B**			1355	1789			80.00	-0.07	90.16	1.11
**Avg**			1110	1229			87.46	-0.16	83.87	0.70
**Plate A**	NM_170606	MLL3	1647	913	N	N	98.79	0.13	58.82	-0.62
**Plate B**			950	1634			92.74	0.64	82.31	0.74
**Avg**			1298.5	1273.5			95.76	0.39	70.57	0.06
**Plate A**	NM_173647	RNF149	1570	1376	N	N	98.92	0.14	71.37	-0.01
**Plate B**			1814	1190			91.90	0.59	70.84	0.21
**Avg**			1692	1283			95.41	0.37	71.10	0.10
**Plate A**	NM_001005207	TRIM37	1903	1481	N	N	94.06	-0.32	41.39	-1.46
**Plate B**			1747	1609			85.23	0.22	54.94	-0.52
**Avg**			1825	1545			89.65	-0.05	48.17	-0.99
**Plate A**	NM_022161	BIRC7	1347	1142	N	N	96.59	-0.08	85.73	0.68
**Plate B**			1057	1037			78.43	-0.16	66.63	0.02
**Avg**			1202	1089.5			87.51	-0.12	76.18	0.35
**Plate A**	NM_170662	CBLB	2165	1076	N	N	93.26	-0.40	36.06	-1.72
**Plate B**			1422	1585			70.75	-0.59	39.87	-1.22
**Avg**			1793.5	1330.5			82.00	-0.49	37.97	-1.47

**Plate A**	NM_183043	RNF6	1316	574	N	N	90.43	-0.67	20.73	-2.46
**Plate B**			1490	1299			63.42	-1.00	35.95	-1.40
**Avg**			1403	936.5			76.92	-0.84	28.34	-1.93
**Plate A**	NM_014746	RNF144	716	812	N	N	97.49	0.01	57.88	-0.66
**Plate B**			1359	883			95.29	0.79	62.74	-0.16
**Avg**			1037.5	847.5			96.39	0.40	60.31	-0.41
**Plate A**	NM_152271	LONRF1	1157	938	N	N	95.25	-0.21	46.06	-1.24
**Plate B**			1636	2357			84.17	0.16	89.73	1.09
**Avg**			1396.5	1647.5			89.71	-0.02	67.89	-0.08
**Plate A**	NM_184087	TRIM55	1326	914	N	N	97.89	0.05	87.75	0.78
**Plate B**			790	500			70.76	-0.59	68.00	0.08
**Avg**			1058	707			84.32	-0.27	77.87	0.43
**Plate A**	NM_015695	BRPF3	797	619	N	N	98.75	0.13	71.89	0.01
**Plate B**			2173	679			95.58	0.80	53.61	-0.58
**Avg**			1485	649			97.16	0.46	62.75	-0.29
**Plate A**	NM_001017368	RFFL	616	1088	N	N	95.29	-0.20	80.79	0.44
**Plate B**			884	333			73.64	-0.43	49.85	-0.76
**Avg**			750	710.5			84.47	-0.32	65.32	-0.16
**Plate A**	NM_001005374	LRSAM1	872	868	N	N	99.31	0.18	71.77	0.01
**Plate B**			796	1519			90.95	0.54	81.37	0.70
**Avg**			834	1193.5			95.13	0.36	76.57	0.35
**Plate A**	NM_007218	RNF139	1622	1163	N	N	99.20	0.17	92.35	1.00
**Plate B**			1058	217			90.93	0.54	92.17	1.20
**Avg**			1340	690			95.06	0.36	92.26	1.10
**Plate A**	NM_032645	RAPSN	1730	1241	N	N	92.31	-0.49	64.30	-0.35
**Plate B**			1978	1587			84.18	0.16	49.65	-0.77
**Avg**			1854	1414			88.24	-0.16	56.98	-0.56
**Plate A**	NM_022368	PJA1	931	1091	N	N	85.39	-1.15	70.30	-0.06
**Plate B**			1113	1178			69.99	-0.63	47.62	-0.86
**Avg**			1022	1134.5			77.69	-0.89	58.96	-0.46
**Plate A**	NM_016626	RKHD2	1226	1304	N	N	93.64	-0.36	71.47	-0.01
**Plate B**			754	1196			64.32	-0.95	73.75	0.35
**Avg**			990	1250			78.98	-0.66	72.61	0.17
**Plate A**	NM_004647	DPF1	2062	2587	N	N	98.06	0.31	77.12	0.27
**Plate B**			1643	1880			95.07	0.17	67.93	-0.12
**Avg**			1852.5	2233.5			96.57	0.24	72.52	0.07
**Plate A**	NM_004788	UBE4A	2216	2145	N	N	98.47	0.35	83.03	0.64
**Plate B**			1335	2278			92.43	-0.21	78.93	0.43
**Avg**			1775.5	2211.5			95.45	0.07	80.98	0.53
**Plate A**	NM_178502	DTX3	1951	3071	N	N	99.18	0.43	92.02	1.20
**Plate B**			1397	1880			97.14	0.46	88.03	0.88
**Avg**			1674	2475.5			98.16	0.45	90.03	1.04
**Plate A**	NM_001144	AMFR	1234	2517	N	N	97.00	0.20	66.47	-0.40
**Plate B**			1802	1825			94.95	0.15	85.32	0.75
**Avg**			1518	2171			95.98	0.18	75.89	0.17
**Plate A**	NM_003843	SCEL	2749	1989	N	N	99.64	0.47	73.05	0.01
**Plate B**			1412	1603			92.71	-0.17	74.80	0.22
**Avg**			2080.5	1796			96.17	0.15	73.92	0.12
**Plate A**	NM_017824	MARCH5	1581	1432	N	N	97.09	0.21	58.10	-0.93
**Plate B**			2399	1947			97.00	0.45	77.76	0.37
**Avg**			1990	1689.5			97.04	0.33	67.93	-0.28
**Plate A**	XM_942113	LOC652591	1129	1700	N	N	87.60	-0.78	80.06	0.45
**Plate B**			1773	1430			94.92	0.15	82.24	0.59
**Avg**			1451	1565			91.26	-0.32	81.15	0.52
**Plate A**	NM_005921	MAP3K1	2291	2979	N	N	97.12	0.21	54.28	-1.17
**Plate B**			2079	1517			95.09	0.17	42.32	-1.39
**Avg**			2185	2248			96.11	0.19	48.30	-1.28
**Plate A**	NM_018150	C1orf164	2888	2499	N	N	95.81	0.07	57.30	-0.98
**Plate B**			1577	1493			86.62	-1.04	47.69	-1.13
**Avg**			2232.5	1996			91.22	-0.48	52.50	-1.05
**Plate A**	XM_062300	LOC120824	1979	2167	N	N	87.32	-0.81	65.67	-0.45
**Plate B**			2377	1309			91.96	-0.27	55.08	-0.76
**Avg**			2178	1738			89.64	-0.54	60.37	-0.61
**Plate A**	NM_001102562	LOC441061	2304	3658	N	N	88.50	-0.69	73.48	0.04
**Plate B**			2946	2211			86.08	-1.11	46.18	-1.20
**Avg**			2625	2934.5			87.29	-0.90	59.83	-0.58
**Plate A**	NM_007358	M96	578	706	Y*	N	92.21	-0.30	84.84	0.75
**Plate B**			952	404			95.59	0.24	86.88	0.82
**Avg**			765	555			93.90	-0.03	85.86	0.79
**Plate A**	NM_014771	RNF40	2725	2879	N	N	97.83	0.29	74.47	0.10
**Plate B**			1773	1648			95.88	0.29	60.98	-0.46
**Avg**			2249	2263.5			96.86	0.29	67.73	-0.18
**Plate A**	NM_019592	RNF20	806	2350	N	N	88.83	-0.66	62.34	-0.66
**Plate B**			2278	1540			94.69	0.12	77.34	0.35
**Avg**			1542	1945			91.76	-0.27	69.84	-0.16

**Plate A**	NM_001007157	PHF14	1310	2454	N	N	97.33	0.23	84.03	0.70
**Plate B**			565	1554			93.81	-0.01	74.20	0.19
**Avg**			937.5	2004			95.57	0.11	79.11	0.45
**Plate A**	XM_001131933	LOC729974	1327	1606	N	N	99.25	0.43	91.59	1.18
**Plate B**			1182	1412			97.88	0.57	90.58	1.01
**Avg**			1254.5	1509			98.57	0.50	91.09	1.09
**Plate A**	NM_052916	RNF157	1218	1692	N	N	93.19	-0.20	78.25	0.34
**Plate B**			1427	1311			94.95	0.15	81.24	0.54
**Avg**			1322.5	1501.5			94.07	-0.02	79.74	0.44
**Plate A**	NM_016494	LOC51255	2014	2969	N	N	88.48	-0.69	29.77	-2.71
**Plate B**			2830	2635			82.69	-1.60	20.91	-2.46
**Avg**			2422	2802			85.58	-1.14	25.34	-2.58
**Plate A**	NM_173631	ZNF547	1068	2664	N	N	82.96	-1.27	55.26	-1.11
**Plate B**			1399	2120			86.78	-1.01	53.21	-0.85
**Avg**			1233.5	2392			84.87	-1.14	54.23	-0.98
**Plate A**	NM_173082	SHPRH	2455	3246	N	N	78.25	-1.76	53.88	-1.19
**Plate B**			3211	2469			84.55	-1.33	24.14	-2.30
**Avg**			2833	2857.5			81.40	-1.55	39.01	-1.74
**Plate A**	NM_020901	KIAA1542	2867	3423	N	N	97.91	0.29	83.70	0.68
**Plate B**			3188	2107			97.62	0.53	78.64	0.41
**Avg**			3027.5	2765			97.76	0.41	81.17	0.55
**Plate A**	NM_152896	UHRF2	1435	2020	N	N	94.22	-0.09	75.59	0.17
**Plate B**			980	947			86.33	-1.08	84.48	0.70
**Avg**			1207.5	1483.5			90.27	-0.58	80.04	0.44
**Plate A**	NM_171982	TRIM35	2392	1752	N	N	96.03	0.10	84.76	0.75
**Plate B**			1685	2379			94.48	0.09	85.92	0.78
**Avg**			2038.5	2065.5			95.25	0.09	85.34	0.76
**Plate A**	NM_001008225	CNOT4	1606	2838	N	N	96.70	0.17	86.61	0.86
**Plate B**			2443	2825			95.95	0.30	87.89	0.87
**Avg**			2024.5	2831.5			96.32	0.23	87.25	0.87
**Plate A**	XM_929433	LOC644006	1802	1946	N	N	87.40	-0.80	75.90	0.19
**Plate B**			1989	2387			91.35	-0.36	84.50	0.71
**Avg**			1895.5	2166.5			89.38	-0.58	80.20	0.45
**Plate A**	NM_006604	RFPL3	2369	3180	N	N	91.52	-0.37	49.91	-1.44
**Plate B**			3380	2791			91.78	-0.30	69.97	-0.02
**Avg**			2874.5	2985.5			91.65	-0.34	59.94	-0.73
**Plate A**	NM_001273	CHD4	1576	1623	N	N	96.19	0.11	92.11	1.21
**Plate B**			1985	1070			97.33	0.49	93.93	1.17
**Avg**			1780.5	1346.5			96.76	0.30	93.02	1.19
**Plate A**	NM_025126	RNF34	1896	2341	N	N	79.43	-1.64	71.25	-0.10
**Plate B**			1885	1856			87.64	-0.89	59.64	-0.53
**Avg**			1890.5	2098.5			83.53	-1.26	65.45	-0.32
**Plate A**	XM_061890	TRIM64	2011	3363	N	N	81.75	-1.40	61.23	-0.73
**Plate B**			2430	1251			88.27	-0.80	25.10	-2.25
**Avg**			2220.5	2307			85.01	-1.10	43.16	-1.49
**Plate A**	NM_007150	ZNF185	1837	1942	N	N	81.33	-1.44	63.54	-0.59
**Plate B**			2196	2070			85.61	-1.18	63.14	-0.36
**Avg**			2016.5	2006			83.47	-1.31	63.34	-0.47
**Plate A**	NM_139048	SMARCA3	1835	3010	N	N	98.96	0.40	86.25	0.84
**Plate B**			1970	1127			95.84	0.28	64.33	-0.30
**Avg**			1902.5	2068.5			97.40	0.34	75.29	0.27
**Plate A**	NM_001102596	DTX2	1978	2845	N	N	93.98	-0.12	77.33	0.28
**Plate B**			2017	2839			93.95	0.01	79.85	0.47
**Avg**			1997.5	2842			93.97	-0.05	78.59	0.38
**Plate A**	NM_020774	MIB1	1813	2885	N	N	98.73	0.38	66.62	-0.39
**Plate B**			1413	2247			96.53	0.38	70.76	0.02
**Avg**			1613	2566			97.63	0.38	68.69	-0.19
**Plate A**	NM_030961	TRIM56	2178	1861	N	N	99.40	0.45	83.99	0.70
**Plate B**			1771	2164			98.25	0.62	87.01	0.83
**Avg**			1974.5	2012.5			98.83	0.54	85.50	0.76
**Plate A**	NM_015528	RNF167	1874	2391	N	N	94.18	-0.10	71.39	-0.09
**Plate B**			933	1506			90.57	-0.47	70.19	-0.01
**Avg**			1403.5	1948.5			92.38	-0.28	70.79	-0.05
**Plate A**	NM_198461	LONRF2	1530	1689	N	N	97.45	0.25	89.40	1.04
**Plate B**			1293	1555			97.99	0.59	92.99	1.13
**Avg**			1411.5	1622			97.72	0.42	91.20	1.08
**Plate A**	XM_925959	LOC642446	2640	2011	N	N	98.41	0.35	61.31	-0.73
**Plate B**			2688	2411			98.77	0.70	56.41	-0.69
**Avg**			2664	2211			98.59	0.52	58.86	-0.71
**Plate A**	NM_018124	RFWD3	1052	1099	N	N	56.65	-4.02	28.75	-2.77
**Plate B**			1483	950			62.31	-4.50	29.47	-2.03
**Avg**			1267.5	1024.5			59.48	-4.26	29.11	-2.40
**Plate A**	XM_936370	LOC642219	2676	2850	N	N	90.55	-0.48	71.05	-0.11
**Plate B**			2438	1490			82.69	-1.60	34.43	-1.79
**Avg**			2557	2170			86.62	-1.04	52.74	-0.95

**Plate A**	NM_174903	RNF151	740	1454	N	N	88.65	-0.67	50.41	-1.41
**Plate B**			931	1200			93.98	0.02	33.67	-1.82
**Avg**			835.5	1327			91.32	-0.33	42.04	-1.62
**Plate A**	NM_030581	WDR59	2053	2848	N	N	98.73	0.38	89.01	1.01
**Plate B**			1972	1486			97.72	0.55	85.06	0.73
**Avg**			2012.5	2167			98.23	0.46	87.04	0.87
**Plate A**	NM_021729	VPS11	1001	2423	N	N	93.51	-0.17	82.34	0.59
**Plate B**			2770	1200			95.42	0.22	87.08	0.83
**Avg**			1885.5	1811.5			94.46	0.03	84.71	0.71
**Plate A**	NM_014323	ZNF278	1491	2523	N	N	93.76	-0.14	88.70	0.99
**Plate B**			1564	1932			94.63	0.11	74.48	0.21
**Avg**			1527.5	2227.5			94.20	-0.02	81.59	0.60
**Plate A**	NM_006768	BRAP	1249	3315	N	N	98.40	0.34	85.82	0.81
**Plate B**			1995	1765			99.30	0.77	79.26	0.44
**Avg**			1622	2540			98.85	0.56	82.54	0.63
**Plate A**	NM_024546	C13ORF7	1975	2983	N	N	95.19	0.01	68.76	-0.26
**Plate B**			1911	2247			89.80	-0.58	75.12	0.24
**Avg**			1943	2615			92.49	-0.29	71.94	-0.01
**Plate A**	NM_198924	TRIM73	2245	1880	N	N	95.01	-0.01	72.13	-0.05
**Plate B**			1848	1725			96.10	0.32	55.83	-0.72
**Avg**			2046.5	1802.5			95.56	0.15	63.98	-0.38
**Plate A**	NM_182757	IBRDC2	917	1338	N	N	97.71	0.27	62.33	-0.66
**Plate B**			1074	765			99.53	0.81	74.25	0.20
**Avg**			995.5	1051.5			98.62	0.54	68.29	-0.23
**Plate A**	NM_148176	PPIL2	677	1817	N	N	89.22	-0.62	70.28	-0.16
**Plate B**			399	1039			81.95	-1.70	69.30	-0.05
**Avg**			538	1428			85.59	-1.16	69.79	-0.11
**Plate A**	NM_006321	ARIH2	1206	2454	N	N	60.12	-3.66	60.72	-0.76
**Plate B**			2795	2143			78.25	-2.23	36.58	-1.68
**Avg**			2000.5	2298.5			69.18	-2.94	48.65	-1.22
**Plate A**	NM_003852	TIF1	1861	1376	N	N	87.91	-0.75	47.17	-1.61
**Plate B**			1999	1999			86.49	-1.05	58.23	-0.60
**Avg**			1930	1687.5			87.20	-0.90	52.70	-1.11
**Plate A**	XM_001133292	LOC728919	2138	3322	N	N	95.51	0.04	76.19	0.21
**Plate B**			1492	1602			88.40	-0.78	53.56	-0.83
**Avg**			1815	2462			91.96	-0.37	64.87	-0.31
**Plate A**	NM_003300	TRAF3	1927	2708	N	N	97.20	0.22	67.61	-0.33
**Plate B**			2984	2152			97.39	0.50	62.17	-0.41
**Avg**			2455.5	2430			97.29	0.36	64.89	-0.37
**Plate A**	NM_033017	TRIM4	1505	3474	N	N	94.49	-0.06	86.64	0.87
**Plate B**			1646	3356			89.73	-0.59	91.33	1.04
**Avg**			1575.5	3415			92.11	-0.33	88.99	0.96
**Plate A**	NM_032154	PCGF6	1528	2176	N	N	95.75	0.07	78.68	0.36
**Plate B**			504	1014			93.25	-0.09	79.09	0.44
**Avg**			1016	1595			94.50	-0.01	78.88	0.40
**Plate A**	NM_007028	TRIM31	1520	2583	N	N	92.24	-0.30	68.45	-0.28
**Plate B**			1631	683			83.14	-1.53	57.83	-0.62
**Avg**			1575.5	1633			87.69	-0.92	63.14	-0.45
**Plate A**	XM_941875	LOC652433	1727	2727	N	N	98.20	0.32	82.84	0.63
**Plate B**			792	1502			95.45	0.22	71.57	0.06
**Avg**			1259.5	2114.5			96.83	0.27	77.20	0.34
**Plate A**	NM_172070	ZNF650	2158	2607	N	N	99.17	0.43	87.42	0.91
**Plate B**			1892	2125			98.26	0.62	82.96	0.63
**Avg**			2025	2366			98.71	0.52	85.19	0.77
**Plate A**	NM_002202	ISL1	2201	3292	N	N	95.59	0.05	41.86	-1.95
**Plate B**			2678	1296			97.61	0.53	43.06	-1.36
**Avg**			2439.5	2294			96.60	0.29	42.46	-1.65
**Plate A**	NM_020857	VPS18	1863	2158	N	N	78.80	-1.70	38.74	-2.14
**Plate B**			2175	1659			79.26	-2.08	35.99	-1.71
**Avg**			2019	1908.5			79.03	-1.89	37.36	-1.93
**Plate A**	NM_013377	PDZRN4	3080	3711	N	N	88.80	-0.66	58.18	-0.92
**Plate B**			2303	2930			80.89	-1.85	36.25	-1.70
**Avg**			2691.5	3320.5			84.85	-1.26	47.21	-1.31
**Plate A**	NM_001017981	RNF215	1020	1531	N	N	97.75	0.28	75.51	0.17
**Plate B**			849	904			91.05	-0.40	66.37	-0.20
**Avg**			934.5	1217.5			94.40	-0.06	70.94	-0.02
**Plate A**	NM_080745	TRIM69	2457	2492	N	N	98.01	0.30	87.28	0.91
**Plate B**			1896	1882			96.20	0.33	82.73	0.62
**Avg**			2176.5	2187			97.10	0.32	85.01	0.76
**Plate A**	NM_000658	AIRE	2630	3503	N	N	98.21	0.33	92.21	1.21
**Plate B**			1907	1490			96.22	0.33	69.93	-0.02
**Avg**			2268.5	2496.5			97.22	0.33	81.07	0.60
**Plate A**	NM_004562	PARK2	3526	3585	N	N	96.94	0.19	74.98	0.13
**Plate B**			1275	1415			82.98	-1.55	70.46	0.01
**Avg**			2400.5	2500			89.96	-0.68	72.72	0.07

**Plate A**	NM_001080434	LMTK3	1028	1383	N	N	89.88	-0.55	83.59	0.67
**Plate B**			1417	733			86.94	-0.99	76.67	0.32
**Avg**			1222.5	1058			88.41	-0.77	80.13	0.49
**Plate A**	XM_936301	LOC390231	2427	2557	N	N	99.18	0.43	88.50	0.98
**Plate B**			2075	1845			97.83	0.56	86.50	0.80
**Avg**			2251	2201			98.50	0.49	87.50	0.89
**Plate A**	XM_208043	LOC283116	1815	2630	N	N	97.41	0.24	89.16	1.02
**Plate B**			2037	1653			98.43	0.65	78.89	0.43
**Avg**			1926	2141.5			97.92	0.45	84.03	0.72
**Plate A**	NM_004620	TRAF6	2322	3030	N	N	77.86	-1.80	41.85	-1.95
**Plate B**			2063	2192			77.90	-2.28	34.90	-1.76
**Avg**			2192.5	2611			77.88	-2.04	38.37	-1.86
**Plate A**	NM_007212	RNF2	1260	3006	N	N	72.30	-2.38	68.76	-0.26
**Plate B**			1191	1647			72.38	-3.07	32.42	-1.89
**Avg**			1225.5	2326.5			72.34	-2.73	50.59	-1.07
**Plate A**	NM_001079867	PXMP3	1423	3003	N	N	53.55	-4.34	54.41	-1.16
**Plate B**			3347	1968			89.12	-0.68	18.14	-2.60
**Avg**			2385	2485.5			71.34	-2.51	36.28	-1.88
**Plate A**	NM_005082	TRIM25	1895	1556	N	N	99.00	0.41	76.93	0.26
**Plate B**			1185	2230			98.14	0.61	89.19	0.94
**Avg**			1540	1893			98.57	0.51	83.06	0.60
**Plate A**	NM_001166	BIRC2	1364	2332	N	N	71.41	-2.48	48.84	-1.51
**Plate B**			2205	2596			78.82	-2.15	57.47	-0.64
**Avg**			1784.5	2464			75.11	-2.31	53.16	-1.07
**Plate A**	NM_013450	BAZ2B	1683	2493	N	N	90.43	-0.49	50.50	-1.40
**Plate B**			2134	2269			88.33	-0.79	60.60	-0.48
**Avg**			1908.5	2381			89.38	-0.64	55.55	-0.94
**Plate A**	NM_018434	RNF130	2907	1978	N	N	93.40	-0.18	58.24	-0.92
**Plate B**			2003	2263			84.47	-1.34	73.97	0.18
**Avg**			2455	2120.5			88.93	-0.76	66.11	-0.37
**Plate A**	NM_001033910	TRAF5	1449	2288	N	N	96.62	0.16	74.08	0.08
**Plate B**			1967	1718			93.44	-0.06	76.37	0.30
**Avg**			1708	2003			95.03	0.05	75.23	0.19
**Plate A**	NM_198859	PRICKLE2	1500	1906	N	N	97.40	0.24	49.27	-1.48
**Plate B**			1706	1997			97.07	0.46	55.68	-0.73
**Avg**			1603	1951.5			97.23	0.35	52.47	-1.11
**Plate A**	XM_937424	LOC653978	1191	1714	N	N	99.16	0.42	95.04	1.39
**Plate B**			988	1181			98.99	0.73	90.77	1.02
**Avg**			1089.5	1447.5			99.07	0.58	92.91	1.20
**Plate A**	NM_001039111	TRIM71	3527	2989	N	N	87.78	-0.77	52.39	-1.29
**Plate B**			3791	3103			91.27	-0.37	29.58	-2.03
**Avg**			3659	3046			89.52	-0.57	40.99	-1.66
**Plate A**	NM_001099289	SH3MD4	1734	1979	N	N	82.24	-1.34	39.62	-2.09
**Plate B**			1839	2055			89.34	-0.65	58.93	-0.57
**Avg**			1786.5	2017			85.79	-1.00	49.27	-1.33
**Plate A**	NM_003316	TTC3	2570	2427	N	N	94.90	-0.02	72.68	-0.01
**Plate B**			921	1445			96.31	0.35	50.31	-1.00
**Avg**			1745.5	1936			95.61	0.16	61.50	-0.50
**Plate A**	NM_024778	LONRF3	328	1570	N	N	96.95	1.60	93.82	0.83
**Plate B**			253	448			87.75	-0.03	85.27	1.22
**Avg**			290.5	1009			92.35	0.78	89.54	1.02
**Plate A**	NM_014648	DZIP3	228	761	N	N	95.61	1.38	87.39	0.24
**Plate B**			50	231			82.00	-0.77	90.04	1.52
**Avg**			139	496			88.81	0.30	88.71	0.88
**Plate A**	NM_001039664	TNFRSF25	306	1068	N	N	82.03	-0.78	93.07	0.76
**Plate B**			213	447			90.14	0.28	80.76	0.93
**Avg**			259.5	757.5			86.08	-0.25	86.92	0.85
**Plate A**	NM_032681	SPRYD5	394	1635	N	N	93.15	0.99	92.72	0.73
**Plate B**			211	614			88.15	0.02	85.67	1.24
**Avg**			302.5	1124.5			90.65	0.50	89.19	0.99
**Plate A**	NM_174916	UBR1	164	1068	N	N	93.90	1.11	93.45	0.80
**Plate B**			135	164			86.67	-0.17	82.93	1.07
**Avg**			149.5	616			90.28	0.47	88.19	0.93
**Plate A**	NM_014727	MLL4	176	1059	N	N	93.18	0.99	87.16	0.21
**Plate B**			212	390			95.28	0.94	78.72	0.81
**Avg**			194	724.5			94.23	0.97	82.94	0.51
**Plate A**	NM_178861	RNF113B	121	721	N	N	85.12	-0.29	72.40	-1.15
**Plate B**			108	176			92.59	0.59	68.75	0.18
**Avg**			114.5	448.5			88.86	0.15	70.57	-0.49
**Plate A**	NM_002636	PHF1	414	1300	N	N	92.51	0.89	89.54	0.44
**Plate B**			314	420			88.85	0.11	78.33	0.78
**Avg**			364	860			90.68	0.50	83.94	0.61
**Plate A**	NM_020870	SH3MD2	321	1126	N	N	91.59	0.74	86.86	0.19
**Plate B**			270	705			87.04	-0.12	83.55	1.11
**Avg**			295.5	915.5			89.31	0.31	85.20	0.65

**Plate A**	NM_002931	RING1	468	1029	N	N	93.80	1.09	85.71	0.08
**Plate B**			228	586			96.93	1.15	89.08	1.46
**Avg**			348	807.5			95.37	1.12	87.40	0.77
**Plate A**	NM_031919	FSD1L	635	1401	N	N	85.67	-0.20	67.88	-1.57
**Plate B**			310	688			77.10	-1.41	43.31	-1.43
**Avg**			472.5	1044.5			81.38	-0.81	55.60	-1.50
**Plate A**	NM_000448	RAG1	335	1242	N	N	85.37	-0.25	82.21	-0.24
**Plate B**			405	463			84.94	-0.40	55.08	-0.68
**Avg**			370	852.5			85.16	-0.32	68.64	-0.46
**Plate A**	NM_004295	TRAF4	40	533	N	N	85.00	-0.31	72.80	-1.11
**Plate B**			80	157			81.25	-0.87	61.15	-0.30
**Avg**			60	345			83.13	-0.59	66.97	-0.71
**Plate A**	NM_006568	CGRRF1	309	740	N	N	91.59	0.74	68.51	-1.51
**Plate B**			118	399			88.98	0.13	54.64	-0.71
**Avg**			213.5	569.5			90.28	0.43	61.58	-1.11
**Plate A**	NM_018133	MSL2L1	978	1875	N	N	84.66	-0.36	51.15	-3.12
**Plate B**			410	845			81.95	-0.78	41.18	-1.56
**Avg**			694	1360			83.31	-0.57	46.17	-2.34
**Plate A**	NM_016271	RNF138	501	1533	N	N	86.83	-0.02	86.50	0.15
**Plate B**			230	938			88.70	0.09	76.33	0.66
**Avg**			365.5	1235.5			87.76	0.04	81.41	0.40
**Plate A**	NM_001656	TRIM23	627	1411	N	N	94.42	1.19	46.21	-3.57
**Plate B**			438	873			87.44	-0.07	42.96	-1.45
**Avg**			532.5	1142			90.93	0.56	44.58	-2.51
**Plate A**	NM_014868	RNF10	669	1291	N	N	89.39	0.39	71.03	-1.28
**Plate B**			415	1165			89.64	0.21	76.14	0.64
**Avg**			542	1228			89.51	0.30	73.58	-0.32
**Plate A**	NM_012074	DPF3	1028	1528	N	N	94.65	1.23	76.64	-0.76
**Plate B**			616	1665			93.02	0.65	65.83	-0.01
**Avg**			822	1596.5			93.83	0.94	71.23	-0.38
**Plate A**	NM_172071	RC3H1	517	1300	N	N	90.33	0.54	74.92	-0.92
**Plate B**			480	645			87.29	-0.09	74.88	0.56
**Avg**			498.5	972.5			88.81	0.22	74.90	-0.18
**Plate A**	NM_024539	RNF128	385	862	N	N	87.01	0.01	78.77	-0.56
**Plate B**			204	335			88.73	0.09	58.81	-0.45
**Avg**			294.5	598.5			87.87	0.05	68.79	-0.51
**Plate A**	NM_022455	NSD1	596	1560	N	N	82.72	-0.67	90.00	0.48
**Plate B**			1475	877			92.95	0.64	62.94	-0.19
**Avg**			1035.5	1218.5			87.83	-0.02	76.47	0.14
**Plate A**	NM_194359	RNF41	473	1725	N	N	75.48	-1.83	62.20	-2.09
**Plate B**			338	657			72.19	-2.04	40.33	-1.61
**Avg**			405.5	1191			73.83	-1.94	51.27	-1.85
**Plate A**	NM_001100875	RNF190	211	1397	N	N	83.89	-0.49	62.13	-2.10
**Plate B**			114	215			87.72	-0.04	31.63	-2.16
**Avg**			162.5	806			85.80	-0.26	46.88	-2.13
**Plate A**	NM_014593	CXXC1	305	1672	N	N	83.28	-0.58	90.43	0.52
**Plate B**			183	403			90.16	0.28	86.35	1.29
**Avg**			244	1037.5			86.72	-0.15	88.39	0.90
**Plate A**	XM_115100	LOC196346	888	2129	N	N	86.94	0.00	69.19	-1.45
**Plate B**			683	1454			82.72	-0.68	54.95	-0.69
**Avg**			785.5	1791.5			84.83	-0.34	62.07	-1.07
**Plate A**	NM_005762	TRIM28	598	1570	N	N	81.77	-0.82	63.18	-2.00
**Plate B**			615	521			89.11	0.14	57.97	-0.50
**Avg**			606.5	1045.5			85.44	-0.34	60.58	-1.25
**Plate A**	NM_016578	HBXAP	548	1374	N	N	89.05	0.34	82.17	-0.25
**Plate B**			144	449			83.33	-0.60	73.94	0.50
**Avg**			346	911.5			86.19	-0.13	78.06	0.13
**Plate A**	NM_058166	TRIM6	741	2566	N	N	90.55	0.58	81.68	-0.29
**Plate B**			536	1301			86.94	-0.14	63.03	-0.18
**Avg**			638.5	1933.5			88.75	0.22	72.36	-0.24
**Plate A**	XM_941879	LOC652436	712	1550	N	N	90.87	0.63	78.45	-0.59
**Plate B**			475	1088			89.68	0.22	76.10	0.64
**Avg**			593.5	1319			90.28	0.42	77.28	0.03
**Plate A**	NM_001002245	ANAPC11	638	1082	N	N	83.70	-0.52	74.58	-0.95
**Plate B**			221	360			76.47	-1.49	46.67	-1.21
**Avg**			429.5	721			80.08	-1.00	60.63	-1.08
**Plate A**	NM_138396	MARCH9	635	1171	N	N	82.68	-0.68	81.90	-0.27
**Plate B**			235	678			79.57	-1.09	67.85	0.12
**Avg**			435	924.5			81.13	-0.88	74.87	-0.08
**Plate A**	NM_014160	MKRN2	668	1466	N	N	72.90	-2.24	68.14	-1.54
**Plate B**			485	791			71.96	-2.07	36.28	-1.87
**Avg**			576.5	1128.5			72.43	-2.16	52.21	-1.71
**Plate A**	NM_016125	LOC51136	647	1683	N	N	80.37	-1.05	85.09	0.02
**Plate B**			533	879			80.30	-0.99	63.94	-0.13
**Avg**			590	1281			80.34	-1.02	74.51	-0.05

Plate A	NM_001008274	TRIM72	408	1485	N	N	88.97	0.32	82.15	-0.25
Plate B			158	488			91.77	0.49	55.74	-0.64
Avg			283	986.5			90.37	0.41	68.95	-0.45
Plate A	NM_207111	TRIAD3	843	2112	N	N	75.09	-1.89	80.78	-0.38
Plate B			436	876			83.03	-0.64	60.50	-0.34
Avg			639.5	1494			79.06	-1.27	70.64	-0.36
Plate A	NM_014817	KIAA0644	968	2146	N	N	87.19	0.04	84.86	0.00
Plate B			648	1067			88.58	0.08	66.64	0.04
Avg			808	1606.5			87.89	0.06	75.75	0.02
Plate A	XM_942563	LOC652859	431	2127	N	N	81.90	-0.80	90.08	0.49
Plate B			276	647			77.90	-1.30	64.45	-0.09
Avg			353.5	1387			79.90	-1.05	77.27	0.20
Plate A	NM_015565	ZNF294	588	1549	N	N	93.71	1.08	86.31	0.14
Plate B			150	900			93.33	0.69	77.89	0.75
Avg			369	1224.5			93.52	0.88	82.10	0.45
Plate A	NM_144726	FLJ31951	839	815	N	N	83.79	-0.50	82.33	-0.23
Plate B			474	1086			79.96	-1.04	84.25	1.15
Avg			656.5	950.5			81.87	-0.77	83.29	0.46
Plate A	NM_001009991	SYTL3	483	704	N	N	89.65	0.43	86.22	0.13
Plate B			101	252			87.13	-0.11	49.60	-1.03
Avg			292	478			88.39	0.16	67.91	-0.45
Plate A	NM_004653	JARID1D	433	1064	N	N	85.91	-0.16	96.24	1.06
Plate B			293	363			95.56	0.98	79.89	0.88
Avg			363	713.5			90.74	0.41	88.07	0.97
Plate A	XM_926437	LOC653192	491	1557	N	N	75.97	-1.75	81.89	-0.27
Plate B			231	410			82.68	-0.69	46.10	-1.25
Avg			361	983.5			79.33	-1.22	63.99	-0.76
Plate A	NM_025058	TRIM46	626	1591	N	N	91.85	0.78	84.85	0.00
Plate B			271	609			94.83	0.88	34.48	-1.98
Avg			448.5	1100			93.34	0.83	59.67	-0.99
Plate A	NM_015153	PHF3	505	1955	N	N	80.59	-1.01	84.91	0.01
Plate B			156	784			85.90	-0.27	61.86	-0.26
Avg			330.5	1369.5			83.25	-0.64	73.39	-0.12
Plate A	NM_021026	RFPL1	219	1098	N	N	89.95	0.48	94.72	0.91
Plate B			43	242			95.35	0.95	86.36	1.29
Avg			131	670			92.65	0.72	90.54	1.10
Plate A	NM_145051	RNF183	616	1630	N	N	85.06	-0.30	86.26	0.13
Plate B			272	1166			91.18	0.41	66.04	0.01
Avg			444	1398			88.12	0.06	76.15	0.07
Plate A	NM_182521	ZSWIM2	432	1409	N	N	84.03	-0.46	86.30	0.14
Plate B			266	338			93.98	0.77	71.30	0.34
Avg			349	873.5			89.01	0.15	78.80	0.24
Plate A	NM_006007	ZNF216	794	1203	N	N	93.45	1.04	90.27	0.50
Plate B			681	928			93.54	0.72	83.41	1.10
Avg			737.5	1065.5			93.49	0.88	86.84	0.80
Plate A	NM_001009936	PHF19	969	1899	N	N	94.63	1.23	90.78	0.55
Plate B			417	1581			94.96	0.90	82.04	1.01
Avg			693	1740			94.80	1.06	86.41	0.78
Plate A	NM_002597	PDC	437	1304	N	N	83.98	-0.47	88.04	0.30
Plate B			176	528			81.25	-0.87	40.53	-1.60
Avg			306.5	916			82.62	-0.67	64.28	-0.65
Plate A	NM_004416	DTX1	476	1642	N	N	77.31	-1.54	94.46	0.89
Plate B			183	590			83.06	-0.64	73.73	0.49
Avg			329.5	1116			80.19	-1.09	84.09	0.69
Plate A	NM_018288	PHF10	390	1051	N	N	77.18	-1.56	87.92	0.29
Plate B			162	290			72.84	-1.96	52.07	-0.87
Avg			276	670.5			75.01	-1.76	69.99	-0.29
Plate A	NM_025188	TRIM45	612	1414	N	N	86.27	-0.11	87.77	0.27
Plate B			458	771			87.99	0.00	60.57	-0.34
Avg			535	1092.5			87.13	-0.05	74.17	-0.03
Plate A	NM_001099679	TRIM32	460	1151	N	N	85.65	-0.21	83.32	-0.14
Plate B			96	399			84.38	-0.47	65.66	-0.02
Avg			278	775			85.01	-0.34	74.49	-0.08
Plate A	NM_194452	RNF121	525	768	N	N	89.90	0.47	95.83	1.02
Plate B			249	520			91.57	0.46	87.88	1.38
Avg			387	644			90.74	0.47	91.86	1.20
Plate A	NM_032015	RNF26	343	1258	N	N	88.63	0.27	92.37	0.70
Plate B			120	259			90.83	0.37	79.15	0.83
Avg			231.5	758.5			89.73	0.32	85.76	0.76
Plate A	NM_005553	KRTAP5	245	898	N	N	61.22	-4.10	93.32	0.78
Plate B			75	131			48.00	-5.17	75.57	0.61
Avg			160	514.5			54.61	-4.63	84.45	0.70
Plate A	NM_183384	RNF13	782	2416	N	N	81.97	-0.79	83.49	-0.12
Plate B			333	518			79.28	-1.13	35.91	-1.89
Avg			557.5	1467			80.62	-0.96	59.70	-1.01

**Plate A**	NM_153341	IBRDC3	450	995	N	N	95.33	1.34	83.92	-0.08
**Plate B**			154	815			94.81	0.88	66.50	0.04
**Avg**			302	905			95.07	1.11	75.21	-0.02
**Plate A**	NM_015303	KIAA0804	437	998	N	N	81.24	-0.91	86.27	0.13
**Plate B**			271	439			81.18	-0.88	44.87	-1.33
**Avg**			354	718.5			81.21	-0.90	65.57	-0.60
**Plate A**	NM_020358	TRIM49	544	1261	N	N	86.95	0.00	78.27	-0.61
**Plate B**			254	641			92.52	0.58	41.65	-1.53
**Avg**			399	951			89.73	0.29	59.96	-1.07
**Plate A**	NM_080737	SYTL4	405	733	N	N	78.52	-1.34	91.54	0.62
**Plate B**			331	428			82.48	-0.71	71.96	0.38
**Avg**			368	580.5			80.50	-1.03	81.75	0.50
**Plate A**	NM_002938	RNF4	597	1857	N	N	84.59	-0.38	93.38	0.79
**Plate B**			615	357			91.06	0.40	76.19	0.65
**Avg**			606	1107			87.82	0.01	84.78	0.72
**Plate A**	NM_005358	LMO7	579	1501	N	N	84.11	-0.45	85.54	0.07
**Plate B**			530	504			93.96	0.77	66.07	0.01
**Avg**			554.5	1002.5			89.04	0.16	75.81	0.04
**Plate A**	NM_018223	CHFR	788	1325	N	N	85.91	-0.16	45.13	-3.67
**Plate B**			337	644			86.05	-0.25	26.09	-2.51
**Avg**			562.5	984.5			85.98	-0.21	35.61	-3.09
**Plate A**	NM_002431	MNAT1	945	1280	N	N	92.49	0.88	87.50	0.25
**Plate B**			246	645			86.18	-0.23	58.45	-0.47
**Avg**			595.5	962.5			89.33	0.32	72.97	-0.11
**Plate A**	XM_929704	LOC646754	807	1341	N	N	87.36	0.07	64.95	-1.84
**Plate B**			465	866			76.56	-1.48	45.38	-1.29
**Avg**			636	1103.5			81.96	-0.71	55.17	-1.57
**Plate A**	NM_007144	PCGF2	430	1296	N	N	90.70	0.60	68.60	-1.50
**Plate B**			500	623			92.40	0.57	50.40	-0.98
**Avg**			465	959.5			91.55	0.58	59.50	-1.24
**Plate A**	NM_024544	C1orf166	909	1690	N	N	85.92	-0.16	77.99	-0.63
**Plate B**			435	1375			78.62	-1.21	70.18	0.27
**Avg**			672	1532.5			82.27	-0.69	74.08	-0.18
**Plate A**	NM_173084	TRIM59	32	197	Y	N	96.88	1.58	78.68	-0.57
**Plate B**			28	30			78.57	-1.22	83.33	1.10
**Avg**			30	113.5			87.72	0.18	81.01	0.26
**Plate A**	NM_005067	SIAH2	19	116	Y	N	84.21	-0.44	83.62	-0.11
**Plate B**			21	42			71.43	-2.14	90.48	1.55
**Avg**			20	79			77.82	-1.29	87.05	0.72
**Plate A**	NM_017763	RNF43	76	506	N	N	89.47	0.40	78.85	-0.55
**Plate B**			114	93			92.98	0.64	63.44	-0.16
**Avg**			95	299.5			91.23	0.52	71.15	-0.36
**Plate A**	NM_152553	IBRDC1	401	909	N	N	89.78	0.45	87.46	0.24
**Plate B**			232	294			90.52	0.33	74.49	0.54
**Avg**			316.5	601.5			90.15	0.39	80.97	0.39
**Plate A**	NM_019062	RNF186	452	1042	N	N	83.63	-0.53	74.66	-0.94
**Plate B**			427	462			92.74	0.61	53.25	-0.80
**Avg**			439.5	752			88.18	0.04	63.96	-0.87
**Plate A**	NM_022826	MARCH7	442	928	N	N	90.05	0.49	89.76	0.46
**Plate B**			240	508			94.58	0.85	66.93	0.06
**Avg**			341	718			92.31	0.67	78.35	0.26
**Plate A**	NM_020205	ZA20D1	361	830	N	N	88.64	0.27	84.82	0.00
**Plate B**			256	395			92.97	0.64	69.87	0.25
**Avg**			308.5	612.5			90.81	0.46	77.35	0.12
**Plate A**	NM_187841	TRIM54	205	431	N	N	97.07	1.62	87.70	0.27
**Plate B**			59	236			96.61	1.11	59.75	-0.39
**Avg**			132	333.5			96.84	1.36	73.72	-0.06
**Plate A**	NM_032431	SYVN1	751	551	N	N	92.41	0.87	70.42	-1.33
**Plate B**			175	630			88.00	0.00	56.51	-0.59
**Avg**			463	590.5			90.21	0.44	63.46	-0.96
**Plate A**	NM_032246	RKHD3	349	535	N	N	94.84	1.26	79.81	-0.46
**Plate B**			179	515			94.97	0.90	65.24	-0.04
**Avg**			264	525			94.91	1.08	72.53	-0.25
**Plate A**	XM_062300	LOC120824	372	745	N	N	93.01	1.37	85.10	1.71
**Plate B**			149	354			85.91	-0.19	75.71	0.34
**Avg**			260.5	549.5			89.46	0.59	80.40	1.03
**Plate A**	NM_183399	RNF14	255	552	N	N	89.80	0.66	61.41	-1.84
**Plate B**			187	382			93.05	1.27	69.63	-0.42
**Avg**			221	467			91.43	0.96	65.52	-1.13
**Plate A**	NM_004811	LPXN	412	740	N	N	90.05	0.72	64.86	-1.33
**Plate B**			215	625			85.12	-0.35	83.52	1.32
**Avg**			313.5	682.5			87.58	0.18	74.19	0.00
**Plate A**	NM_014372	RNF11	388	1009	N	N	93.30	1.43	69.77	-0.59
**Plate B**			443	522			93.68	1.40	79.69	0.84
**Avg**			415.5	765.5			93.49	1.41	74.73	0.13

**Plate A**	NM_005798	RFP2	240	887	N	N	90.83	0.89	70.69	-0.45
**Plate B**			239	450			90.38	0.72	83.11	1.27
**Avg**			239.5	668.5			90.60	0.81	76.90	0.41
**Plate A**	NM_015431	TRIM58	418	629	N	N	93.54	1.49	72.66	-0.16
**Plate B**			192	498			88.54	0.35	87.55	1.83
**Avg**			305	563.5			91.04	0.92	80.10	0.83
**Plate A**	NM_016561	BFAR	506	959	N	N	91.11	0.95	75.60	0.28
**Plate B**			188	484			84.04	-0.57	76.45	0.44
**Avg**			347	721.5			87.57	0.19	76.02	0.36
**Plate A**	NM_015435	RNF19	461	847	N	N	90.24	0.76	84.18	1.57
**Plate B**			78	711			83.33	-0.71	90.58	2.21
**Avg**			269.5	779			86.79	0.02	87.38	1.89
**Plate A**	NM_004634	BRPF1	338	1465	N	N	84.91	-0.42	83.96	1.54
**Plate B**			158	475			93.67	1.39	78.95	0.75
**Avg**			248	970			89.29	0.49	81.45	1.14
**Plate A**	NM_194460	RNF126	426	777	N	N	91.78	1.10	80.18	0.97
**Plate B**			205	767			95.61	1.79	90.35	2.18
**Avg**			315.5	772			93.70	1.44	85.27	1.57
**Plate A**	NM_006355	TRIM38	520	769	N	N	89.04	0.49	82.57	1.33
**Plate B**			276	645			89.86	0.62	73.33	0.05
**Avg**			398	707			89.45	0.55	77.95	0.69
**Plate A**	NM_001098638	RNF169	340	1157	N	N	86.76	-0.01	64.30	-1.41
**Plate B**			300	529			83.67	-0.65	72.21	-0.10
**Avg**			320	843			85.22	-0.33	68.26	-0.75
**Plate A**	NM_024814	CBLL1	460	1075	N	N	78.26	-1.88	68.09	-0.84
**Plate B**			236	568			83.90	-0.60	72.89	-0.01
**Avg**			348	821.5			81.08	-1.24	70.49	-0.43
**Plate A**	NM_032622	LNX1	632	1052	N	N	84.18	-0.58	71.29	-0.36
**Plate B**			350	1144			89.43	0.53	75.70	0.34
**Avg**			491	1098			86.80	-0.02	73.50	-0.01
**Plate A**	NM_005744	ARIH1	412	938	N	N	84.95	-0.41	65.78	-1.19
**Plate B**			276	685			92.03	1.06	68.61	-0.55
**Avg**			344	811.5			88.49	0.33	67.20	-0.87
**Plate A**	NM_173662	RNF175	725	1118	N	N	87.72	0.20	67.35	-0.95
**Plate B**			225	804			91.11	0.87	77.11	0.52
**Avg**			475	961			89.42	0.54	72.23	-0.22
**Plate A**	NM_004210	NEURL	614	1668	N	N	86.48	-0.07	79.50	0.87
**Plate B**			447	999			89.93	0.63	72.67	-0.04
**Avg**			530.5	1333.5			88.21	0.28	76.08	0.42
**Plate A**	NM_153371	LNX2	684	1304	N	N	87.87	0.23	76.69	0.45
**Plate B**			622	870			90.68	0.78	74.83	0.23
**Avg**			653	1087			89.27	0.51	75.76	0.34
**Plate A**	NM_031277	RNF17	319	1210	N	N	78.37	-1.86	75.62	0.29
**Plate B**			321	1175			88.79	0.40	84.94	1.50
**Avg**			320	1192.5			83.58	-0.73	80.28	0.89
**Plate A**	NM_007219	RNF24	613	876	N	N	88.58	0.39	76.48	0.42
**Plate B**			438	938			89.04	0.45	69.40	-0.45
**Avg**			525.5	907			88.81	0.42	72.94	-0.02
**Plate A**	NM_030963	RNF146	536	1189	N	N	91.98	1.14	79.14	0.81
**Plate B**			217	397			88.02	0.24	66.25	-0.84
**Avg**			376.5	793			90.00	0.69	72.69	-0.01
**Plate A**	NM_001017397	TRIM36	810	1022	N	N	86.17	-0.14	71.53	-0.33
**Plate B**			417	846			86.33	-0.10	58.51	-1.81
**Avg**			613.5	934			86.25	-0.12	65.02	-1.07
**Plate A**	NM_005392	PHF2	563	1388	N	N	84.19	-0.58	67.07	-0.99
**Plate B**			346	858			82.66	-0.85	68.65	-0.54
**Avg**			454.5	1123			83.43	-0.71	67.86	-0.77
**Plate A**	NM_002617	PEX10	696	1299	N	N	88.36	0.34	63.43	-1.54
**Plate B**			476	884			92.65	1.19	69.12	-0.48
**Avg**			586	1091.5			90.50	0.76	66.28	-1.01
**Plate A**	NM_013262	MYLIP	446	1210	N	N	74.89	-2.63	57.93	-2.36
**Plate B**			503	808			89.07	0.45	62.62	-1.30
**Avg**			474.5	1009			81.98	-1.09	60.28	-1.83
**Plate A**	NM_032268	ZNRF1	693	815	N	N	92.21	1.19	67.48	-0.93
**Plate B**			135	551			78.52	-1.70	52.09	-2.62
**Avg**			414	683			85.36	-0.25	59.79	-1.78
**Plate A**	NM_145214	TRIM11	433	1461	N	N	83.14	-0.81	73.51	-0.03
**Plate B**			373	945			83.11	-0.76	65.29	-0.96
**Avg**			403	1203			83.13	-0.78	69.40	-0.50
**Plate A**	NM_033034	TRIM5	889	929	N	N	83.35	-0.76	68.03	-0.85
**Plate B**			510	879			88.04	0.25	63.48	-1.19
**Avg**			699.5	904			85.70	-0.26	65.76	-1.02
**Plate A**	NM_032271	TRAF7	824	1057	N	N	91.26	0.98	68.40	-0.80
**Plate B**			366	998			87.98	0.23	68.14	-0.61
**Avg**			595	1027.5			89.62	0.61	68.27	-0.70

Plate A	NM_015107	PHF8	919	1220	N	N	91.73	1.09	71.07	-0.40
Plate B			534	992			92.88	1.23	65.02	-1.00
Avg			726.5	1106			92.31	1.16	68.04	-0.70
Plate A	NM_030954	RNF170	539	848	N	N	85.53	-0.28	83.37	1.45
Plate B			222	586			77.48	-1.91	67.58	-0.68
Avg			380.5	717			81.50	-1.09	75.47	0.39
Plate A	NM_152737	RNF182	448	1069	N	N	79.91	-1.52	62.39	-1.70
Plate B			316	918			85.76	-0.22	75.71	0.34
Avg			382	993.5			82.84	-0.87	69.05	-0.68
Plate A	NM_001048201	UHRF1	605	999	N	N	80.17	-1.46	67.77	-0.89
Plate B			360	756			84.72	-0.43	73.81	0.11
Avg			482.5	877.5			82.44	-0.95	70.79	-0.39
Plate A	NM_207343	DKFZP547C195	621	1277	N	N	89.37	0.57	70.16	-0.53
Plate B			587	652			92.84	1.23	66.56	-0.80
Avg			604	964.5			91.11	0.90	68.36	-0.67
Plate A	NM_014819	PJA2	678	1417	N	N	84.37	-0.54	73.18	-0.08
Plate B			339	638			88.79	0.40	67.71	-0.66
Avg			508.5	1027.5			86.58	-0.07	70.45	-0.37
Plate A	NM_033247	PML	816	1314	N	N	89.58	0.61	84.09	1.56
Plate B			630	642			85.71	-0.23	73.52	0.07
Avg			723	978			87.65	0.19	78.81	0.81
Plate A	NM_199415	UBOX5	947	1236	N	N	83.21	-0.79	67.88	-0.87
Plate B			443	906			80.81	-1.23	67.22	-0.72
Avg			695	1071			82.01	-1.01	67.55	-0.80
Plate A	NM_173547	TRIM65	746	1202	N	N	80.03	-1.49	70.55	-0.47
Plate B			378	943			87.83	0.20	81.23	1.04
Avg			562	1072.5			83.93	-0.64	75.89	0.28
Plate A	NM_006268	DPF2	1045	1239	N	N	89.00	0.48	65.21	-1.27
Plate B			433	1034			86.14	-0.14	75.24	0.28
Avg			739	1136.5			87.57	0.17	70.23	-0.49
Plate A	NM_004187	JARID1C	873	1217	N	N	92.78	1.32	74.20	0.07
Plate B			440	1104			89.55	0.55	77.81	0.61
Avg			656.5	1160.5			91.16	0.94	76.00	0.34
Plate A	NM_032408	BAZ1B	618	919	N	N	92.88	1.34	74.97	0.19
Plate B			311	460			87.46	0.13	64.78	-1.03
Avg			464.5	689.5			90.17	0.73	69.88	-0.42
Plate A	NM_153042	AOF1	390	949	N	N	81.03	-1.27	64.59	-1.37
Plate B			304	555			77.96	-1.81	69.01	-0.50
Avg			347	752			79.49	-1.54	66.80	-0.93
Plate A	NM_006978	RNF113A	912	1156	N	N	89.25	0.54	77.42	0.56
Plate B			638	772			96.71	2.01	75.26	0.29
Avg			775	964			92.98	1.28	76.34	0.42
Plate A	NM_032373	PCGF5	1082	1363	N	N	84.38	-0.53	65.74	-1.19
Plate B			351	1121			88.03	0.24	73.68	0.09
Avg			716.5	1242			86.21	-0.14	69.71	-0.55
Plate A	NM_002393	MDM4	580	1247	N	N	88.45	0.36	67.84	-0.88
Plate B			266	390			84.59	-0.46	64.36	-1.08
Avg			423	818.5			86.52	-0.05	66.10	-0.98
Plate A	NM_003141	SSA1	434	1191	N	N	82.95	-0.85	76.15	0.37
Plate B			395	890			79.49	-1.50	72.02	-0.12
Avg			414.5	1040.5			81.22	-1.17	74.09	0.12
Plate A	NM_017769	KIAA1333	714	1144	N	N	85.15	-0.36	75.09	0.21
Plate B			771	592			86.12	-0.15	58.11	-1.86
Avg			742.5	868			85.64	-0.25	66.60	-0.83
Plate A	XM_934453	LOC399940	1040	1269	N	N	85.19	-0.35	65.41	-1.24
Plate B			412	887			83.74	-0.63	81.17	1.03
Avg			726	1078			84.47	-0.49	73.29	-0.11
Plate A	NM_178125	TRIM50A	528	946	N	N	80.30	-1.43	68.39	-0.80
Plate B			366	905			88.25	0.29	80.55	0.95
Avg			447	925.5			84.28	-0.57	74.47	0.08
Plate A	NM_000243	MEFV	386	1664	N	N	82.38	-0.97	73.44	-0.04
Plate B			250	1070			90.80	0.81	82.80	1.23
Avg			318	1367			86.59	-0.08	78.12	0.60
Plate A	NM_006910	RBBP6	460	765	N	N	86.09	-0.16	70.85	-0.43
Plate B			193	485			86.01	-0.17	71.96	-0.13
Avg			326.5	625			86.05	-0.16	71.40	-0.28
Plate A	NM_001080419	UNK	657	1081	N	N	89.65	0.63	71.51	-0.33
Plate B			308	543			87.34	0.10	70.72	-0.28
Avg			482.5	812			88.49	0.37	71.11	-0.31
Plate A	NM_017610	RNF111	697	927	N	N	87.66	0.19	65.16	-1.28
Plate B			475	650			80.42	-1.31	58.62	-1.80
Avg			586	788.5			84.04	-0.56	61.89	-1.54
Plate A	NM_018073	TRIM68	553	1186	N	N	77.58	-2.03	68.13	-0.84
Plate B			660	523			89.55	0.55	52.20	-2.60
Avg			606.5	854.5			83.56	-0.74	60.16	-1.72

**Plate A**	NM_033452	TRIM47	442	1106	N	N	77.38	-2.08	73.24	-0.07
**Plate B**			286	999			84.62	-0.45	67.47	-0.69
**Avg**			364	1052.5			81.00	-1.26	70.35	-0.38
**Plate A**	NM_001167	BIRC4	870	1355	N	N	87.24	0.10	80.52	1.02
**Plate B**			607	809			84.68	-0.44	69.22	-0.47
**Avg**			738.5	1082			85.96	-0.17	74.87	0.28
**Plate A**	NM_031297	RNF208	571	872	N	N	89.67	0.63	75.80	0.31
**Plate B**			179	717			73.74	-2.67	75.31	0.29
**Avg**			375	794.5			81.71	-1.02	75.56	0.30
**Plate A**	XM_928029	RNF187	905	1569	N	N	80.00	-1.50	80.43	1.01
**Plate B**			430	946			84.88	-0.40	81.61	1.08
**Avg**			667.5	1257.5			82.44	-0.95	81.02	1.05
**Plate A**	NM_198586	NHLRC1	837	1600	N	N	87.81	0.22	76.88	0.47
**Plate B**			528	1267			87.50	0.14	80.74	0.97
**Avg**			682.5	1433.5			87.66	0.18	78.81	0.72
**Plate A**	NM_033341	BIRC8	501	1146	N	N	88.82	0.45	78.27	0.68
**Plate B**			512	828			92.97	1.25	68.96	-0.50
**Avg**			506.5	987			90.90	0.85	73.62	0.09
**Plate A**	NM_024114	TRIM48	635	866	N	N	84.57	-0.49	75.52	0.27
**Plate B**			167	325			84.43	-0.49	62.77	-1.28
**Avg**			401	595.5			84.50	-0.49	69.14	-0.50
**Plate A**	NM_017923	MARCH1	643	930	N	N	77.14	-2.13	75.70	0.30
**Plate B**			185	809			78.92	-1.61	73.05	0.01
**Avg**			414	869.5			78.03	-1.87	74.38	0.15
**Plate A**	NM_001009569	MLLT10	637	735	N	N	81.32	-1.21	72.79	-0.14
**Plate B**			544	698			78.49	-1.70	61.17	-1.48
**Avg**			590.5	716.5			79.91	-1.45	66.98	-0.81
**Plate A**	NM_014455	ZNF364	520	1096	N	N	85.58	-0.27	73.91	0.03
**Plate B**			374	504			85.56	-0.26	64.88	-1.01
**Avg**			447	800			85.57	-0.26	69.39	-0.49
**Plate A**	NM_013446	MKRN1	576	852	N	N	85.94	-0.19	74.88	0.18
**Plate B**			349	735			79.66	-1.46	66.26	-0.84
**Avg**			462.5	793.5			82.80	-0.83	70.57	-0.33
**Plate A**	NM_032814	FLJ14627	661	1060	N	N	86.84	0.01	76.23	0.38
**Plate B**			119	682			72.27	-2.97	63.05	-1.24
**Avg**			390	871			79.55	-1.48	69.64	-0.43
**Plate A**	NM_207396	RNF207	476	945	N	N	84.24	-0.56	79.79	0.91
**Plate B**			381	723			85.04	-0.37	65.28	-0.96
**Avg**			428.5	834			84.64	-0.46	72.54	-0.03
**Plate A**	NM_178450	MARCH3	478	1361	N	N	80.54	-1.38	88.10	2.16
**Plate B**			415	1147			85.54	-0.26	79.60	0.83
**Avg**			446.5	1254			83.04	-0.82	83.85	1.49
**Plate A**	NM_198853	TRIM74	907	1157	N	N	86.66	-0.03	77.53	0.57
**Plate B**			366	1368			81.69	-1.05	78.58	0.70
**Avg**			636.5	1262.5			84.18	-0.54	78.05	0.64
**Plate A**	NM_152470	RNF165	662	765	N	N	89.58	0.61	86.14	1.86
**Plate B**			348	1134			89.94	0.63	73.28	0.04
**Avg**			505	949.5			89.76	0.62	79.71	0.95
**Plate A**	NM_052828	TRIM10	208	221	N	N	90.38	0.79	79.19	0.82
**Plate B**			55	182			92.73	1.20	78.02	0.63
**Avg**			131.5	201.5			91.56	1.00	78.60	0.73
**Plate A**	NM_003566	EEA1	273	254	N	N	91.94	1.13	76.77	0.46
**Plate B**			74	256			90.54	0.76	75.78	0.35
**Avg**			173.5	255			91.24	0.94	76.28	0.41
**Plate A**	NM_006510	RFP	324	455	N	N	88.58	0.39	83.30	1.44
**Plate B**			176	281			93.75	1.41	77.22	0.53
**Avg**			250	368			91.17	0.90	80.26	0.99
**Plate A**	NM_020914	C17ORF27	315	574	N	N	93.33	1.44	82.40	1.30
**Plate B**			225	237			87.56	0.15	73.42	0.06
**Avg**			270	405.5			90.44	0.79	77.91	0.68
**Plate A**	NM_001012414	TRIM61	450	392	N	N	87.78	0.22	80.61	1.03
**Plate B**			292	542			85.96	-0.18	77.49	0.57
**Avg**			371	467			86.87	0.02	79.05	0.80
**Plate A**	NM_012101	TRIM29	438	718	N	N	85.62	-0.26	82.03	1.25
**Plate B**			279	611			83.87	-0.60	79.71	0.84
**Avg**			358.5	664.5			84.74	-0.43	80.87	1.05
