# Supplementary material for: A High-Content RNAi Screen Identifies Ubiquitin Modifiers That Regulate TNF-Dependent Nuclear Accumulation of NF-κB
Source: Front Immunol. 2014 Jul 14;5:322. doi: 10.3389/fimmu.2014.00322 (PMC4094887; doi:10.3389/fimmu.2014.00322)
Supplement: Supplementary file 1 [file Presentation_1.ZIP › Supp. Table 4.PDF]

	Gene ID	Gene	30 mins # of cells	120 mins # of cells	Lethal	Nuclear ph	30 mins %	30 mins Z-	120 mins %	120 mins Z
Plate A	NM_004656	BAP1	270	136	N	N	88.15	0.92	70.59	1.89
Plate B			34	83			88.24	0.66	77.11	2.12
Avg			152	109.5			88.19	0.79	73.85	2.00
Plate A	NM_006837	COPS5	157	114	N	N	90.45	1.02	67.54	1.74
Plate B			149	159			97.99	1.05	66.04	1.63
Avg			153	136.5			94.22	1.03	66.79	1.68
Plate A	NM_024332	CXORF53	638	727	N	N	83.54	0.73	33.15	0.08
Plate B			219	313			78.54	0.28	40.89	0.51
Avg			428.5	520			81.04	0.51	37.02	0.30
Plate A	NM_001042355	CYLD	261	369	N	N	85.06	0.79	31.17	-0.01
Plate B			137	174			78.83	0.29	52.30	1.02
Avg			199	271.5			81.94	0.54	41.73	0.50
Plate A	XM_939510	DUB1A	258	488	N	N	86.82	0.87	47.13	0.76
Plate B			187	204			87.70	0.64	36.76	0.33
Avg			222.5	346			87.26	0.76	41.95	0.54
Plate A	NM_201402	DUB3	186	129	N	N	97.31	1.31	77.52	2.22
Plate B			168	174			96.43	0.98	70.69	1.83
Avg			177	151.5			96.87	1.15	74.10	2.03
Plate A	NM_001033024	FBXO7	366	299	N	N	69.95	0.16	45.48	0.68
Plate B			321	331			75.70	0.17	29.61	0.01
Avg			343.5	315			72.82	0.16	37.55	0.34
Plate A	NM_012180	FBXO8	330	386	N	N	95.45	1.23	73.32	2.02
Plate B			235	153			94.04	0.89	65.36	1.60
Avg			282.5	269.5			94.75	1.06	69.34	1.81
Plate A	NM_032868	FLJ14981	507	536	N	N	72.58	0.27	18.28	-0.64
Plate B			448	398			54.91	-0.64	15.33	-0.62
Avg			477.5	467			63.75	-0.19	16.81	-0.63
Plate A	NM_014876	JOSD1	486	712	N	N	89.92	1.00	46.63	0.73
Plate B			471	148			87.47	0.63	50.68	0.94
Avg			478.5	430			88.70	0.82	48.65	0.84
Plate A	NM_030660	MJD	523	757	N	N	66.35	0.00	18.49	-0.63
Plate B			152	213			84.21	0.51	33.80	0.20
Avg			337.5	485			75.28	0.25	26.15	-0.22
Plate A	NM_001085487	MYSM1	545	672	N	N	42.94	-0.98	6.40	-1.21
Plate B			293	412			34.47	-1.45	6.55	-1.01
Avg			419	542			38.70	-1.22	6.48	-1.11
Plate A	NM_017670	OTUB1	268	449	N	N	29.48	-1.55	17.59	-0.67
Plate B			125	244			36.00	-1.39	22.54	-0.30
Avg			196.5	346.5			32.74	-1.47	20.07	-0.49
Plate A	NM_023112	OTUB2	195	489	N	N	54.36	-0.50	34.56	0.15
Plate B			122	95			56.56	-0.58	26.32	-0.14
Avg			158.5	292			55.46	-0.54	30.44	0.01
Plate A	XM_001134465	OTUD1	265	233	N	N	44.53	-0.92	38.20	0.32
Plate B			302	150			51.66	-0.77	33.33	0.17
Avg			283.5	191.5			48.09	-0.84	35.77	0.25
Plate A	NM_017493	OTUD4	465	535	N	N	80.22	0.59	24.49	-0.34
Plate B			323	241			86.69	0.60	23.65	-0.25
Avg			394	388			83.45	0.60	24.07	-0.30
Plate A	NM_017602	OTUD5	190	95	N	N	74.74	0.36	62.11	1.48
Plate B			62	50			82.26	0.43	40.00	0.47
Avg			126	72.5			78.50	0.39	51.05	0.97
Plate A	NM_016023	OTUD6B	576	555	N	N	84.20	0.76	34.05	0.12
Plate B			329	274			78.72	0.29	24.45	-0.22
Avg			452.5	414.5			81.46	0.52	29.25	-0.05
Plate A	NM_130901	OTUD7	82	76	N	N	92.68	1.12	86.84	2.67
Plate B			87	118			87.36	0.63	92.37	2.80
Avg			84.5	97			90.02	0.87	89.61	2.73
Plate A	NM_020205	ZA20D1	195	269	N	N	63.08	-0.13	53.16	1.05
Plate B			131	214			33.59	-1.48	43.46	0.62
Avg			163	241.5			48.33	-0.81	48.31	0.84
Plate A	NM_020367	PARP11	463	635	N	N	36.93	-0.44	9.13	-1.08
Plate B			354	351			42.66	-0.84	14.53	-0.66
Avg			408.5	493			39.79	-0.64	11.83	-0.87
Plate A	NM_006445	PRPF8	43	52	N	N	55.81	-2.07	53.85	1.08
Plate B			20	12			50.00	-2.58	83.33	2.39
Avg			31.5	32			52.91	-2.33	68.59	1.74
Plate A	NM_005805	PSMD14	58	88	Y	N	17.24	0.55	12.50	-0.91
Plate B			18	40			5.56	0.88	37.50	0.36
Avg			38	64			11.40	0.71	25.00	-0.28
Plate A	NM_138334	SBBI54	529	542	N	N	79.21	-0.34	58.86	1.32
Plate B			192	179			93.75	-0.42	69.27	1.77
Avg			360.5	360.5			86.48	-0.38	64.06	1.55
Plate A	NM_021627	SENP2	292	600	N	N	58.22	-1.25	34.00	0.12
Plate B			360	174			60.56	-1.82	40.80	0.51
Avg			326	387			59.39	-1.54	37.40	0.31
Plate A	NM_201647	STAMBP	841	921	N	N	36.62	-0.00	11.62	-0.96
Plate B			542	537			24.91	-0.54	14.71	-0.65
Avg			691.5	729			30.77	-0.27	13.16	-0.80
Plate A	NM_020799	STAMBPL1	328	383	N	N	66.16	0.30	18.80	-0.61
Plate B			139	171			57.55	-0.35	24.56	-0.21
Avg			233.5	277			61.86	-0.03	21.68	-0.41
Plate A	NM_006290	TNFA1P3	367	531	N	N	73.30	0.30	79.10	2.30
Plate B			239	176			62.34	-0.35	80.68	2.28
Avg			303	353.5			67.82	-0.03	79.89	2.29
Plate A	NM_007106	UBL3	312	409	N	N	59.94	-1.52	23.23	-0.40
Plate B			146	367			71.23	-1.92	18.53	-0.48
Avg			229	388			65.58	-1.72	20.88	-0.44
Plate A	NM_014235	UBL4	600	683	N	N	30.17	-1.24	10.54	-1.01
Plate B			249	455			22.49	-1.12	7.91	-0.95
Avg			424.5	569			26.33	-1.18	9.23	-0.98
Plate A	NM_001048241	UBL5	17	51	Y	N	29.41	-1.56	37.25	0.28
Plate B			12	8			41.67	-1.16	37.50	0.36
Avg			14.5	29.5			35.54	-1.36	37.38	0.32
Plate A	NM_174916	UBR1	582	652	N	N	49.14	-0.72	18.40	-0.63
Plate B			156	402			62.18	-0.36	24.13	-0.23
Avg			369	527			55.66	-0.54	21.27	-0.43
Plate A	NM_024954	UBTD1	400	677	N	N	35.25	-1.31	8.12	-1.13
Plate B			257	284			35.02	-1.42	8.80	-0.91
Avg			328.5	480.5			35.13	-1.37	8.46	-1.02
Plate A	NM_152277	DC-UBP	367	179	N	N	81.20	0.63	58.66	1.31
Plate B			99	128			94.95	0.93	45.31	0.71
Avg			233	153.5			88.07	0.78	51.99	1.01
Plate A	NM_004181	UCHL1	298	459	N	N	66.78	0.02	31.15	-0.02
Plate B			312	245			78.53	0.28	33.06	0.16
Avg			305	352			72.65	0.15	32.11	0.07
Plate A	NM_006002	UCHL3	715	719	N	N	52.87	-0.57	15.58	-0.77
Plate B			226	253			52.21	-0.75	17.00	-0.55
Avg			470.5	486			52.54	-0.66	16.29	-0.66
Plate A	NM_015984	UCHL5	490	423	N	N	72.65	0.27	26.48	-0.24
Plate B			110	161			72.73	0.05	26.09	-0.15
Avg			300	292			72.69	0.16	26.28	-0.19
Plate A	NM_012474	UMPK	447	586	N	N	71.36	0.22	38.40	0.33
Plate B			379	452			57.78	-0.53	30.97	0.07
Avg			413	519			64.57	-0.16	34.68	0.20
Plate A	NM_018314	UEVLD	311	766	N	N	89.71	0.99	41.12	0.47
Plate B			139	202			86.33	0.59	43.56	0.63
Avg			225	484			88.02	0.79	42.34	0.55
Plate A	NM_001035247	UFD1L	252	393	N	N	63.49	-0.12	39.69	0.40
Plate B			264	350			41.67	-1.16	26.29	-0.14
Avg			258	371.5			52.58	-0.64	32.99	0.13
Plate A	NM_001017416	USP1	244	411	N	N	84.84	0.78	29.44	-0.10
Plate B			174	131			82.76	0.45	19.85	-0.42
Avg			209	271			83.80	0.62	24.64	-0.26
Plate A	NM_005153	USP10	387	362	N	N	44.19	-0.93	18.78	-0.61
Plate B			195	375			38.97	-1.27	24.80	-0.20
Avg			291	368.5			41.58	-1.10	21.79	-0.41
Plate A	NM_004651	USP11	430	369	N	N	58.14	-0.34	18.16	-0.64
Plate B			99	233			61.62	-0.38	19.31	-0.45
Avg			264.5	301			59.88	-0.36	18.74	-0.54
Plate A	NM_182488	USP12	547	985	N	N	8.23	-2.45	8.83	-1.09
Plate B			293	308			4.10	-2.64	13.64	-0.70
Avg			420	646.5			6.16	-2.54	11.23	-0.90
Plate A	NM_003940	USP13	610	583	N	N	32.30	-1.43	8.75	-1.10
Plate B			198	254			38.38	-1.29	16.14	-0.59
Avg			404	418.5			35.34	-1.36	12.44	-0.84
Plate A	NM_001037334	USP14	336	344	N	N	83.63	0.73	50.58	0.92
Plate B			202	162			81.68	0.41	49.38	0.89
Avg			269	253			82.66	0.57	49.98	0.90
Plate A	NM_006313	USP15	581	667	N	N	58.52	-0.33	22.34	-0.44
Plate B			212	292			68.87	-0.10	27.74	-0.07
Avg			396.5	479.5			63.69	-0.21	25.04	-0.26
Plate A	NM_001001992	USP16	385	484	N	N	71.43	0.22	21.69	-0.47
Plate B			247	161			55.47	-0.62	42.86	0.60
Avg			316	322.5			63.45	-0.20	32.28	0.06
Plate A	NM_001105662	USP17	259	473	N	N	92.66	1.12	77.59	2.22
Plate B			389	284			91.00	0.77	75.00	2.02
Avg			324	378.5			91.83	0.94	76.29	2.12
Plate A	NM_017414	USP18	466	502	N	N	84.12	0.75	45.62	0.68
Plate B			607	303			76.11	0.19	40.26	0.48
Avg			536.5	402.5			80.12	0.47	42.94	0.58
Plate A	NM_006677	USP19	566	730	N	N	17.31	-2.07	5.21	-1.27
Plate B			477	371			14.05	-2.25	1.35	-1.25
Avg			521.5	550.5			15.68	-2.16	3.28	-1.26
Plate A	NM_171997	USP2	543	384	N	N	71.64	0.23	38.28	0.33
Plate B			212	329			81.13	0.38	29.18	-0.01
Avg			377.5	356.5			76.39	0.31	33.73	0.16
Plate A	NM_001110303	USP20	507	666	N	N	56.02	-0.43	28.08	-0.16
Plate B			382	222			74.35	0.12	28.83	-0.03
Avg			444.5	444			65.18	-0.16	28.45	-0.09
Plate A	NM_012475	USP21	195	243	N	N	66.15	-0.00	37.86	0.31
Plate B			57	124			71.93	0.02	31.45	0.09
Avg			126	183.5			69.04	0.01	34.66	0.20
Plate A	NM_015276	USP22	589	727	N	N	12.39	-2.27	3.99	-1.33
Plate B			291	246			5.15	-2.60	7.32	-0.98
Avg			440	486.5			8.77	-2.44	5.65	-1.15
Plate A	NM_015306	USP24	222	387	N	N	57.66	-0.36	44.19	0.61
Plate B			190	296			72.63	0.05	40.88	0.51
Avg			206	341.5			65.14	-0.16	42.53	0.56
Plate A	NM_013396	USP25	171	356	N	N	78.36	0.51	60.67	1.41
Plate B			112	141			96.43	0.98	66.67	1.65
Avg			141.5	248.5			87.40	0.75	63.67	1.53
Plate A	NM_031907	USP26	745	594	N	N	24.56	-1.76	9.60	-1.06
Plate B			704	545			18.89	-2.06	9.91	-0.87
Avg			724.5	569.5			21.73	-1.91	9.75	-0.96
Plate A	XM_372213	USP27X	385	598	N	N	36.88	-1.24	29.77	-0.08
Plate B			275	601			58.91	-0.49	20.30	-0.40
Avg			330	599.5			47.90	-0.86	25.03	-0.24
Plate A	NM_020886	USP28	504	605	N	N	77.38	0.47	33.72	0.11
Plate B			283	355			66.78	-0.18	25.35	-0.18
Avg			393.5	480			72.08	0.15	29.54	-0.04
Plate A	NM_020903	USP29	648	1324	N	N	39.66	-1.12	13.29	-0.88
Plate B			386	234			30.57	-1.60	7.69	-0.96
Avg			517	779			35.12	-1.36	10.49	-0.92
Plate A	NM_006537	USP3	374	579	N	N	61.76	-0.19	28.32	-0.15
Plate B			247	180			58.70	-0.50	18.89	-0.47
Avg			310.5	379.5			60.23	-0.34	23.61	-0.31
Plate A	NM_032663	USP30	300	94	N	N	74.00	0.33	51.06	0.95
Plate B			49	35			71.43	0.00	20.00	-0.42
Avg			174.5	64.5			72.71	0.17	35.53	0.26
Plate A	NM_020718	USP31	309	308	N	N	4.85	-2.59	1.95	-1.42
Plate B			211	193			1.90	-2.72	1.55	-1.24
Avg			260	250.5			3.38	-2.66	1.75	-1.33
Plate A	NM_032582	USP32	562	407	N	N	10.85	-2.34	4.67	-1.29
Plate B			376	491			13.03	-2.29	6.52	-1.02
Avg			469	449			11.94	-2.31	5.59	-1.15
Plate A	NM_015017	USP33	493	434	N	N	77.69	0.48	15.44	-0.77
Plate B			367	346			84.74	0.53	14.45	-0.66
Avg			430	390			81.21	0.50	14.94	-0.72
Plate A	NM_014709	USP34	638	501	N	N	46.08	-0.85	14.37	-0.82
Plate B			220	241			72.27	0.04	15.35	-0.62
Avg			429	371			59.18	-0.41	14.86	-0.72
Plate A	NM_020798	USP35	429	554	N	N	76.92	0.45	31.77	0.01
Plate B			228	300			80.26	0.35	38.00	0.38
Avg			328.5	427			78.59	0.40	34.88	0.20
Plate A	NM_025090	USP36	494	618	N	N	51.21	-0.64	11.49	-0.96
Plate B			137	386			52.55	-0.74	10.88	-0.82
Avg			315.5	502			51.88	-0.69	11.18	-0.89
Plate A	NM_020935	USP37	536	467	N	N	48.88	-0.73	26.77	-0.23
Plate B			585	309			66.15	-0.20	33.98	0.20
Avg			560.5	388			57.52	-0.47	30.37	-0.01
Plate A	NM_032557	USP38	121	64	N	N	79.34	0.55	46.88	0.74
Plate B			53	75			94.34	0.90	62.67	1.48
Avg			87	69.5			86.84	0.73	54.77	1.11
Plate A	NM_006590	USP39	7	3	Y	N	71.43	0.22	33.33	0.09
Plate B			7	4			71.43	0.00	100.00	3.13
Avg			7	3.5			71.43	0.11	66.67	1.61
Plate A	NM_003363	USP4	302	255	N	N	58.61	-0.32	7.45	-1.16
Plate B			57	187			42.11	-1.15	10.16	-0.85
Avg			179.5	221			50.36	-0.73	8.81	-1.01
Plate A	NM_018218	USP40	67	80	N	N	76.12	0.42	57.50	1.26
Plate B			27	74			74.07	0.11	40.54	0.49
Avg			47	77			75.10	0.26	49.02	0.88
Plate A	XM_937988	USP41	91	110	N	N	39.56	-1.13	22.73	-0.42
Plate B			49	34			53.06	-0.72	5.88	-1.04
Avg			70	72			46.31	-0.92	14.30	-0.73
Plate A	NM_032172	USP42	160	270	N	N	97.50	1.32	60.37	1.39
Plate B			118	221			92.37	0.83	54.75	1.13
Avg			139	245.5			94.94	1.07	57.56	1.26
Plate A	NM_153210	USP43	39	46	N	N	51.28	-0.63	36.96	0.26
Plate B			43	43			88.37	0.67	44.19	0.66
Avg			41	44.5			69.83	0.02	40.57	0.46
Plate A	NM_001042403	USP44	268	327	N	N	78.73	0.53	48.01	0.80
Plate B			95	223			71.58	0.01	50.67	0.94
Avg			181.5	275			75.16	0.27	49.34	0.87
Plate A	NM_001080481	USP45	115	88	N	N	96.52	1.28	44.32	0.62
Plate B			107	27			91.59	0.79	70.37	1.82
Avg			111	57.5			94.06	1.04	57.34	1.22
Plate A	NM_022832	USP46	371	580	N	N	88.14	0.92	14.83	-0.80
Plate B			366	629			86.07	0.58	24.80	-0.20
Avg			368.5	604.5			87.10	0.75	19.81	-0.50
Plate A	NM_022832	USP46	643	474	N	N	54.74	1.03	10.55	0.10
Plate B			290	402			60.34	1.55	7.71	-0.16
Avg			466.5	438			57.54	1.29	9.13	-0.03
Plate A	NM_017944	USP47	221	220	N	N	84.16	2.18	25.91	1.23
Plate B			162	146			80.86	2.42	23.97	0.92
Avg			191.5	183			82.51	2.30	24.94	1.07
Plate A	NM_001032730	USP48	588	286	N	N	92.69	2.51	13.99	0.35
Plate B			95	299			83.16	2.52	22.07	0.79
Avg			341.5	292.5			87.92	2.51	18.03	0.57
Plate A	NM_018561	USP49	61	115	N	N	88.52	2.34	62.61	3.92
Plate B			154	83			66.88	1.83	84.34	4.91
Avg			107.5	99			77.70	2.09	73.47	4.42
Plate A	NM_003481	USP5	523	399	N	N	66.54	1.49	36.84	2.03
Plate B			325	327			57.85	1.44	36.09	1.72
Avg			424	363			62.19	1.47	36.46	1.87
Plate A	NM_203494	USP50	260	342	N	N	85.00	2.21	69.59	4.43
Plate B			200	206			79.50	2.36	46.12	2.38
Avg			230	274			82.25	2.29	57.85	3.41
Plate A	NM_201286	USP51	469	616	N	N	67.38	1.52	33.93	1.81
Plate B			265	425			50.57	1.13	42.82	2.17
Avg			367	520.5			58.97	1.33	38.38	1.99
Plate A	NM_014871	USP52	384	606	N	N	48.18	0.78	6.77	-0.18
Plate B			538	448			18.40	-0.24	6.47	-0.24
Avg			461	527			33.29	0.27	6.62	-0.21
Plate A	NM_019050	USP53	311	444	N	N	21.86	-0.24	6.53	-0.20
Plate B			457	510			4.38	-0.83	5.10	-0.33
Avg			384	477			13.12	-0.54	5.81	-0.27
Plate A	NM_152586	USP54	86	176	N	N	45.35	0.67	59.09	3.66
Plate B			184	55			48.91	1.06	63.64	3.54
Avg			135	115.5			47.13	0.87	61.36	3.60
Plate A	NM_004505	USP6	145	307	N	N	62.76	1.35	12.70	0.26
Plate B			146	102			73.97	2.13	29.41	1.28
Avg			145.5	204.5			68.37	1.74	21.06	0.77
Plate A	NM_003470	USP7	147	190	N	N	93.88	2.55	48.95	2.92
Plate B			106	81			66.04	1.79	49.38	2.60
Avg			126.5	135.5			79.96	2.17	49.17	2.76
Plate A	NM_005154	USP8	482	651	N	N	25.93	-0.08	6.14	-0.23
Plate B			246	286			15.85	-0.34	9.09	-0.07
Avg			364	468.5			20.89	-0.21	7.62	-0.15
Plate A	NM_001039591	USP9X	595	339	N	N	46.22	0.70	23.01	1.01
Plate B			406	209			50.74	1.14	22.01	0.79
Avg			500.5	274			48.48	0.92	22.51	0.90
Plate A	NM_004654	USP9Y	418	679	N	N	18.42	-0.37	4.86	-0.32
Plate B			426	269			12.68	-0.48	7.06	-0.20
Avg			422	474			15.55	-0.43	5.96	-0.26
Plate A	NM_005800	C13ORF22	168	233	N	N	71.43	1.68	9.87	0.05
Plate B			90	199			47.78	1.01	9.05	-0.07
Avg			129	216			59.60	1.35	9.46	-0.01
Plate A	NM_025054	VCPIP1	214	444	N	N	40.65	0.49	18.92	0.71
Plate B			333	153			19.82	-0.18	22.88	0.84
Avg			273.5	298.5			30.24	0.16	20.90	0.78
Plate A	NM_018566	YOD1	239	431	N	N	60.67	1.26	25.29	1.18
Plate B			251	217			64.94	1.75	24.42	0.95
Avg			245	324			62.80	1.50	24.86	1.06
Plate A	NM_020205	ZA20D1	184	264	N	N	34.24	0.24	15.91	0.49
Plate B			135	111			20.00	-0.17	17.12	0.46
Avg			159.5	187.5			27.12	0.04	16.51	0.48
Plate A	NM_017580	ZRANB1	201	349	N	N	89.55	2.38	38.11	2.12
Plate B			193	204			87.56	2.71	47.55	2.48
Avg			197	276.5			88.56	2.55	42.83	2.30

