# Supplementary material for: A High-Content RNAi Screen Identifies Ubiquitin Modifiers That Regulate TNF-Dependent Nuclear Accumulation of NF-κB
Source: Front Immunol. 2014 Jul 14;5:322. doi: 10.3389/fimmu.2014.00322 (PMC4094887; doi:10.3389/fimmu.2014.00322)
Supplement: Supplementary file 1 [file Presentation_1.ZIP › Supp. Table 5.PDF]

GENE	A549 30MIN siRNA1	A549 30MIN siRNA2	A549 30MIN siRNA3	A549 30MIN siRNA4	HeLa 30MIN siRNA1	HeLa 30MIN siRNA2	HeLa 30MIN siRNA3	HeLa 30MIN siRNA4	A549 120MIN siRNA1	A549 120MIN siRNA2	A549 120MIN siRNA3	A549 120MIN siRNA4	HeLa 120MIN siRNA1	HeLa 120MIN siRNA2	HeLa 120MIN siRNA3	HeLa 120MIN siRNA4
CUL1	-0.93	-1.39	-3.50	-3.53	-5.54	-0.12	-9.57	-2.07	-1.58	-0.87	-8.03	-7.94	1.02	2.62	-0.79	1.31
DCUN1D	0.42	0.65	-0.22	-0.07	2.37	1.15	1.29	-3.20	-4.92	-0.72	-3.31	-2.65	2.70	2.41	2.11	0.31
FBXW11	-2.31	-1.39	-3.50	-3.53	-10.88	-9.76	-6.23	-0.25	-3.81	-5.50	-4.99	0.79	-0.54	-0.67	1.38	3.84
UBE2V1	0.98	-0.99	-1.07	-0.23	1.04	1.15	1.83	2.00	-0.84	-6.10	-2.51	-1.91	2.13	2.32	3.12	4.56
FBXO36	-0.31	-0.52	-0.85	0.55	2.26	2.43	0.59	-0.96	0.27	-0.53	2.21	5.01	4.20	4.24	2.07	2.44
FBXO8	1.09	-0.57	-0.42	-1.30	2.56	1.66	-0.22	2.07	-0.83	-6.84	-0.09	-7.77	4.89	2.42	2.33	0.88
TRIM17	-0.70	-2.06	0.04	0.45	0.26	-3.02	-0.54	0.31	-4.56	-1.79	1.74	0.77	1.19	3.29	1.58	1.95
MLL2	0.82	0.29	-0.88	-1.12	1.62	-4.56	-2.15	-2.84	3.16	-3.39	-5.07	-3.39	1.60	0.97	1.98	0.35
KRTAP5-9	-0.70	0.10	-0.48	-0.08	2.33	0.97	-0.04	-4.49	-3.53	2.19	-3.76	4.46	2.43	2.59	0.82	1.70
RNF31	0.43	-1.85	-1.52	-1.88	1.28	-3.37	-4.62	0.33	2.85	-6.41	1.08	-3.84	1.20	-0.13	-0.35	2.76
USP12	-0.60	-0.66	0.34	-0.49	2.59	-6.94	-6.68	-1.35	-0.32	-0.25	1.34	3.06	3.45	0.14	0.30	0.57
A20	0.27	-1.10	-1.53	-1.02	2.76	0.05	2.65	2.26	2.19	-3.83	2.59	0.14	4.89	4.53	4.99	4.98
USP31	-0.46	-4.56	-1.11	1.13	2.54	-11.17	2.27	-0.38	-0.58	-12.44	-7.14	8.33	2.20	-2.56	4.85	1.27
USP26	0.11	-1.80	-1.63	-1.00	2.39	1.78	1.47	-0.06	-3.19	-10.23	-3.85	-4.82	3.33	4.21	2.02	0.97
USP50	-0.76	-1.61	0.04	0.95	0.81	-3.44	1.59	2.31	0.04	-4.59	3.71	6.49	0.96	-0.18	1.83	4.32
USP49	0.39	-0.44	0.15	-0.86	2.01	2.24	1.12	0.84	2.41	-4.00	2.24	-4.49	2.13	4.11	3.85	2.07
USP7	-0.34	-0.16	-0.48	0.64	1.58	2.43	0.83	2.19	0.32	-2.01	0.26	2.42	3.41	4.71	1.69	3.01
USP54	-0.08	-3.50	-1.35	0.42	1.97	-9.33	1.96	1.97	2.75	-11.27	-7.72	2.93	2.88	-1.37	4.11	4.06
