# Supplementary material for: A High-Content RNAi Screen Identifies Ubiquitin Modifiers That Regulate TNF-Dependent Nuclear Accumulation of NF-κB
Source: Front Immunol. 2014 Jul 14;5:322. doi: 10.3389/fimmu.2014.00322 (PMC4094887; doi:10.3389/fimmu.2014.00322)
Supplement: Supplementary file 1 [file Presentation_1.ZIP › Supp. Tables legends.pdf]

Legends Supplementary Tables

Supplementary tables 1-4: Complete results from the primary siRNA screen. The number of cells counted for each sample at 30 and 120min TNF are shown along with the corresponding percentage of cells with nuclear NF-κB and the respective Z scores. siRNAs that appeared to give lethal or nuclear phenotypes are indicated in the respective columns. Table 1 contains results for Cullins, E1, E2, and HECT E3 ligases. Table 2 contains results for F-box and SOCS box E3 ligases. Table 3 contains the results for RING finger and RING finger-like E3 ligases.

Supplementary table 5: All results from the validation screen described in Figure 6. Cell type, siRNA number and duration of TNF exposure are indicated for each gene. Scores indicate the number of standard deviations from control non-silencing siRNA duplexes.
