# Supplementary material for: A High-Content RNAi Screen Identifies Ubiquitin Modifiers That Regulate TNF-Dependent Nuclear Accumulation of NF-κB
Source: Front Immunol. 2014 Jul 14;5:322. doi: 10.3389/fimmu.2014.00322 (PMC4094887; doi:10.3389/fimmu.2014.00322)
Supplement: Supplementary file 1 [file Presentation_1.ZIP › Supp. Table 1.PDF]

	Gene ID	Gene	30 mins # of cells	120 mins # of cells	Lethal	Nuclear phenotype	30 mins % NF-kB Translocation	30 mins Z-score	120 mins % NF-kB Translocation	120 mins Z-score
Plate A	NM_181803	UBE2C	964	1206	N	N	97.61	0.61	76.62	0.92
Plate B			1188	999			97.73	0.55	82.68	1.16
Avg			1076	1102.5			97.67	0.58	79.65	1.04
Plate A	NM_181349	SMURF1	649	857	N	N	88.14	−0.34	80.98	1.17
Plate B			802	285			92.52	0.06	81.05	1.06
Avg			725.5	571			90.33	−0.14	81.02	1.12
Plate A	NM_015902	EDD1	383	522	N	N	85.38	−0.62	39.85	−1.21
Plate B			669	515			81.17	−0.99	48.35	−0.96
Avg			526	518.5			83.27	−0.80	44.10	−1.08
Plate A	NM_0011111	HIP2	434	269	N	N	94.01	0.25	92.94	1.86
Plate B			788	545			92.01	0.02	95.96	1.98
Avg			611	407			93.01	0.13	94.45	1.92
Plate A	NM_015382	HECTD1	390	251	N	N	98.72	0.72	76.89	0.93
Plate B			785	400			98.60	0.63	78.00	0.87
Avg			587.5	325.5			98.66	0.67	77.45	0.90
Plate A	NM_014176	UBE2T	440	202	N	N	85.91	−0.56	76.73	0.92
Plate B			426	258			90.61	−0.11	68.60	0.29
Avg			433	230			88.26	−0.34	72.67	0.61
Plate A	NM_004359	CDC34	215	210	N	N	91.63	0.01	45.71	−0.87
Plate B			319	460			91.22	−0.06	72.17	0.51
Avg			267	335			91.43	−0.02	58.94	−0.18
Plate A	NM_0011096	FLJ34154	550	837	N	N	96.18	0.46	85.66	1.44
Plate B			375	712			96.53	0.44	85.67	1.35
Avg			462.5	774.5			96.36	0.45	85.67	1.39
Plate A	NM_020640	DCUN1D1	388	606	N	N	94.33	0.28	63.37	0.15
Plate B			600	563			98.83	0.65	71.58	0.48
Avg			494	584.5			96.58	0.47	67.47	0.31
Plate A	NM_003591	CUL2	271	244	N	N	92.25	0.07	71.72	0.63
Plate B			274	326			96.72	0.45	76.99	0.81
Avg			272.5	285			94.48	0.26	74.36	0.72
Plate A	NM_014606	HERC3	806	775	N	N	82.26	−0.93	68.26	0.43
Plate B			626	604			86.74	−0.47	64.40	0.03
Avg			716	689.5			84.50	−0.70	66.33	0.23
Plate A	NM_018299	UBE2W	701	751	N	N	90.01	−0.15	40.88	−1.15
Plate B			560	679			92.68	0.08	56.41	−0.46
Avg			630.5	715			91.35	−0.04	48.64	−0.80
Plate A	NM_003350	UBE2V2	310	765	N	N	87.42	−0.41	83.27	1.30
Plate B			704	486			92.19	0.03	85.39	1.33
Avg			507	625.5			89.80	−0.19	84.33	1.32
Plate A	NM_032299	DCUN1D5	873	967	N	N	86.03	−0.55	60.29	−0.03
Plate B			935	939			77.33	−1.35	60.81	−0.19
Avg			904	953			81.68	−0.95	60.55	−0.11
Plate A	NM_004667	HERC2	533	715	N	N	94.37	0.28	51.89	−0.51
Plate B			516	938			91.67	−0.02	52.88	−0.68
Avg			524.5	826.5			93.02	0.13	52.38	−0.60
Plate A	NM_003348	UBE2N	317	322	N	N	80.76	−1.08	66.46	0.33
Plate B			169	289			60.95	−2.88	60.21	−0.23
Avg			243	305.5			70.85	−1.98	63.33	0.05
Plate A	NM_023079	UBE2Z	464	707	N	N	80.39	−1.11	46.96	−0.80
Plate B			626	899			84.35	−0.70	48.28	−0.96
Avg			545	803			82.37	−0.91	47.62	−0.88
Plate A	NM_003347	UBE2L3	390	766	N	N	98.72	0.72	91.25	1.76
Plate B			351	544			91.45	−0.04	89.34	1.57
Avg			370.5	655			95.09	0.34	90.30	1.67
Plate A	NM_016323	HERC5	245	260	N	N	95.92	0.44	81.54	1.20
Plate B			304	344			92.43	0.06	86.92	1.42
Avg			274.5	302			94.18	0.25	84.23	1.31
Plate A	NM_0010129	UBE2NL	180	454	N	N	94.44	0.29	23.35	−2.16
Plate B			391	326			96.16	0.40	29.75	−2.11
Avg			285.5	390			95.30	0.35	26.55	−2.13
Plate A	NM_173475	DCUN1D3	570	684	N	N	98.07	0.65	75.00	0.82
Plate B			417	166			98.08	0.58	86.75	1.41
Avg			493.5	425			98.08	0.62	80.87	1.12
Plate A	NM_016252	BIRC6	858	809	N	N	85.43	−0.61	40.42	−1.17
Plate B			740	707			88.11	−0.35	44.70	−1.18
Avg			799	758			86.77	−0.48	42.56	−1.18
Plate A	NM_194457	UBE2J2	1217	1153	N	N	89.24	−0.23	64.70	0.23
Plate B			1178	642			88.88	−0.28	63.71	−0.01
Avg			1197.5	897.5			89.06	−0.25	64.20	0.11
Plate A	NM_015052	HECW1	1090	786	N	N	97.80	0.63	63.99	0.19
Plate B			900	676			98.00	0.57	62.13	−0.11
Avg			995	731			97.90	0.60	63.06	0.04
Plate A	NM_003335	UBE1L	988	1220	N	N	70.34	−2.12	52.30	−0.49
Plate B			826	883			69.13	−2.11	49.26	−0.90
Avg			907	1051.5			69.74	−2.12	50.78	−0.70
Plate A	NM_153280	UBE1	741	697	N	N	95.01	0.35	53.95	−0.39
Plate B			933	840			95.07	0.30	67.74	0.24
Avg			837	768.5			95.04	0.32	60.84	−0.08
Plate A	NM_003922	HERC1	326	393	N	N	88.04	−0.35	47.07	−0.79
Plate B			264	201			94.70	0.27	59.20	−0.29
Avg			295	297			91.37	−0.04	53.14	−0.54
Plate A	NM_020771	HACE1	228	458	N	N	90.79	−0.07	70.09	0.54
Plate B			213	253			88.73	−0.29	74.70	0.67
Avg			220.5	355.5			89.76	−0.18	72.40	0.60
Plate A	NM_014780	CUL7	384	318	N	N	93.75	0.22	70.13	0.54
Plate B			226	311			92.04	0.02	70.42	0.40
Avg			305	314.5			92.89	0.12	70.27	0.47
Plate A	NM_014501	UBE2S	462	720	N	N	98.27	0.67	64.72	0.23
Plate B			664	813			94.13	0.21	63.10	−0.05
Avg			563	766.5			96.20	0.44	63.91	0.09
Plate A	NM_003590	CUL3	867	570	N	N	89.16	−0.24	80.00	1.11
Plate B			785	624			85.73	−0.57	87.02	1.43
Avg			826	597			87.45	−0.40	83.51	1.27
Plate A	NM_031483	ITCH	587	401	N	N	93.36	0.18	65.59	0.28
Plate B			377	470			92.57	0.07	62.34	−0.09
Avg			482	435.5			92.96	0.13	63.96	0.09
Plate A	NM_031407	HUWE1	1085	925	N	N	84.42	−0.71	61.62	0.05
Plate B			595	1281			77.14	−1.37	70.88	0.43
Avg			840	1103			80.78	−1.04	66.25	0.24
Plate A	NM_012298	CAND2	269	492	N	N	88.48	−0.31	68.50	0.45
Plate B			256	404			86.72	−0.48	78.47	0.90
Avg			262.5	448			87.60	−0.39	73.48	0.67
Plate A	NM_181893	UBE2D3	616	555	N	N	73.86	−1.77	59.46	−0.07
Plate B			258	195			63.18	−2.67	63.08	−0.05
Avg			437	375			68.52	−2.22	61.27	−0.06
Plate A	NM_006154	NEDD4	334	957	N	N	100.00	0.85	74.92	0.82
Plate B			643	844			97.05	0.49	70.14	0.39
Avg			488.5	900.5			98.52	0.67	72.53	0.60
Plate A	NM_198329	UBE1DC1	679	1068	N	N	83.95	−0.76	50.19	−0.61
Plate B			708	753			86.02	−0.54	51.13	−0.79
Avg			693.5	910.5			84.98	−0.65	50.66	−0.70
Plate A	NM_003969	UBE2M	378	297	N	N	84.66	−0.69	81.14	1.18
Plate B			368	405			85.87	−0.56	86.67	1.41
Avg			373	351			85.26	−0.62	83.91	1.29
Plate A	NM_018314	UEVLD	345	622	N	N	86.96	−0.46	59.97	−0.04
Plate B			230	545			87.83	−0.37	64.04	0.01
Avg			287.5	583.5			87.39	−0.42	62.00	−0.02
Plate A	NM_080678	UBE2F	497	154	N	N	98.19	0.67	59.09	−0.10
Plate B			486	548			94.44	0.24	73.54	0.60
Avg			491.5	351			96.32	0.45	66.32	0.25
Plate A	NM_0010142	DCUN1D2	835	943	N	N	41.32	−5.02	37.01	−1.37
Plate B			702	1017			34.05	−5.38	45.72	−1.12
Avg			768.5	980			37.68	−5.20	41.37	−1.25
Plate A	NM_003592	CUL1	859	335	N	N	47.96	−4.36	43.28	−1.01
Plate B			1071	463			55.28	−3.40	62.20	−0.10
Avg			965	399			51.62	−3.88	52.74	−0.56
Plate A	NM_130466	UBE3B	941	559	N	N	96.71	0.52	79.61	1.09
Plate B			975	1029			97.74	0.55	90.18	1.63
Avg			958	794			97.22	0.53	84.90	1.36
Plate A	NM_003336	UBE2A	656	972	N	N	64.79	−2.67	50.31	−0.60
Plate B			1216	648			92.93	0.10	65.90	0.12
Avg			936	810			78.86	−1.29	58.10	−0.24
Plate A	NM_152653	UBE2E2	818	726	N	N	93.40	0.19	35.95	−1.43
Plate B			1124	946			87.72	−0.38	56.66	−0.45
Avg			971	836			90.56	−0.10	46.31	−0.94
Plate A	NM_024602	HECTD3	389	402	N	N	79.95	−1.16	43.03	−1.02
Plate B			622	525			86.66	−0.48	50.48	−0.83
Avg			505.5	463.5			83.30	−0.82	46.76	−0.93
Plate A	NM_003345	UBE2I	408	630	N	N	98.04	0.65	67.62	0.40
Plate B			671	535			98.51	0.62	66.36	0.15
Avg			539.5	582.5			98.27	0.64	66.99	0.28
Plate A	NM_173469	UBE2Q2	338	443	N	N	94.38	0.28	58.47	−0.13
Plate B			288	289			91.32	−0.05	63.32	−0.03
Avg			313	366			92.85	0.12	60.89	−0.08
Plate A	NM_017912	HERC6	830	910	N	N	91.08	−0.04	41.43	−1.12
Plate B			493	1018			94.52	0.25	54.81	−0.56
Avg			661.5	964			92.80	0.10	48.12	−0.84
Plate A	NM_007013	WWP1	919	586	N	N	91.95	0.04	43.17	−1.02
Plate B			792	865			80.18	−1.09	45.09	−1.16
Avg			855.5	725.5			86.06	−0.52	44.13	−1.09
Plate A	NM_022066	UBE2O	955	724	N	N	93.72	0.22	54.14	−0.38
Plate B			828	1069			95.05	0.30	56.88	−0.43
Avg			891.5	896.5			94.38	0.26	55.51	−0.41
Plate A	NM_0010322	UBE2V1	433	293	N	N	97.46	0.59	97.27	2.11
Plate B			337	339			98.52	0.62	97.35	2.07
Avg			385	316			97.99	0.61	97.31	2.09
Plate A	NM_017811	UBE2R2	1058	892	N	N	89.70	−0.18	48.65	−0.70
Plate B			1150	1061			89.91	−0.18	49.01	−0.92
Avg			1104	976.5			89.81	−0.18	48.83	−0.81
Plate A	NM_022476	AKTIP	1037	1469	N	N	95.27	0.37	60.99	0.01
Plate B			195	836			94.36	0.23	66.27	0.15
Avg			616	1152.5			94.82	0.30	63.63	0.08
Plate A	NM_003341	UBE2E1	1072	937	N	N	94.78	0.32	92.00	1.81
Plate B			1278	854			96.40	0.43	96.49	2.01
Avg			1175	895.5			95.59	0.37	94.24	1.91
Plate A	NM_014671	UBE3C	554	1433	N	N	85.56	−0.60	75.85	0.87
Plate B			997	1280			88.47	−0.31	62.73	−0.07
Avg			775.5	1356.5			87.01	−0.46	69.29	0.40
Plate A	NM_006292	TSG101	668	1249	N	N	94.91	0.34	43.55	−0.99
Plate B			705	981			94.75	0.27	36.80	−1.67
Avg			686.5	1115			94.83	0.30	40.18	−1.33
Plate A	NM_003343	UBE2G2	265	616	N	N	84.53	−0.70	42.86	−1.03
Plate B			496	579			85.28	−0.61	40.24	−1.46
Avg			380.5	597.5			84.91	−0.66	41.55	−1.25
Plate A	NM_022739	SMURF2	1076	697	N	N	97.03	0.55	42.04	−1.08
Plate B			666	989			96.70	0.45	63.09	−0.05
Avg			871	843			96.86	0.50	52.57	−0.56
Plate A	NM_015277	NEDD4L	1133	1283	N	N	87.73	−0.38	40.14	−1.19
Plate B			1556	1319			87.98	−0.36	59.89	−0.25
Avg			1344.5	1301			87.86	−0.37	50.02	−0.72
Plate A	NM_003968	UBE1C	514	533	N	N	96.89	0.54	77.67	0.98
Plate B			542	219			94.28	0.23	84.93	1.30
Avg			528	376			95.58	0.38	81.30	1.14
Plate A	NM_006357	UBE2E3	474	760	N	N	86.50	−0.50	58.55	−0.13
Plate B			825	1134			84.24	−0.71	70.81	0.43
Avg			649.5	947			85.37	−0.61	64.68	0.15
Plate A	NM_003338	UBE2D1	1058	556	N	N	89.41	−0.21	48.20	−0.72
Plate B			1318	936			90.59	−0.12	60.79	−0.19
Avg			1188	746			90.00	−0.16	54.50	−0.46
Plate A	NM_000462	UBE3A	91	206	N	N	96.70	0.52	86.89	1.51
Plate B			376	161			97.87	0.56	84.47	1.27
Avg			233.5	183.5			97.29	0.54	85.68	1.39
Plate A	NM_004238	TRIP12	414	1063	N	N	88.65	−0.29	79.96	1.11
Plate B			961	699			89.07	−0.26	80.40	1.02
Avg			687.5	881			88.86	−0.27	80.18	1.07
Plate A	NM_173497	HECTD2	206	893	N	N	90.78	−0.08	51.85	−0.51
Plate B			1058	670			95.27	0.32	48.96	−0.92
Avg			632	781.5			93.03	0.12	50.40	−0.72
Plate A	NM_003589	CUL4A	638	1030	N	N	89.03	−0.25	60.49	−0.01
Plate B			764	1032			92.15	0.03	57.07	−0.42
Avg			701	1031			90.59	−0.11	58.78	−0.22
Plate A	NM_015601	HERC4	705	439	N	N	94.75	0.32	74.03	0.77
Plate B			958	744			96.35	0.42	78.63	0.91
Avg			831.5	591.5			95.55	0.37	76.33	0.84
Plate A	NM_020760	HECW2	511	759	N	N	93.35	0.18	23.32	−2.16
Plate B			1156	886			95.24	0.32	29.12	−2.15
Avg			833.5	822.5			94.29	0.25	26.22	−2.16
Plate A	NM_003337	UBE2B	895	799	N	N	93.41	0.19	42.80	−1.04
Plate B			779	1101			89.86	−0.18	52.04	−0.73
Avg			837	950			91.63	0.00	47.42	−0.88
Plate A	NM_152489	UBE2U	753	184	N	N	96.55	0.50	64.67	0.23
Plate B			304	483			80.26	−1.08	81.16	1.07
Avg			528.5	333.5			88.41	−0.29	72.92	0.65
Plate A	NM_003349	KUA-UEV	625	108	N	N	97.44	0.59	90.74	1.73
Plate B			344	166			98.55	0.62	90.36	1.64
Avg			484.5	137			97.99	0.61	90.55	1.69
Plate A	NM_018448	TIP120A	490	89	N	N	89.80	−0.17	49.44	−0.65
Plate B			471	206			85.56	−0.58	53.40	−0.65
Avg			480.5	147.5			87.68	−0.38	51.42	−0.65
Plate A	NM_0010394	KIAA0317	758	487	N	N	83.77	−0.78	42.51	−1.05
Plate B			730	512			81.23	−0.99	33.98	−1.85
Avg			744	499.5			82.50	−0.88	38.24	−1.45
Plate A	NM_015983	UBE2D4	534	616	N	N	97.94	0.64	57.63	−0.18
Plate B			521	455			99.23	0.69	57.36	−0.40
Avg			527.5	535.5			98.59	0.66	57.50	−0.29
Plate A	NM_015115	DCUN1D4	300	288	N	N	89.33	−0.22	30.90	−1.73
Plate B			697	464			83.36	−0.79	33.19	−1.90
Avg			498.5	376			86.35	−0.50	32.05	−1.81
Plate A	NM_0010798	CUL4B	734	591	N	N	72.21	−1.93	52.28	−0.49
Plate B			750	418			74.80	−1.59	58.13	−0.35
Avg			742	504.5			73.50	−1.76	55.21	−0.42
Plate A	NM_004223	UBE2L6	607	809	N	N	99.34	0.78	80.59	1.15
Plate B			743	467			97.58	0.53	67.88	0.25
Avg			675	638			98.46	0.66	74.24	0.70
Plate A	NM_016021	UBE2J1	503	571	N	N	96.22	0.47	65.67	0.29
Plate B			620	751			95.16	0.31	68.97	0.32
Avg			561.5	661			95.69	0.39	67.32	0.30
Plate A	XM_940609	FLJ25076	479	478	N	N	91.44	−0.01	78.66	1.04
Plate B			242	430			73.65	−1.69	80.70	1.04
Avg			360.5	454			82.54	−0.85	79.68	1.04
Plate A	NM_017582	UBE2Q1	580	475	N	N	98.45	0.43	64.42	0.56
Plate B			497	241			95.57	0.34	78.84	1.13
Avg			538.5	358			97.01	0.38	71.63	0.84
Plate A	NM_003342	UBE2G1	457	793	N	N	94.09	0.00	54.98	0.00
Plate B			371	389			93.26	0.00	56.56	−0.05
Avg			414	591			93.68	0.00	55.77	−0.02
Plate A	NM_182697	UBE2H	500	376	N	N	96.20	0.21	71.01	0.95
Plate B			487	529			98.77	0.81	77.50	1.06
Avg			493.5	452.5			97.48	0.51	74.26	1.00
Plate A	NM_003478	CUL5	758	774	N	N	96.17	0.20	78.55	1.40
Plate B			812	727			95.57	0.34	87.76	1.60
Avg			785	750.5			95.87	0.27	83.16	1.50
Plate A	NM_005744	ARIH1	670	568	N	N	93.73	−0.04	64.08	0.54
Plate B			244	382			87.70	−0.82	60.99	0.19
Avg			457	475			90.72	−0.43	62.54	0.36
Plate A	NM_003339	UBE2D2	337	670	N	N	73.59	−2.00	37.16	−1.06
Plate B			821	532			86.72	−0.96	50.75	−0.35
Avg			579	601			80.16	−1.48	43.96	−0.70
Plate A	NM_007014	WWP2	422	386	N	N	72.99	−2.06	25.39	−1.75
Plate B			509	455			81.53	−1.72	23.52	−1.79
Avg			465.5	420.5			77.26	−1.89	24.45	−1.77
Plate A	NM_153810	C10ORF46	723	462	N	N	95.30	0.12	50.87	−0.24
Plate B			600	712			95.17	0.28	57.44	0.00
Avg			661.5	587			95.23	0.20	54.15	−0.12
Plate A	NM_018227	UBE1L2	370	242	N	N	81.08	−1.27	45.87	−0.54
Plate B			279	358			79.57	−2.01	56.15	−0.07
Avg			324.5	300			80.33	−1.64	51.01	−0.30
